# Seleno Containing Compounds as Potent and Selective Antifungal Agents

**DOI:** 10.1021/acsinfecdis.2c00250

**Published:** 2022-08-19

**Authors:** Andrea Angeli, Alice Velluzzi, Silvia Selleri, Clemente Capasso, Costanza Spadini, Mattia Iannarelli, Clotilde S. Cabassi, Fabrizio Carta, Claudiu T. Supuran

**Affiliations:** † EUROFARBA Department, Sezione di Scienze Farmaceutiche e Nutraceutiche, University of Florence, Via Ugo Schiff 6, 50019 Sesto Fiorentino, Florence, Italy; ‡ Department of Biology, Agriculture and Food Sciences, Institute of Biosciences and Bioresources, 80131 Napoli, Italy; § Department of Veterinary Science, University of Parma, via del Taglio 10, 43126 Parma, Italy

**Keywords:** Fungi, Candida, Malassezia, Carbonic Anhydrases, Selenoureas, Antifungals

## Abstract

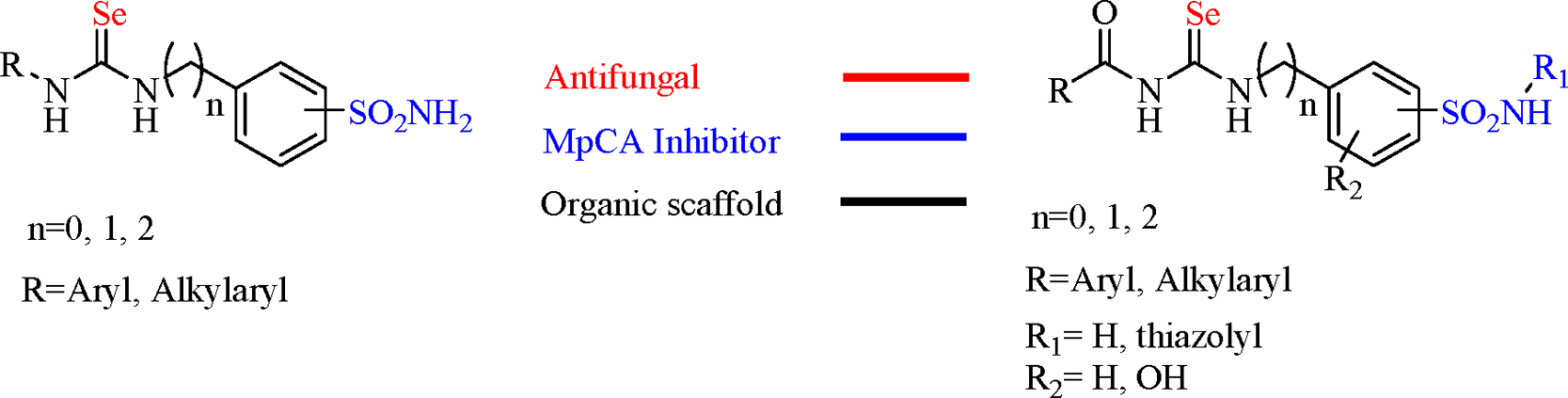

Fungal promoted infections are becoming a severe health global emergency due to drug-resistant phenomena and zoonosis. This work investigated compounds bearing acyl-/selenoureido moieties and primary/secondary sulfonamide groups as novel antifungal agents acting through organism-directed selenium toxicity and inhibition of the newly emergent therapeutic target, the Carbonic Anhydrases (CAs; EC 4.2.1.1). Reported data clearly indicate that seleno-containing scaffolds with respect to the standard-of-care drugs showed appreciable antifungal activity, which was suppressed when the chalcogen was replaced with its cognate isosteric elements sulfur and oxygen. In addition, such compounds showed excellent selectivity against *Malassezia pachydermatis* over its related genus strains *Malassezia furfur* and *Malassezia globosa*. Safe cytotoxicity profiles on bovine kidney cells (MDBK) and human HaCat cells, as well as the shallow hemolytic activity on defibrinated sheep blood, allowed us to consider these compounds as up-and-coming novel antifungals.

## Introduction

Fungal pathogens are largely present either in plants as well as animals and usually establish symbiotic relationships with the hosts. Dysregulation of such an equilibrium may result in significant consequences on human, animals, and plant welfare which in some cases may turn life-threatening.^1^ Metadata analyses at global scale account for constant and stiff on-growing cases of fungal-promoted infections in humans, which are dangerously close to well-known refractory diseases such as malaria and tuberculosis.^2^ Such fast evolution is mainly ascribed to the spread of relatively neglected infections which act as ideal incubators for organisms (i.e., fungi/yeasts) featured with highly variable genomes and quick reproduction rates. As a result, fungal strains resistant to classically used antifungal agents are rapidly selected.^3^ The extent and the rapid development of drug resistances triggered the World Health Organization (WHO) in 2021 to act in compiling the first fungal priority pathogens list (FPPL) of public health importance^4^ with the intent to create a boundary of sanitary protection for primarily exposed patients such as immunocompromised and/or neonatal, especially those receiving lipidic parenteral nutrition.^5^ Among the fungal pathogens of interest are the *Malassezia spp.*, which are involved in various skin diseases including pityriasis versicolor, seborrheic dermatitis, folliculitis, and dandruff. Specifically, *M. pachydermatis* holds a prominent role due to its constitutional presence in animals and its increasingly finding on human skin because of zoonotic transmission from domestic pets.^6,7^ Multiple cases of infections that require clinical attention refer to *M. pachydermatis* as the etiological agent,^6,7^ and it is reasonable to predict that these zoonoses will spread further in the coming years. Azole-containing compounds are commonly used to tackle *Malassezia spp*. either for systematic or topic administration, as they effectively interfere with the synthesis of fungal sterols by inhibiting the sterol-14-α-demethylase enzymes.^8^ Of exclusive topic administration is the inorganic SeS_2_, which acts on the sterol pathways by means of multiple and yet to be revealed mechanisms, all based on the metabolism of the selenium element.^9−11^ Although bacterial and fungal cells are far more efficient than the eukaryotic ones in processing such an element,^12−15^ the effectiveness of selenium-based drugs is closely related to the uptake capacity of the organism, which in turn is highly dependent on its lipidome constitution.^12−15^

In this context, we sought (i) to investigate whether the administration of selenium through its biologically fruitful organic form may affect its antifungal effectiveness and (ii) to make use of organic scaffolds bearing primary/secondary sulfonamide moieties with the intent to target the fungal expressed metalloenzyme carbonic anhydrases (CAs, E.C. 4.2.1.1). Such enzymes, among others, have a high potential to be validated as new druggable targets as they regulate key metabolic transformations linked to fungal survival and virulence.^16,17^

## Results and Discussion

### Design and Synthesis

**Scheme 1:**
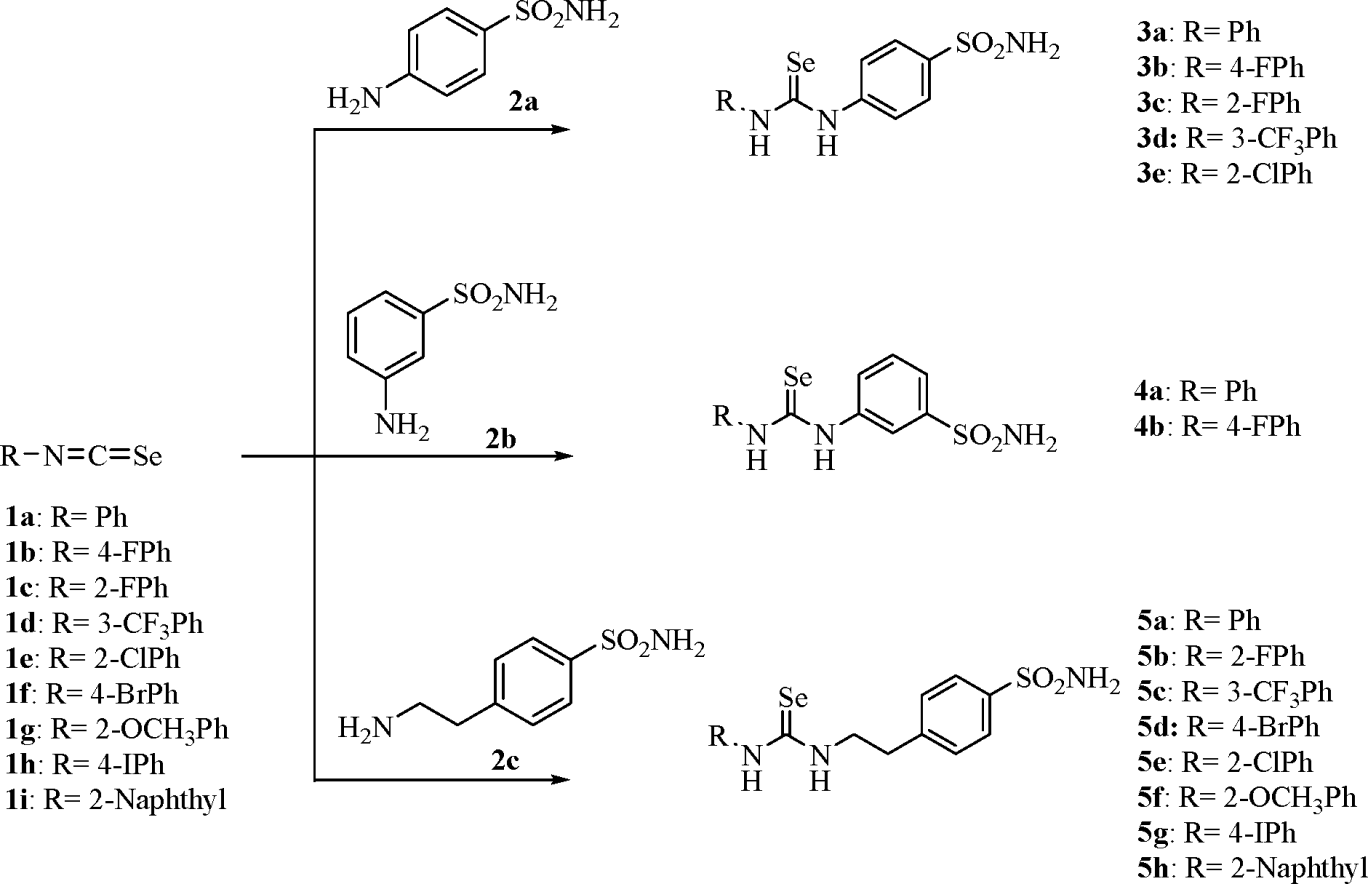
Synthesis of Selenoureas **3a**–**e**, **4a**,**b**, and **5a**–**h**^19,20^

**Scheme 2:**
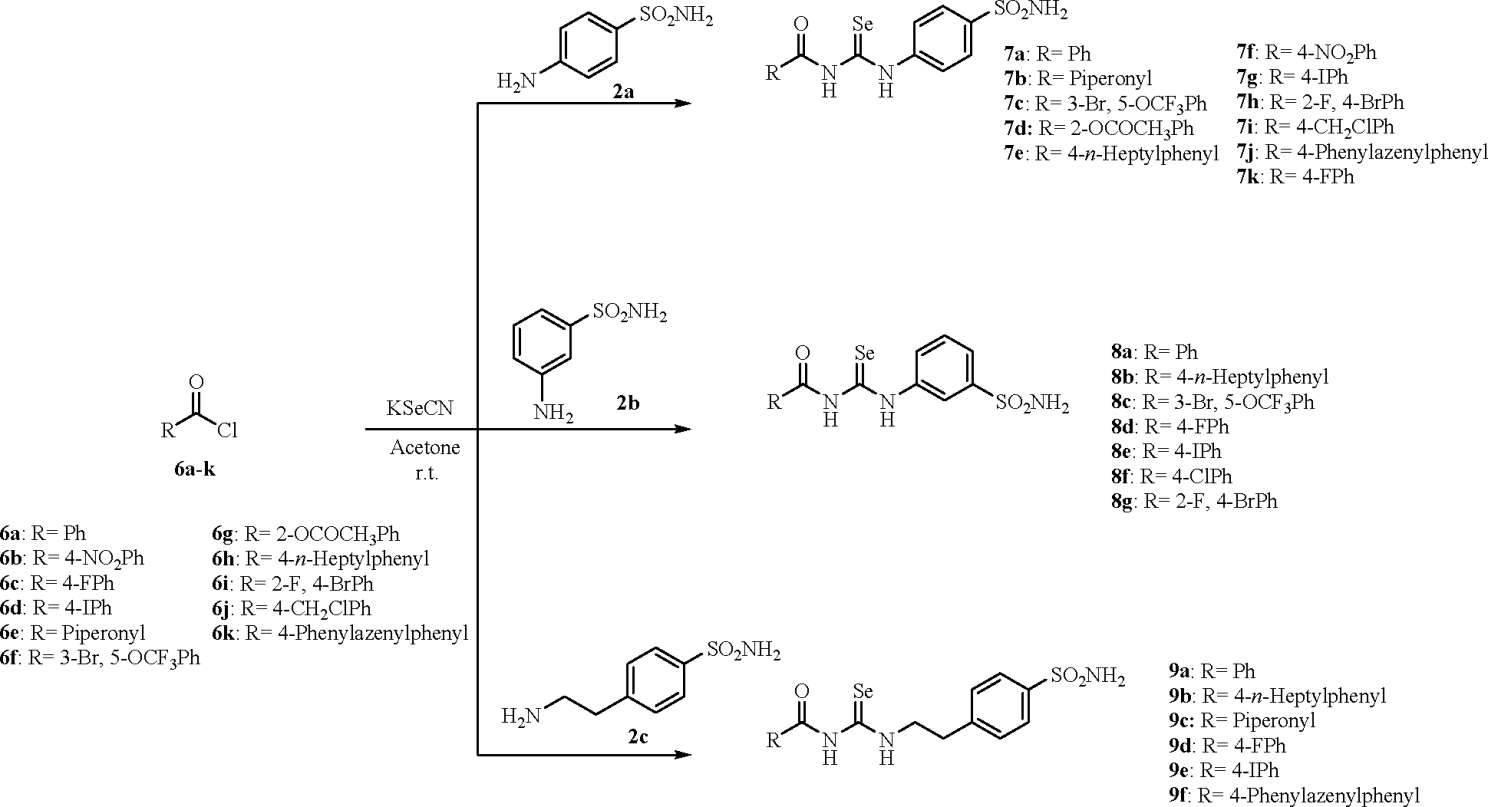
Synthesis of Acylselenoureas **7a**–**k**, **8a**–**g**, and **9a**–**f**

**Scheme 3:**
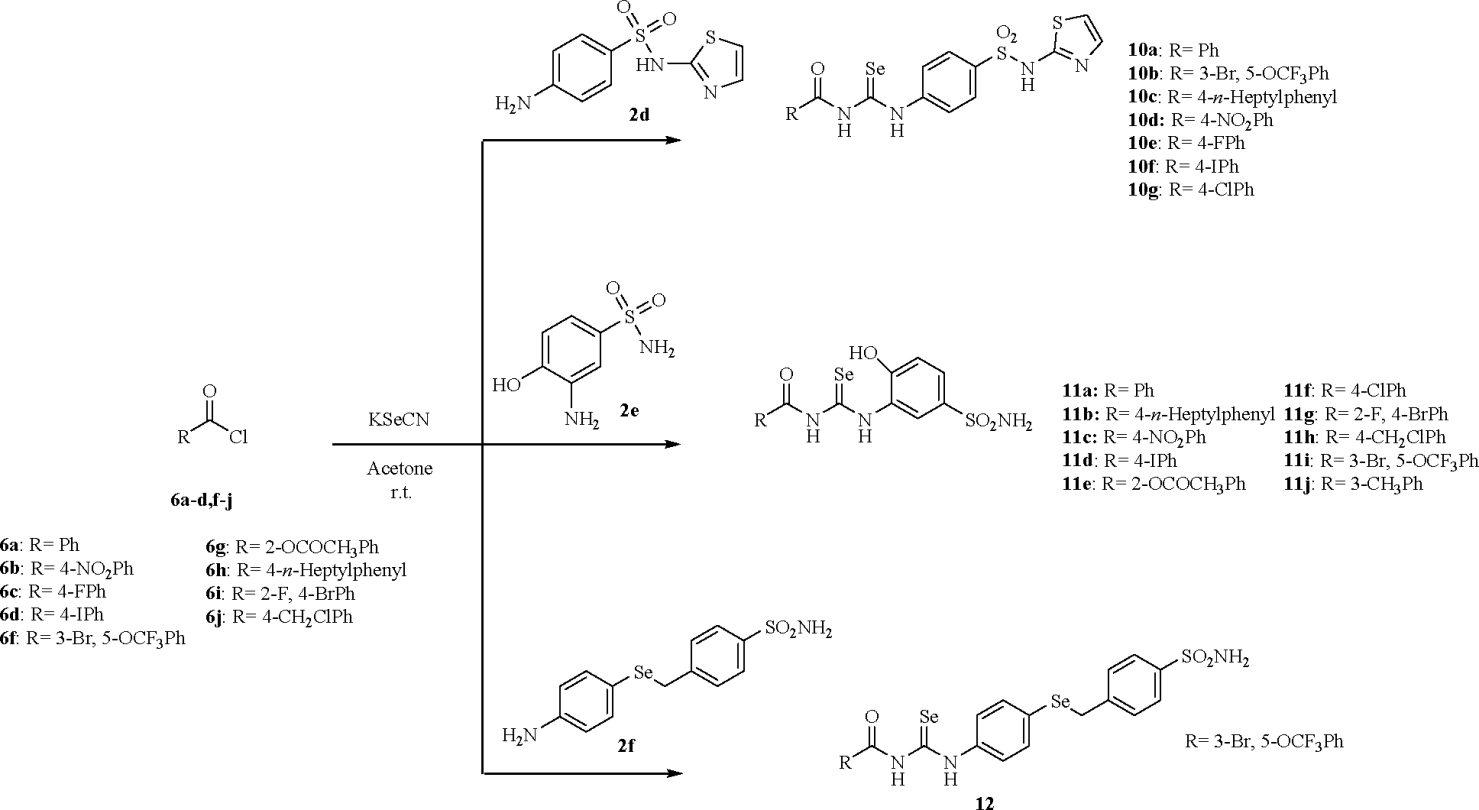
Synthesis of Acylselenoureas **10a**–**g**, **11a**–**j**, and **12**

Since chemically induced deselenizations on either selenoureas and acylselenoureas all rely on the high polarizability of selenium,^18,19^ we envisioned such moieties as ideal scaffolds to deliver this element when exposed to yeast metabolic transformations. Some of us reported selenoureas and acylselenoureas merged to primary/secondary aryl sulfonamides as effective inhibitors of the human and bacterial expressed CAs.^19−24^ For the purpose of this study, we further expanded the compounds’ library according to Schemes 1–3 taking into consideration that the sulfonamide moiety (i.e., primary and secondary) is also highly advantageous in reducing the lipophilic load which is too high when the selenium element is introduced into such small molecules.

The synthetic route developed for the preparation of selenoureas **3**–**5** makes use of classic coupling reactions between the isoselenocyanate **1a**–**i** with commercially available arylsulfonamides **2a**–**c** as earlier reported by some of us^19,20^ (Scheme 1).

A convenient chemical modification on Scheme 1 is operated by insertion of the acyl moiety to afford derivatives **7a**–**k**, **8a**–**g**, **9a**–**f**, **10a**–**g**, **11a**–**j**, and **12** as in Schemes 2 and 3. The procedure of Koketsu et al.^25^ was applied to commercially available acyl chlorides **6a**–**k** to generate *in situ* the corresponding acyl isoselenocyanates which in turn were trapped with primary anilines/amines **2a**–**f**.^19−23^

**Scheme 4:**
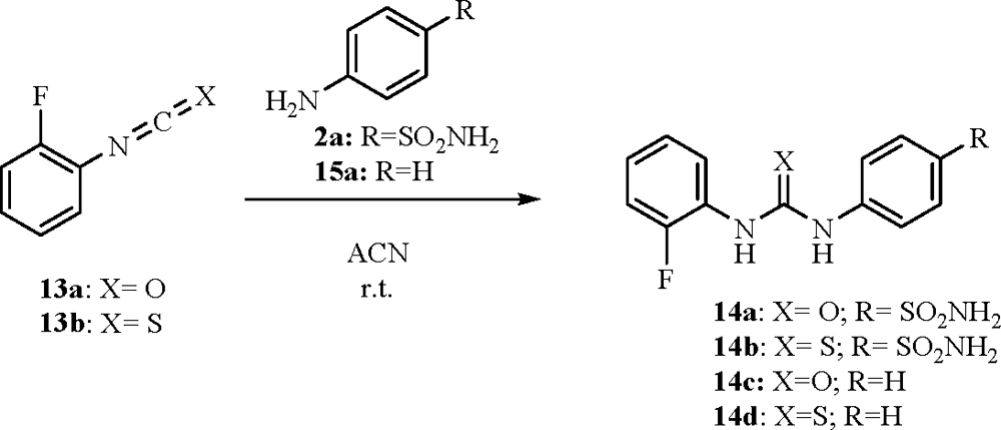
Synthesis of **14a**–**d**

**Scheme 5:**
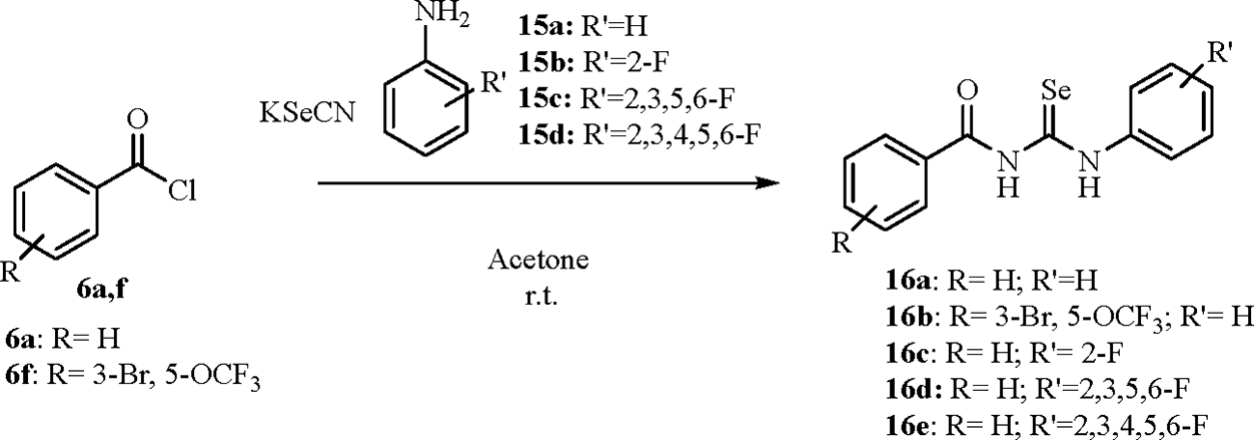
Synthesis of **16a**–**e**

In order to assess the contribution of either the selenium and the sulfonamide as inhibitor moiety of the CA of interest, we synthesized ureas and acylureas of the type in Schemes 4 and 5. In analogy to the synthetic pathway in Scheme 1, the ureas **14a**,**c** and thioureas **14b**,**d** were obtained using the corresponding 2-fluoroiso(thio)cyanates **13a**,**b**.

The method of Koketsu for the acylselenoureas was applied for the obtainment of derivatives **16a**–**e**, which are all devoid of the primary sulfonamide moiety (Scheme 5).

### Fungal Inhibitory Activity

The ability of the herein-reported compounds to reduce the growth of fungal strains of human and veterinary relevance was assessed by microdilution broth assay and calculating the minimal inhibitory concentration (MIC) after 24–48 h of incubation at 33/37 °C. Compounds **3a**, **3c**, **5b**, **7a**–**d**, **9c**, **11e**, **11i**, and **14a**,**b** were selected as representatives of the chemical moieties synthesized prior to their screen on yeasts of biomedical relevance such as *M. pachydermatis* DSMZ 6172, *C. albicans* ATCC 10231, and *C. glabrata* ATCC 90030 (DSMZ 11226) strains. The clinical used drugs Ketoconazole, SeS_2_, and Amphotericin B were included as references (Table 1).

**Table 1:** Minimal Inhibitory Concentration (MIC) of Compounds **3a**, **3c**, **5b**, **7a**–**d**, **9c**, **11e**, **11i**, and **14a**,**b** on *M. pachydermatis*, *C. albicans*, and *C. glabrata*^a^

MICs (μg/mL)^b^			
compounds	*M. pachydermatis* DSMZ 6172	*C. albicans* ATCC 11006	*C. glabrata* DSMZ 11226
**3a**	0.5 ± 0	64 ± 0	10.7 ± 0
**3c**	64 ± 0	341.3 ± 0	64 ± 0
**5b**	0.25 ± 0	106.7 ± 0	3.3 ± 0
**7a**	0.5 ± 0	128 ± 0	32 ± 0
**7b**	0.5 ± 0	128 ± 0	16 ± 0
**7c**	0.5 ± 0	64 ± 0	8 ± 0
**7d**	3.33 ± 1.15	128 ± 0	64 ± 0
**9c**	1.0 ± 0	>256	>256
**11e**	0.5 ± 0	256 ± 0	256 ± 0
**11i**	0.5 ± 0	64 ± 0	32 ± 0
**14a**	>256	>256	>256
**14b**	>256	>256	>256
Ketoconazole	0.03 ± 0	0.05 ± 0.02	96 ± 37.0
SeS_2_	0.16 ± 0.06	2.0 ± 0	1.0 ± 0
Amphotericin B	0.43 ± 0.12	0.24 ± 01	0.98 ± 0

a Ketoconazole, SeS_2_, and Amphotericin B were used as reference drugs.

b Mean ± SD from at least three different determinations.

Overall, data in Table 1 indicate that all seleno-containing scaffolds **3a**, **3c**, **5b**, **7a**–**d**, **9c**, **11e**, and **11i** were endowed with appreciable antifungal activities, which were suppressed when the chalcogen element was replaced with either cognate isosteric elements sulfur and oxygen (i.e., **14a** and **14b** with MICs > 256 μg/mL). As a side note, the piperonyl acylselenoyl derivative **9c** possessed antifungal activity only against *M. pachydermatis* (MIC of 1.0 μg/mL). Overall data indicated that the selenium-containing derivatives preferentially showed growth inhibition for the zoophilic *Malassezia* over the human commensal *Candida* species. Compound **3c** was the only exception as equally potent (MIC of 64 μg/mL) on both *M. pachydermatis* and *C. glabrata* (Table 1). Compounds **3a**, **5b**, **7a**–**c**, **11e**, and **11i** showed MIC values comparable to the reference drugs Amphotericin B (0.43 μg/mL) and thus slightly superior to either the Ketoconazole and SeS_2_ (i.e., MIC of 0.03 and 0.16 μg/mL, respectively).

Based on such results, we further extended the antifungal investigation of our compounds’ library against *M. globosa* and *M. furfur*, which are distributed mainly on human skin, causing dermatitis and dandruff when commensal conditions are altered (Table 2).

**Table 2:** Minimal Inhibitory Concentration (MIC) of Compounds **3a**–**e**, **4a**,**b**, **5a**–**h**, **7a**–**k**, **8a**–**g**, **9a**–**f**, **10a**–**g**, **11a**–**j**, **12**, **14a**–**d**, and **16a**–**d** on *M. pachydermatis*, *M. globosa*, and *M. furfur* Strains Compared to the Clinically Used Drugs Ketoconazole, SeS_2_, and Amphotericin B

MIC (μg/mL)^a^			
compounds	*M. pachydermatis* DSMZ 6172	*M. globosa* ATCC MYA 4612	ATCC *M. furfur* 14521
**3a**	0.5 ± 0	4.3 ± 1.97	0.5 ± 0
**3c**	64 ± 0	>256 ± 0	16 ± 0
**3d**	6 ± 3.46	>256 ± 0	213.3 ± 73.9
**3e**	1 ± 0.87	256 ± 0	5.3 ± 2.31
**4a**	4 ± 0	256 ± 0	106.7 ± 36.95
**5b**	0.25 ± 0	9 ± 5.9	1 ± 0
**5e**	0.5 ± 0	256 ± 0	2.3 ± 1.53
**5f**	0.5 ± 0	256 ± 0	1.3 ± 0.58
**5g**	1 ± 0	85.3 ± 36.95	1.7 ± 0.58
**5h**	0.5 ± 0	64 ± 0	1.7 ± 0.58
**7a**	0.5 ± 0	14.7 ± 3.26	0.85 ± 0.36
**7b**	0.5 ± 0	16 ± 8.76	0.5 ± 0
**7c**	0.5 ± 0	128 ± 0	4 ± 0
**7d**	3.33 ± 1.15	256 ± 0	2.7 ± 1.15
**7e**	0.5 ± 0	>256 ± 0	10.7 ± 4.62
**7f**	2.2 ± 1.75	64 ± 0	42.7 ± 18.47
**7g**	0.5 ± 0	256 ± 0	6 ± 3.46
**7h**	0.5 ± 0	64 ± 0	3.3 ± 1.15
**7i**	6.7 ± 2.31	26,7 ± 9.2	16 ± 0
**7j**	2 ± 0	24 ± 13.9	21.3 ± 9.24
**7k**	0.5 ± 0	256 ± 0	5.3 ± 2.31
**8a**	2 ± 0	128 ± 0	21.3 ± 9.24
**8b**	0.8 ± 0.29	213.3 ± 73.9	6.7 ± 2.31
**8c**	2 ± 0	256 ± 0	9.3 ± 6.11
**8d**	2 ± 0	256 ± 0	256 ± 0
**8e**	8 ± 0	>256 ± 0	6.7 ± 2.31
**8f**	16 ± 0	128 ± 0	64 ± 0
**8g**	13.3 ± 4.6	128 ± 0	8 ± 0
**9a**	1 ± 0	32 ± 0	4 ± 0
**9b**	1.7 ± 0.58	256 ± 0	1.7 ± 0.58
**9c**	1 ± 0	37.3 ± 13.06	2 ± 1.37
**9d**	1 ± 0	128 ± 0	2.3 ± 2.31
**9e**	1 ± 0	32 ± 0	3.3 ± 1.15
**9f**	3.33 ± 1.15	8 ± 0	2.7 ± 1.15
**10a**	1 ± 0	128 ± 0	8 ± 0
**10b**	16 ± 0	341.3 ± 147.8	426.7 ± 147.8
**10c**	0.8 ± 0.29	>256 ± 0	4 ± 0
**10d**	1.8 ± 1.89	26.7 ± 9.2	85.3 ± 36.95
**10e**	1 ± 0	128 ± 0	2.3 ± 2.31
**10f**	2 ± 0	>256 ± 0	5.3 ± 2.31
**10g**	1.3 ± 0.58	>256 ± 0	64 ± 0
**11a**	1 ± 0	128 ± 0	2.7 ± 1.15
**11b**	8 ± 0	256 ± 0	5.3 ± 2.31
**11c**	0.5 ± 0	128 ± 0	5.3 ± 2.31
**11d**	13.3 ± 4.6	128 ± 0	10.7 ± 4.62
**11e**	0.5 ± 0	6.7 ± 2.1	0.75 ± 0.67
**11f**	0.5 ± 0	256 ± 0	4 ± 0
**11g**	3.33 ± 1.15	256 ± 0	5.3 ± 2.31
**11h**	4 ± 0	>256 ± 0	74.7 ± 48.88
**11i**	0.5 ± 0	64 ± 0	5 ± 2.45
**11j**	0.5 ± 0	128 ± 0	16 ± 8.76
**12**	6.7 ± 2.3	256 ± 0	128 ± 0
**14a**	>256	>256	>256
**14b**	>256	>256	>256
**14c**	>256	>256 ± 0	>256
**14d**	>256	>256	128 ± 0
**16a**	0.8 ± 0.29	29.3 ± 18.7	0.8 ± 0.6
**16b**	1 ± 0	341.3 ± 147.8	10.7 ± 4.62
**16c**	1 ± 0	64 ± 0	2 ± 0
**16d**	1 ± 0	>256	2.7 ± 1.15
**16e**	1.8 ± 1.9	85.3 ± 36.9	4 ± 0
Ketoconazole	0.03 ± 0	0.25 ± 0	0.25 ± 0
SeS_2_	0.16 ± 0.06	0.5 ± 0	0.03 ± 0.01
Amphotericin B	0.43 ± 0.12	125 ± 0	1.95 ± 0

a Mean ± SD from at least three different determinations.

The selenoureido **3a** showed the highest antifungal activity (MIC 0.5 μg/mL) against *M. pachydermatis* compared to its substituted derivatives **3c**–**e**. The regioisomeric metanilamide **4a** reported an 8-fold decrease in antifungal growth potency (MIC 4.0 μg/mL). Interestingly, **3e** showed greater selectivity than **3a** for *M. pachydermatis* and *furfur* strains over *M. globosa*. Molecular elongation as in compounds **5b**–**h** did not affect the high efficacy and preferential selectivity for *M. pachydermatis*. Noteworthy, the 2-fluorophenyl derivative **5b** resulted particularly effective against the *M. pachydermatis* strain, showing a MIC value of 0.25 μg/mL, thus 1.7-fold more potent when compared to Amphotericin B (MIC of 0.43 μg/mL) and up to 256-fold than its shorter congener **3c** (MIC of 64 μg/mL). Among the aromatic acyl selenoureido containing derivatives (i.e., series **7**–**12** and **16**) the antifungal activity was retained. Overall, such compounds had antifungal growth efficacies and selectivity against *M. pachydermatis* over the other strains considered in this study. Exceptions were encountered, and accounted for **3a**, **7b**, **7d**, **8e**, **8g**, **9b**, **9f**, **11b**, **11d**, **11e**, **11g**, **16a**, and **16e**, which showed significative matching MIC values for either *M. pachydermatis* or *M. furfur.* Only compounds **3c** caused antifungal effects preferentially on *M. furfur* (MIC of 0.5 μg/mL) although with less efficacy when compared to the reference drugs (Table 2).

The *M. globosa* strain was the least affected by the compounds tested as very high MIC values were obtained. Among the series, the derivatives **3a** and **11e** resulted the most effective, with MIC values in the low μg/mL range (i.e., MICs of 4.3 and 6.7 μg/mL for **3a** and **11e**, respectively).

The significance of the selenium moiety to endow the compounds here reported with antifungal effects is demonstrated by the ineffectiveness of **14a**–**d** on *Malassezia* strains reported in this study (Table 2). Besides the preeminent selectivity of the seleno-containing compounds for *M. pachydermatis*, it is worth noting that the magnitude of the antifungal activities was comparable to the clinically used drugs Ketoconazole, SeS_2_, and Amphotericin B (Table 2).

### *In Vitro* Cytotoxicity Assay

**Figure 1:**
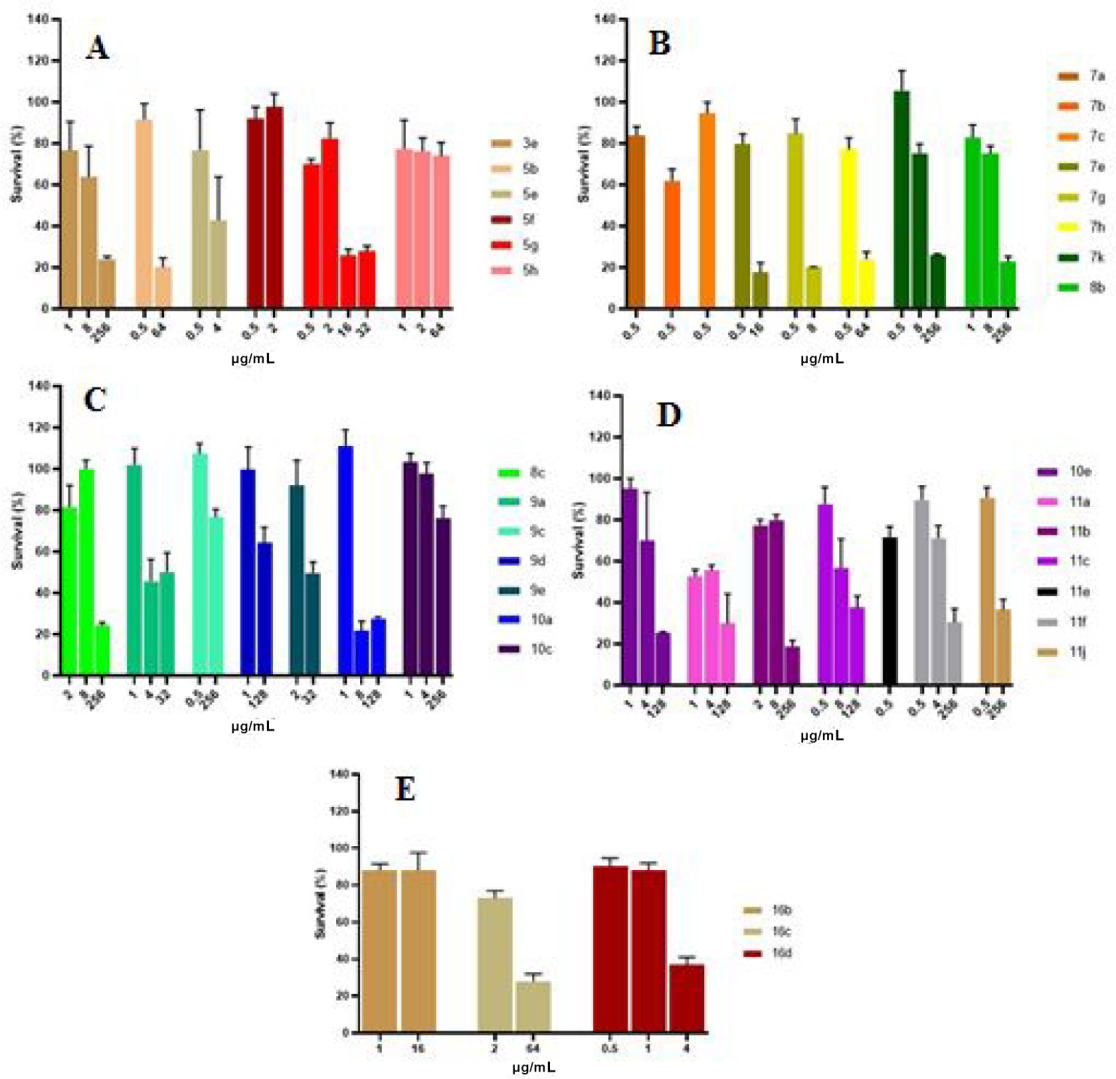
(A–E) Cytotoxicity of selected compounds tested on MDBK cells, indicated as cell viability (%) by MTT assay.

Compounds endowed with the best performing MIC values on *M. pachydermatis* were investigated for the cytotoxic effects on bovine kidney cells (MDBK) by the MTT assay (Figure 1).

Overall, the tested compounds showed dose-dependent cytotoxic effects with appreciable differences. Data in Figure 1A explained that the selenoureido-containing derivatives **3e**, **5b**, **5e**, and **5g** were toxic at concentrations of 256, 64, 4, and 16 μg/mL, respectively. Superior safety profiles were observed for **5f** and **5h**, which showed no appreciable reductions in cell viability at the maximum concentrations used for the assay.

The acyl selenoureido **7a**–**c** showed marginal safety profiles as the decrease of cell viability was already remarkable when tested at the corresponding MIC values (Figure 1B). As for the remaining acyl selenoureido compounds bearing either the sulfanilamide (i.e., **7e**,**g**,**h**,**k**) or metanilamide (i.e., **8b**,**c**) moieties, dose-dependent cytotoxicity was reported (Figure 1B,C) with concentration values up to 512-fold their MICs (cfr. Table 2 and Figure 1B,C). As for the elongated acyl-selenoureido derivatives **9a**, **9c**–**e** high cytotoxic values were obtained for **9c** and **9d**, whereas the remaining compounds were toxic at lower concentrations (Figure 1C). Drastic reduction of the cell viability was observed for the secondary sulfonamide containing compound **10a** at 8 μg/mL with no appreciable effects when tested at higher doses (Figure 1C). Interestingly, introducing a fluorine atom within **10a** to afford **10e** resulted in a restrained dose-dependent cytotoxic curve with no significant changes in cell viability when maximal doses were reached (i.e., 128 μg/mL). Conversely, a linear alkyl chain as in **10c** greatly influenced the compounds’ toxicity profile, which did not induce a notable cell death up to 256 μg/mL (Figure 1C).

Among the phenolic-containing derivatives, the acetyl **11e** was the least tolerated as a slight increase of the administered concentration above the MIC value (i.e., 0.5 μg/mL) drastically reduced the viability of MDBK cells (Figure 1D). The unsubstituted derivative **11a** and the *n*-heptyl bearing **11b** showed similar trends when tested on MDBK cells; the latter is better tolerated at twice the concentration when compared to its cognate precursor (Figure 1D). Similarly, **11f** resulted in 2-fold less cytotoxic than **11c**, which was comparable to **11j** (Figure 1D).

The acyl-seleno derivatives **16b**–**d** lacking the hydrophilic sulfonamide moiety were much more cytotoxic than the entire compound series presented in this study (Figure 1E). The increased lipophilic load of these molecules can reasonably be responsible for such an effect as they are considered keener to interact with lipophilic biomembranes.

**Figure 2:**
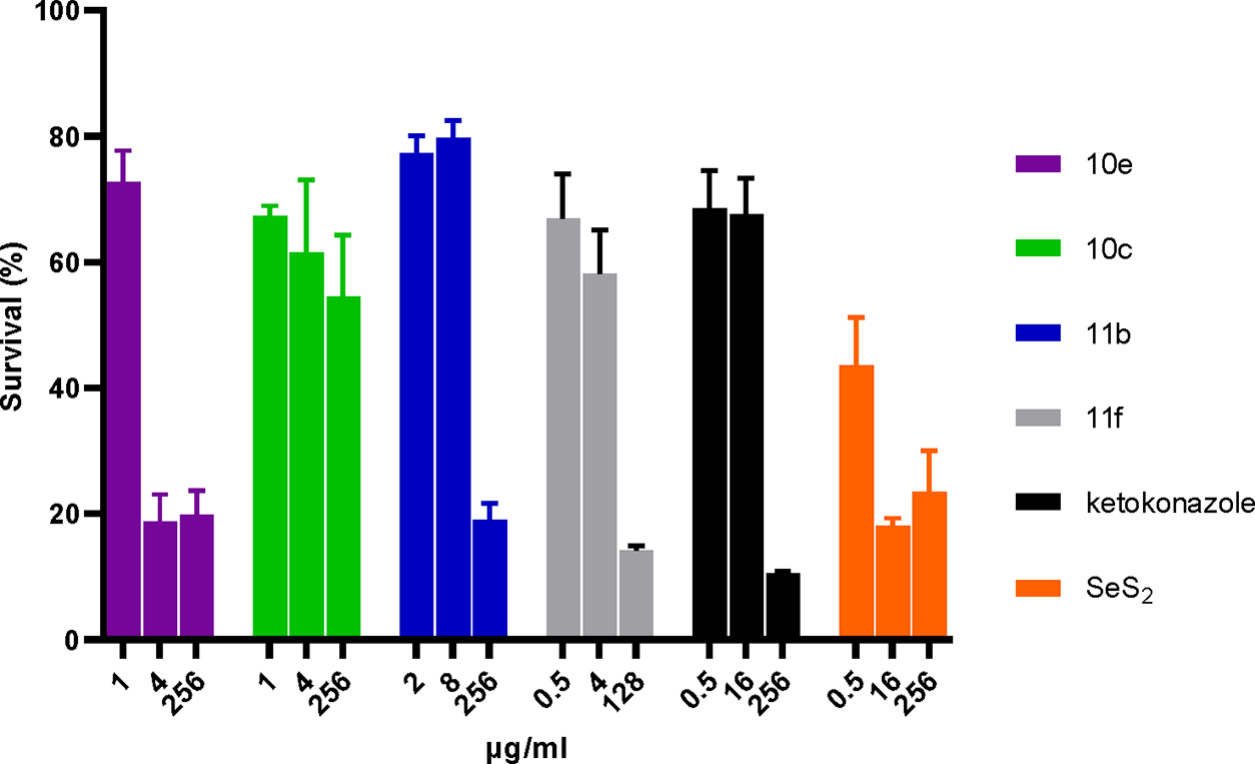
Cytotoxicity of **10c**, **10e**, **11b**, and **11f** on HACat cells indicated as cell viability (%) by MTT assay.

Since the *Malassezia* spp. strains are mainly found on the outer skin layers of animal hosts, including humans, we assessed the cytotoxic effects of selected compounds against human keratinocytes compared to the clinically approved antifungal drugs ketoconazole and SeS_2_. The best performing acyl-seleno ureido derivatives in terms of low cytotoxicity on Human Keratinocytes cells (HaCat) were **10c**, **10e**, **11b**, and **11f**. The results are reported in Figure 2.

**Figure 3:**
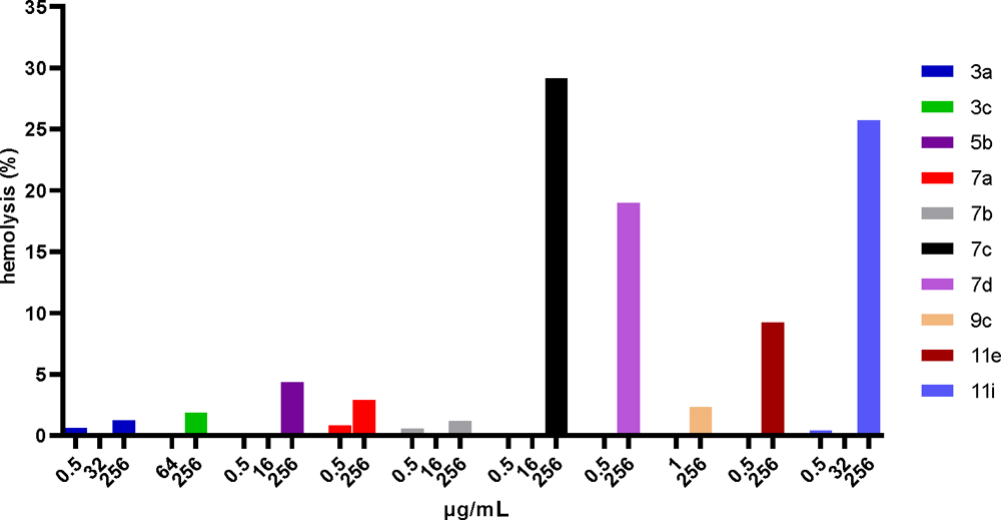
Hemolysis assay of compounds **3a**, **3c**, **5b**, **7a**–**d**, **9c**, **11e**, and **11i** on defibrinated sheep blood. Reported percentages are referred to the hemolytic effect scale, where 0% = all intact cells and 100% = hemolytic effect extended to all cells.

Interestingly, SeS_2_ showed high cytotoxic effects at the lowest concentration (i.e., 0.5 μg/mL), followed by the halogen-containing derivatives **10e** and **11f**, which substantially suppressed cell survival at lower concentrations than the clinically used drug Ketoconazole (Figure 2). Among the *n*-heptyl derivatives, compound **11b** was safer for each tested dose than ketoconazole (Figure 2). More importantly, the derivative **10c** resulted in far less cytotoxic among all the selected compounds and with respect to the clinical drug ketoconazole up to the maximum concentration (i.e., 256 μg/mL), thus supporting this compound as a good candidate for advanced testing. Finally, the hemolytic activity on defibrinated sheep blood for selected compounds (**3a**, **3c**, **7a**–**d**, **9c**, **11e**, and **11i**) was investigated, and the results are reported in Figure 3.

Compounds **7c**, **7d**, **11e**, and **11i** caused hemolysis over the 10% only when tested at maximum dosage (i.e., 256 μg/mL). As for the remaining compounds very low hemolytic events (<5%) were observed when tested at the same concentration.

### *In Vitro* Carbonic Anhydrase Activity

The presence of the sulfonamide moiety (i.e., either primary/secondary) within our scaffolds prompted us to investigate whether the compounds listed in this study were endowed with modulation features against the enzymatic metalloenzyme carbonic anhydrases (CAs, EC 4.2.1.1) expressed in *Malassezia spp*. of interest such as *M. pachydermatis* (MpaCA), *M. globosa* (MgCA), and *M. restricta* (MreCA). The inhibition properties of **3a**–**e**, **4a**,**b**, **5a**–**h**, **7a**–**k**, **8a**–**g**, **9a**–**f**, **10a**–**g**, **11a**–**j**, **12**, **14a**–**d**, and **16a**–**d** on the fungal CAs were all assessed *in vitro* by means of the stopped-flow technique and compared to the standard CAI of the sulfonamide type acetazolamide (**AAZ**). The obtained activities in Table 3 were all reported as *K*_I_ values.

**Table 3:** Inhibition Data of **3a**–**e**, **4a**,**b**, **5a**–**h**, **7a**–**k**, **8a**–**g**, **9a**–**f**, **10a**–**g**, **11a**–**j**, **12**, **14a**–**d**, and **16a**–**d** and the Standard Sulfonamide Inhibitor Acetazolamide (**AAZ**) against MpaCA, MgCA, and MreCA and by Means of the Stopped-Flow CO_2_ Hydrase Assay^26^

*K*_I_ (nM)^a^			
compounds	MpaCA	MgCA	MreCA
**3a**	514.1	599.1	857.6
**3b**	965.2	648.5	80.7
**3c**	151.9	720.5	93.1
**3d**	616.9	803.4	87.9
**3e**	692.8	655.2	423.9
**4a**	282.5	783.1	782.4
**4b**	342.1	700.3	855.6
**5a**	793.3	654.7	763.8
**5b**	148.8	632.7	86.4
**5c**	992.3	700.8	847.0
**5d**	369.4	682.7	90.5
**5e**	518.4	812.4	85.1
**5f**	525.5	411.5	601.5
**5g**	530.6	689.4	2701
**5h**	259.7	561.5	95.1
**7a**	44770	3855	4480
**7b**	20384	4856	3746
**7c**	83470	4300	6910
**7d**	66830	5882	5749
**7e**	78230	5878	7000
**7f**	85440	9014	2250
**7g**	76110	3110	3880
**7h**	68540	763.6	5160
**7i**	67680	876.9	7750
**7j**	37030	2861	5230
**7k**	80800	8288	3800
**8a**	33710	4136	8100
**8b**	49310	2187	40.0
**8c**	86110	7651	7950
**8d**	79460	7296	4380
**8e**	77600	8741	6560
**8f**	74680	2078	7200
**8g**	65660	3481	4350
**9a**	73860	564.7	7210
**9b**	>10000	6389	3960
**9c**	55900	1775	4639
**9d**	74990	7997	3590
**9e**	83330	1568	7510
**9f**	88780	2386	2730
**10a**	42940	52780	6130
**10b**	85350	9260	2800
**10c**	91880	60570	5670
**10d**	92800	8770	4600
**10e**	80300	8900	4900
**10f**	74260	10200	8200
**10g**	66670	36060	6750
**11a**	13420	4095	1160
**11b**	77970	4931	8250
**11c**	91200	5015	3500
**11d**	7499	2932	6180
**11e**	33850	4729	4290
**11f**	54170	5131	5230
**11g**	70880	6006	5780
**11h**	76620	6487	6830
**11i**	12745	6251	5832
**11j**	14837	5644	5677
**12**	71560	1764	6130
**14a**	844.8	6744	106.8
**14b**	488.1	5492	143.3
**14c**	>10000	>10000	>10000
**14d**	>10000	>10000	>10000
**16a**	>10000	>10000	>10000
**16b**	>10000	>10000	>10000
**16c**	>10000	>10000	>10000
**16d**	>10000	>10000	>10000
**AAZ**	620.0	74000	100.0

a Mean from 3 different assays, by a stopped flow technique (errors were in the range of ± 5–10% of the reported values).

As expected, all compounds devoid of the sulfonamide moiety (either primary or secondary) **14c**, **14d**, and **16a**–**d** resulted in ineffective inhibitors of the CA panel considered having *K*_I_ values >10000 nM, whereas the remaining derivatives showed inhibition potencies comprised in the micromolar range (Table 3).

Specifically, the following structure–activity relationships (SARs) can be drawn:

(i) As for the recently cloned MpaCA isozyme, the introduction of the fluoro within **3a** to afford **3b** spoiled the inhibition potency up to 1.9-fold (*K*_I_s of 514.1 and 965.2 nM, respectively). Interestingly, a significant regioisomeric effect occurred when the halogen was switched to 2-position (*K*_I_ of **3c** 151.9 nM). Conversely, either the bulky trifluoromethyl moiety (i.e., **3d**) or the chloro (i.e., **3e**) resulted in detrimental MpaCA inhibition (*K*_I_s of 616.9 and 692.8 nM). A sulfonamide-dependent regioisomeric effect was particularly relevant for **4a** and **4b**, which resulted in 1.8- and 2.8-fold less potent when compared to the cognate para-substituted derivatives **3a** and **3b**, respectively (*K*_I_s of 282.5 and 342.1 nM). Either elongation of the CAI warhead (i.e., **5a**–**c** and **5e**) along with the introduction of various chemical moieties (i.e., **5d**, **5f**–**h**) did not appreciably affect the MpaCA inhibition potencies when compared to the shortest analogues previously discussed (Table 3).
  Introduction of the arylacyl moiety as in the **7a**–**k**, **8a**–**g**, **9a**–**f**, **10a**–**g**, **11a**–**j**, and **12** switched the *K*_I_ inhibition values against the MpaCA toward medium-high micromolar ranges. Specifically, among the sulfanilamide derivatives **7a**–**k** elaboration of the phenyl ring in **7a** to the benzo[*d*]-[1,3]dioxole (i.e., piperonyl) to afford **7b**, enhanced the inhibition potency up to 2.2-fold (*K*_I_s of 44.8 and 20.4 μM for **7a** and **7b**, respectively). Interestingly, the 4-azophenyl substitution in **7a** (i.e., **7j**) also reduced the *K*_I_ value, although very slightly (i.e., 1.2-fold, see Table 3). As for the remaining compounds within the **7a**–**k** series, variable substitutions at the tail-end did not improve the inhibition potency nor determine significative *K*_I_ changes (Table 3). In analogy to the shortest derivative previously discussed also in this case regioisomeric effects were reported when the primary sulfonamide moiety was shifted. For instance, the metanilamide containing **8a** resulted in 2.3-fold more potent than its related sulfanilamide regioisomer **7a** (*K*_I_s of 33.7 and 44.8 μM for **8a** and **7a**, respectively). Although with a lower magnitude (i.e., 1.6-fold), the same trend was reported for **8b** and **7e** (Table 3). No relevant differences were reported for the remaining compounds within the **8a**–**g** series, which resulted in all medium-range micromolar MpaCA inhibitors. Similar considerations are valid for the elongated derivatives **9a**–**f**, which reported an almost flat kinetic profile compared to their shortest counterparts within the **7a**–**k** and **8a**–**g** series (Table 3). It is worth noting that elaboration of the CAI warhead with an additional phenolic moiety had circumstantial effects on the kinetics, as clearly reported in Table 3 when compounds **11a**–**j** were compared to the cognate series **8a**–**g** (Table 3). Specifically, the chloro derivative **11f** was 1.4-fold more effective than **8f** (*K*_I_s of 54.2 and 74.7 μM for **11f** and **8f**, respectively), followed by **11a**/**8a** (i.e., 2.5-fold with *K*_I_s of 13.4 and 33.7 μM for **11a** and **8a**, respectively), **11i**/**8c** (i.e., 6.8-fold with *K*_I_s of 12.8 and 86.1 μM for **11i** and **8c**, respectively), and **11d**/**8e** (i.e., 10.3-fold with *K*_I_s of 7.5 and 77.6 μM for **11d** and **8e**, respectively). As for the remaining compounds, it is worth noting the 3-methyl derivative **11j**, which showed a *K*_I_ value of 14.8 μM (Table 3). Molecular elongation of **7c** by means of insertion of the phenylselenylmethyl spacer to afford compound **12** slightly enhanced the inhibition potency up to 1.2-fold (*K*_I_s of 83.5 and 71.6 μM for **7c** and **12**, respectively). The secondary sulfonamide derivatives **10a**–**g** were all high micromolar range MpaCA inhibitors with very marginal differences from each other. The most potent compound within the series was the **10a**, which showed a close matching *K*_I_ value with its primary sulfonamide containing cognate **7a** (i.e., *K*_I_s of 42.9 and 44.8 μM for **10a** and **7a**, respectively).
(ii) As for the MgCA isozyme, all the selenoureido derivatives **3a**–**e** resulted high nanomolar inhibitors with *K*_I_ values spanning between 599.1 and 803.4 nM, and thus, no significative differences were observed among the series. Slight regioisomeric effects (i.e., up to 1.3-fold) were observed when the primary sulfonamide moiety in **3a** and **3b** was moved to the meta position to afford compounds **4a** and **b**, respectively (Table 3). The elongation strategy did not prove successful in obtaining more effective inhibitors of the MpaCA isoforms as clearly reported from the data in Table 3 referred to **5a**–**h**. Although they showed *K*_I_ values lined with the shortest derivatives **3a**–**e** and **4a**,**b** SARs were better defined. The most important entries account for the 2-methoxy derivative **5f**, which resulted 1.6-fold more potent than the unsubstituted cognate **5a** (i.e., 654.7 and 411.5 nM for **5a** and **5f**, respectively), followed by the 2-naphthyl **5h** (i.e., *K*_I_ of 561.5 nM) and the 2-fluoro **5b** (i.e., *K*_I_ of 632.7 nM), whereas all the remaining compounds in the series resulted less effective than **5a**. Also on the MgCA, the acyl moiety (i.e., series **7a**–**k**, **8a**–**g**, **9a**–**f**, **10a**–**g**, **11a**–**j**, and **12**) determined reduction of the ligand inhibitory activities although the *K*_I_ values resulted an order of magnitude lower when compared to those registered for the MpaCA isoform (Table 3). All the substitutions within **7a** to afford **7b**–**f** and **7j**–**k** spoiled the inhibition values up to 2.3-fold (i.e., cfr. **7f** over **7a**). Enhancement of the compounds potency was obtained when the 4-iodo phenyl moiety was placed, as in **7g** (i.e., *K*_I_ of 3.1 μM), and it was additionally reinforced by means of introduction at the same position of the chloromethylene moiety (i.e., *K*_I_ of 0.88 μM for **7i**) followed by multiple halogen substitution as in **7h** (i.e., *K*_I_ of 0.76 μM). Relevant impacts on kinetics were observed when compounds **8a**–**g** within the metanilamide series was compared to their corresponding sulfanilamide regioisomers **7a**–**k** (Table 3). Slight increases (i.e., 1.1-fold) of the *K*_I_ values were reported for the unsubstituted derivative **8a** over **7a**, whereas more consistent effects were observed for **8***c***/7c** (1.8-fold), **8e**/**7g** (2.8-fold), and **8g**/**7h** (4.6-fold). Only two metanilamide regioisomers (i.e., **8b** and **8d**) showed enhancement of the inhibition potency on MgCA when compared to their sulfanilamide counterparts (i.e., **7e** and **7k**). *K*_I_ value ratios in Table 3 accounted for the metanilamide regioisomers being 2.7- and 1.1-fold more potent. Interesting results were also obtained when the elongation strategy was applied. For instance, the phenyl substituted derivative **9a** resulted particularly effective as inhibitor of the MgCA being 6.8-fold more potent when compared to its shorter cognate **7a** (i.e., *K*_I_s of 564.7 and 3855 nM for **9a** and **7a**, respectively). Additional elaborations as in **9b**–**f** were not beneficial for the *K*_I_ values which all resulted comprised between 1568 and 6389 nM (Table 3). Noteworthy is the halogen effect on *in vitro* kinetics as shown by **9d** and **9e**. The bulky electron rich iodo derivative **9e** resulted 5.1-fold more effective inhibitor for the MgCA when compared to the fluoro containing **9d** (i.e., *K*_I_s of 1568 and 7997 nM for **9e** and **9d**, respectively). The kinetic data relative to the phenolic containing substituents reported values comprised in the low micromolar range. The introduction within **11a** of the iodo moiety to afford the derivative **11d** was the only entry which determined enhancement of the inhibition potency (i.e., up to 1.4-fold), whereas all the remaining compounds induced significative reduction (Table 3). Of interest is the *in vitro* activity for the elongated compound **12** which reported a *K*_I_ value of 1.76 μM, thus being among the most effective acylselenoureido derivatives on MgCA. Variable kinetic trend associated with increase of the associated values was obtained when the phenolic moiety was considered (Table 3, entries **11a**–**j**). Among the series, the iodo containing **11d** was the most effective inhibitor for the MgCA isozyme (i.e., *K*_I_ of 2.9 μM). As expected, the secondary sulfonamide derivatives **10a**–**g** resulted far less efficient inhibitors of the MgCA isoform when compared to their cognates **7a**–**k** (Table 3). Quite unexpectedly, the nitro containing **10d** showed a *K*_I_ value close matching with its structurally analogue **7f** bearing the primary sulfonamide group (*K*_I_s of 8770 and 9014 nM for **10d** and **7f**, respectively).
(iii) As for the MreCA, effective inhibition values were obtained for **3b**–**d**, which reported *K*_I_ values of 80.7, 93.1, and 87.9 nM, whereas the unsubstituted **3a** and the 2-chloro **3e** were effective only at high nanomolar concentrations (i.e., *K*_I_s of 857.6 and 423.9 nM for **3a** and **3e**, respectively). A slight regioisomeric effect was evident for the meta unsubstituted derivative **4a** when compared to its cognate **3a** (i.e., *K*_I_ of 857.6 and 782.4 nM for **3a** and **4a**, respectively). The 4-fluoro derivative **4b** resulted far less effective inhibitor (i.e., 10.6-fold) of the MreCA when compared to its regioisomer **3b** (*K*_I_s of 855.6 and 80.7 nM for **4b** and **3b**, respectively). Elongation of the CAI warhead as in **5a**–**h** series heavily affected the *in vitro* kinetics. As reported in Table 3, the trifluoromethyl moiety as in derivative **5c** spoiled the inhibition potency against MreCA of 9.6-fold when compared to the shortest analogue **3d**. On the contrary the insertion of the chloro group at 2 position as in **5e** resulted beneficial for the *in vitro* inhibition as it resulted 5.0-fold more potent than its cognate **3e** (*K*_I_s of 85.1 and 423.9 nM for **5e** and **3e**, respectively). Narrow differences (i.e., up to 1.1-fold) were observed for the unsubstituted derivatives **5a** and **5b** over their shorter analogues **3a** and **3c**, respectively (Table 3). Change of the halogen moiety in **5e** with the methoxy group instead (i.e., **5f**) greatly enhanced the *K*_I_ value up to 601.5 nM. Noteworthy it is the remarkable halogen effect placed at 4-position on the *in vitro* kinetics as shown for **5d** and **5g**. The *in vitro* data showed the bulky iodo derivative being 29.8-fold less potent than the bromo (i.e., *K*_I_s of 2701 and 90.5 nM for **5g** and **5d**, respectively). It is also of interest that the 2-naphthyl derivative **5h** reported close matching inhibition values to **5d** (*K*_I_s of 95.1 and 90.5 nM for **5h** and **5d**, respectively). Again, significant increase of the MreCA associated *K*_I_s were obtained when the acyl moiety was inserted within the organic scaffolds (i.e., as in **7**–**12** series Table 3). Relevant SARs accounted for a 2.0-fold potency increase when the nitro moiety was placed at 4 position within **7a** (compare *K*_I_s of 4.48 and 2.25 μM for **7a** and **7f**, respectively). Milder *K*_I_ value reductions were obtained when the iodo and the fluoro halogens were inserted at position 4 instead (*K*_I_s of 3.88, 3.80, and 4.48 μM for **7g**, **7k**, and **7a**, respectively). As for the remaining compounds within the **7a**–**k** series (i.e., **7c**–**e** and **7h**–**j**), they all resulted less effective inhibitors of the MreCA isozyme when compared to the unsubstituted cognate **7a** with no significant SARs to be reported (Table 3). Clear regioisomeric effects associated with loss of the inhibition potency on the metanilamide series were observed for **7a**/**8a** (*K*_I_s of 4.48 and 8.10 μM for **7a** and **8a**, respectively), **7c**/**8c** (*K*_I_ of 6.91 and 7.95 μM for **7c** and **8c**, respectively), **7g**/**8e** (*K*_I_ of 3.88 and 6.56 μM for **7g** and **8e**, respectively), and **7k**/**8d** (*K*_I_ of 3.80 and 4.38 μM for **7k** and **8d**, respectively). Conversely in **7e**/**8b** and **7h**/**8g** enhancement of the inhibition potency for the metanilamide derivative was observed, with associated *K*_I_ values increases of 175- and 1.2-fold, respectively (Table 3). In particular, the derivative **8b** resulted the most potent MreCA inhibitor among all the entire compound series considered in this study having a *K*_I_ of 40 nM. Elongation of the CAI warhead in **7a** to afford **9a** spoiled the MreCA inhibition potency of 1.6-fold (i.e., KIs of 4.48 and 7.21 μM for **7a** and **9a**, respectively), whereas all the remaining compounds **9b**–**f** showed enhanced inhibition against such an isoform when compared to their shorter cognate precursors (Table 3). Specifically, the diazocontaining derivative **9f** resulted the most potent MreCA inhibitor among the elongated series being ffective at 2.7 μM concentration (Table 3). Interesting effects on SARs were obtained within the phenoxymetanilamide series **11a**–**j**, with the unsubstituted progenitor **11a** being the most effective MreCA inhibitor (i.e., *K*_I_ value of 1.16 μM). The introduction of the linear *n*-heptyl chain as in **11b** was highly detrimental for the MreCA inhibition potency (*K*_I_ of 8.2 μM), whereas all the remaining compounds resulted more effective inhibitors with *K*_I_ values comprised between 6.8 and 3.5 μM for **11h** and **11c**, respectively. A slight halogen effect on kinetics was observed for the **11d** and **11f** with the chloro derivative **11f** being 1.2-fold more effective when compared to the iodo **11d** (i.e., *K*_I_s of 6.2 and 5.2 μM for **11d** and **11f**, respectively). As for the remaining derivatives within the series, no relevant SARs are to be reported (Table 3). Unexpectedly, the elongated compound **12** and the secondary sulfonamide derivative **10a** reported matching *K*_I_ values of 6.1 μM (Table 3). Among the **10a**–**h** series, the polysubstituted derivative **10b** was the most effective inhibitor with a *K*_I_ of 2.8 μM, whereas additional substitutions spoiled the potency (i.e., compounds **10c**–**g**). It is worth noting the strong halogen effect on the inhibition potency (i.e., **10e**–**g**, which well correlated with the electron negativity of the atom. As reported in Table 3, the fluoro containing compound **10e** resulted 1.4-fold more effective when compared to the chloro derivative **10g** (i.e., *K*_I_s of 4.9 and 6.75 μM for **10e** and **10g**, respectively), which in turn resulted 1.2-fold more effective than the iodo counterpart **10f** (i.e., *K*_I_ of 8.2 μM). Substitution of the fluoro in **10e** with a nitro moiety at the same position further enhanced the compound potency in inhibiting the MreCA isozyme (i.e., *K*_I_s of 4.9 and 4.56 μM for **4e** and **4d**, respectively). As expected, the compounds devoid of the either primary or secondary sulphanilamide moiety (i.e., **14c**, **14d**, and **16a**–**d**) were uneffective inhibitors on the panel of *Malassezia spp* expressed CAs.

Overall, the kinetic data reported in this study reasonably account for a valid contribution of the CAs in the yeast growth inhibition when treatments with compounds bearing primary/secondary sulfonamide moieties were considered.

## Conclusions

Selected acyl-/selenoureido containing derivatives belonging to the series herein reported were evaluated for their activity on *C. albicans*, *C. glabrata* and *M. pachydermatis* strains and showed excellent antifungal activity and selectivity against the latter. Subsequently, all the series were screened against *Malassezia spp*. of human and veterinary interest (i.e., *M. pachydermatis*, *M. globosa*, and *M. furfur*) and showed very potent antifungal features being associated with low MIC values comparable to the standard of care antifungal agents such as Ketoconazole, SeS_2_ and Amphotericin B. Interestingly, most compounds showed preferential activity against the *M. pachydermatis* strain over *M. globosa* and *M. furfur*. Few derivatives (i.e., **7b**, **7d**, **9b**, **9f**, and **16a**) were devoid of appreciable selectivity being effective on *M. pachydermatis* and *M. furfur* with comparable magnitude. The cytotoxicity profile of the selected compounds, particularly effective on *M. pachydermatis*, was assessed and exhibited safety features at the same MIC concentration. Deleterious effects were observed when concentrations close to 10.0-fold the antifungal concentration were considered. A further selection of these compounds did not show cytotoxicity against human keratinocytes cells at the same antifungal concentration (0.5 μg/mL). Notably, compounds **10c** and **11b** resulted safer than the clinically approved drug ketoconazole at each tested concentration, and all selected compounds proved less cytotoxic than SeS_2_. Very low hemolytic activities were observed for compounds chosen and tested at their MIC concentrations. **7c**, **7d**, **11e**, and **11i** determined <10% hemolysis at about 500-times the effective concentration (i.e., 256 μg/mL). In contrast, shallow hemolytic events (i.e., < 5%) were observed for the remaining derivatives when tested at the MIC concentration. The *in vitro* enzymatic assays of the compound series on the *Malassezia spp*. expressed CAs reported that all compounds bearing either a primary or a secondary sulfonamide moiety were endowed with inhibition features with *K*_I_ values spanning the medium-high nanomolar range.

All data reported in this study give support that such compounds are worth considering valid candidates for future development as novel antifungal drugs. The marked antifungal activities observed were immediately ascribed to the yeast-directed selenium toxicity mechanism, whereas the contribution that arose from inhibition of the yeast expressed CAs was not as much as evident but not absent and thus not to be ruled out. Such a discrepancy between two distinct and coexisting mechanisms is expected since the compounds here reported act on the uptake and metabolic transformations of selenium from yeasts, which is very fast and thus resulting in effective, immediate, and measurable effects. In comparison, inhibition of the yeast expressed CAs is slower and still of impact in consideration of its novelty as druggable target in order to tackle drug resistant yeast emerging strains.

## Methods

### General

Anhydrous solvents and all reagents were purchased from Sigma-Aldrich, VWR, and TCI. All reactions involving air- or moisture-sensitive compounds were performed under a nitrogen atmosphere. Nuclear magnetic resonance (^1^H NMR, ^13^C NMR, and ^77^Se NMR) spectra were recorded using a Bruker Advance III 400 MHz spectrometer in DMSO-*d*_6_ or CDCl_3_. Chemical shifts are reported in parts per million (ppm), and the coupling constants (*J*) are expressed in Hertz (Hz). Splitting patterns are designated as follows: s, singlet; d, doublet; t, triplet; m, multiplet; brs, broad singlet; dd, double of doubles. The assignment of exchangeable protons (N*H*) was confirmed by the addition of D_2_O. Analytical thin-layer chromatography (TLC) was carried out on Merck silica gel F-254 plates. Flash chromatography purifications were performed on Merck silica gel 60 (230–400 mesh ASTM) as the stationary phase, and ethyl acetate, *n*-hexane, acetonitrile, and methanol were used as eluents. The solvents used in MS measurements were acetone, acetonitrile (Chromasolv grade), purchased from Sigma-Aldrich (Milan, Italy), and mQ water 18 MΩ, obtained from Millipore’s Simplicity system (Milan, Italy). The mass spectra were obtained using a Varian 1200L triple quadrupole system (Palo Alto, CA, USA) equipped with electrospray source (ESI) operating in both positive and negative ions. Stock solutions of analytes were prepared in acetone at 1.0 mg mL^–1^ and stored at 4 °C. Working solutions of each analyte were freshly prepared by diluting stock solutions in a mixture of mQ H_2_O/ACN 1/1 (*v*/*v*) up to a concentration of 1.0 μg mL^–1^. The mass spectra of each analyte were acquired by introducing, via syringe pump at 10/L min^–1^, the working solution. Raw data were collected and processed by Varian Workstation, version 6.8, software. All compounds reported here are >95% of purity.

#### General Procedure for the Synthesis of Selenoureido Derivatives (**3a**–**e**, **4a**,**b**, **5a**–**h**)

The appropriate isoselenocyanate (**1a**–**i**) (1 equiv) was dissolved in acetonitrile and treated with the corresponding benzenesulfonamide **2a**–**c** (1 equiv). The mixture was stirred overnight at r.t., quenched with H_2_O, and the readily formed precipitate was collected by filtration and dried on air to afford the titled selenoureas **3**–**5**. Experimental data were in agreement with reported data.^19−23^

#### General Procedure for Preparation of Acylselenoureido Benzensulfonamide Derivatives (**7a**–**k**, **8a**–**g**, **9a**–**f**, **10a**–**g**, **11a**–**j**, **12**)

Potassium selenocyanate (1 equiv) dissolved in acetone (5.0 mL) was treated with the appropriate acyl chloride **6a**–**k** (1 equiv). The reaction mixture was stirred at r.t. for 15 min, followed by addition of appropriate aminobenzensulfonamide (**2a**–**f**) (1 equiv) and stirred for 40 min at the same temperature. After this time the mixture was quenched with H_2_O, and the formed precipitate was filtered-off and dried on air. The obtained products **7**–**12** were used as they are. Experimental data for compounds **7a**–**e**, **8a**, **9a**, **9c**, **11a**, **11b**, **11e**, **11i**, and **11j** were in agreement with reported data.^19−23^

### 4-Nitro-*N*-((4-sulfamoylphenyl)carbamoselenoyl)benzamide (**7f**)

Orange solid yield 87%; m.p.: 192–195 °C. Rf: 0.33 (50% EtOAc/n-hex); ^1^H NMR (DMSO-*d*_*6*_, 400 MHz): 12.86 (1H, bs, N*H*, exchange with D_2_O), 12.36 (1H, bs, N*H*, exchange with D_2_O), 8.40 (2H, d, *J* = 8.30 Hz), 8.21 (2H, d, *J* = 8.45 Hz), 7.90 (4H, aps), 7.47 (2H, bs, N*H*, exchange with D_2_O); ^13^C NMR (DMSO-*d*_6_, 100 MHz): 181.8, 167.3, 150.8, 142.8, 138.8, 131.3, 127.2, 126.4, 124.3, 120.4; ^77^Se-NMR (DMSO-*d*_6_, 76 MHz): 450.2; ESI-HRMS (*m*/*z*) calculated for [M-H]^−^ ion species C_14_H_11_N_4_O_5_SSe 427.9694, found 427.9692.

### 4-Iodo-*N*-((4-sulfamoylphenyl)carbamoselenoyl)benzamide (**7g**)

Yellow solid yield 77%; m.p.: 213-216 °C; ^1^H NMR (DMSO-*d*_6_, 400 MHz): 12.96 (1H, bs, N*H*, exchange with D_2_O), 12.06 (1H, bs, N*H*, exchange with D_2_O), 7.98 (2H, d, *J* = 8.27 Hz), 7.89 (4H, aps), 7.78 (2H, d, *J* = 8.21 Hz), 7.47 (2H, bs, N*H*, exchange with D_2_O); ^13^C NMR (DMSO-*d*_6_, 100 MHz): 181.9, 168.3, 142.8, 138.2, 132.3, 131.9, 131.5, 127.2, 126.4, 102.6; ^77^Se-NMR (DMSO-*d*_6_, 76 MHz): 440.2; ESI-HRMS (*m*/*z*) calculated for [M-H]^−^ ion species C_14_H_11_IN_3_O_3_SSe 508.8809, found 508.8806.

### 4-Bromo-2-fluoro-*N*-((4-sulfamoylphenyl)carbamoselenoyl)benzamide (**7h**)

Yellow solid yield 57%; m.p.: 211–214 °C; Rf: 0.18 (1% MeOH/DCM); ^1^H NMR (DMSO-*d*_6_, 400 MHz): 12.73 (1H, bs, N*H*, exchange with D_2_O), 12.21 (1H, bs, N*H*, exchange with D_2_O), 7.89 (4H, aps), 7.82 (1H, d, *J* = 9.96 Hz), 7.71 (1H, t, *J* = 7.67 Hz), 7.63 (1H, d, *J* = 8.15 Hz), 7.48 (2H, bs, N*H*, exchange with D_2_O); ^13^C NMR (DMSO-*d*_6_, 100 MHz): 181.3, 165.0, 160.2 (d, *J* = 255.92 Hz), 142.9 (d, *J* = 30.23 Hz), 132.9, 128.8, 127.7, 127.2, 126.6, 122.3 (d, *J* = 14.05 Hz), 120.6 (d, *J* = 25.13 Hz); ^19^F-NMR (DMSO-*d*_6_, 376 MHz): −109.55; ^77^Se-NMR (DMSO-*d*_6_, 76 MHz): 442.1; ESI-HRMS (*m*/*z*) calculated for [M-H]^−^ ion species C_14_H_10_BrFN_3_O_3_SSe 478.8854, found 478.8853.

### 4-(Chloromethyl)-*N*-((4-sulfamoylphenyl)carbamoselenoyl)benzamide (**7i**)

Yellow solid 66% yield; m.p.: 212–215 °C; Rf: 0.14 (1% MeOH/DCM); ^1^H NMR (DMSO-*d*_6_, 400 MHz): 13.03 (1H, bs, N*H*, exchange with D_2_O), 12.03 (1H, bs, N*H*, exchange with D_2_O), 8.02 (2H, d, *J* = 8.06 Hz), 7.90 (4H, aps), 7.65 (2H, d, *J* = 8.15 Hz), 7.48 (2H, bs, N*H*, exchange with D_2_O), 4.90 (2H, s); ^13^C NMR (DMSO-*d*_6_, 100 MHz): 181.9, 168.3, 143.7, 142.8, 132.6, 130.1, 129.8, 129.6, 127.2, 126.4, 46.1; ^77^Se-NMR (DMSO-*d*_6_, 76 MHz): 437.8; ESI-HRMS (*m*/*z*) calculated for [M-H]^−^ ion species C_14_H_11_ClN_3_O_3_SSe 416.9453, found 416.9450.

### (*E*)-4-(Phenyldiazenyl)-*N*-((4-sulfamoylphenyl)carbamoselenoyl)benzamide (**7j**)

Orange solid 80% yield; m.p.: 214–217 °C; Rf: 0.48 (50% EtOAc/n-hex); ^1^H NMR (DMSO-*d*_6_, 400 MHz): 13.00 (1H, bs, N*H*, exchange with D_2_O), 12.18 (1H, bs, N*H*, exchange with D_2_O), 8.23 (2H, d, *J* = 8.02 Hz), 8.05 (2H, d, *J* = 8.34 Hz), 8.00 (2H, bs), 7.92 (4H, aps), 7.68 (3H, m), 7.48 (2H, bs, N*H*_2_, exchange with D_2_O); ^13^C NMR (DMSO-*d*_6_, 100 MHz): 181.9, 168.0, 155.2, 152.8, 142.9, 142.8, 134.9, 133.3, 131.3, 130.5, 127.2, 126.5, 123.8, 123.2; ^77^Se-NMR (DMSO-*d*_6_, 76 MHz): 441.3; ESI-HRMS (*m*/*z*) calculated for [M-H]^−^ ion species C_20_H_16_N_5_O_3_SSe 487.0217, found 487.0215.

### 4-Fluoro-*N*-((4-sulfamoylphenyl)carbamoselenoyl)benzamide (**7k**)

Orange solid yield 77%; m.p.: 208–211 °C; Rf: 0.18 (1% MeOH/DCM); ^1^H NMR (DMSO-*d*_6_, 400 MHz): 12.99 (1H, bs, N*H*, exchange with D_2_O), 12.05 (1H, bs, N*H*, exchange with D_2_O), 8.10 (2H, m), 7.90 (4H, m), 7.46 (4H, m); ^13^C NMR (DMSO-*d*_6_, 100 MHz): 182.0, 158.8, 165.9 (d, *J* = 251.98 Hz), 142.9, 132.8 (d, *J* = 9.46 Hz), 127.8, 127.2, 126.4, 120.4, 116.4 (d, *J* = 22.08 Hz); ESI-HRMS (*m*/*z*) calculated for [M-H]^−^ ion species C_14_H_11_FN_3_O_3_SSe 400.9749, found 400.9751.

### 4-Heptyl-*N*-((3-sulfamoylphenyl)carbamoselenoyl)benzamide (**8b**)

Orange solid 93% yield; m.p.: 138–141 °C; ^1^H NMR (DMSO-*d*_6_, 400 MHz): 13.06 (1H, bs, N*H*, exchange with D_2_O), 11.86 (1H, bs, N*H*, exchange with D_2_O), 8.15 (1H, s), 7.96 (3H, m), 7.79 (1H, bs), 7.67 (1H, bs), 7.51 (2H, bs), 7.42 (2H, bs, N*H*_2_, exchange with D_2_O), 2.71 (2H, t, *J* = 7.48 Hz), 1.64 (2H, m), 1.32 (8H, m), 0.90 (3H, t, *J* = 6.53 Hz); ^13^C NMR (DMSO-*d*_6_, 100 MHz): 182.2, 168.8, 149.5, 145.5, 140.5, 130.3, 130.1, 129.9, 129.7, 129.3, 124.8, 123.4, 36.0, 32.2, 31.5, 29.6, 29.5, 23.0, 14.9; ^77^Se-NMR (DMSO-*d*_6_, 76 MHz): 425.6; ESI-HRMS (*m*/*z*) calculated for [M-H]^−^ ion species C_21_H_26_N_3_O_3_SSe 481.0938, found 481.0937.

### 3-Bromo-*N*-((3-sulfamoylphenyl)carbamoselenoyl)-5-(trifluoromethoxy)benzamide (**8c**)

Red solid 76% yield; m.p.: 113–115 °C; ^1^H NMR (DMSO-*d*_6_, 400 MHz): 12.77 (1H, bs, N*H*, exchange with D_2_O), 12.29 (1H, bs, N*H*, exchange with D_2_O), 8.26 (1H, s), 8.12 (1H, s), 8.06 (1H, s), 7.97 (1H, s), 7.91 (1H, d, *J* = 6.69 Hz), 7.81 (1H, d, *J* = 7.34 Hz), 7.67 (1H, t, *J* = 7.70 Hz), 7.52 (2H, bs, N*H*_2_, exchange with D_2_O); ESI-HRMS (*m*/*z*) calculated for [M-H]^−^ ion species C_14_H_11_BrN_3_O_3_SSe 460.8948, found 460.8950.

### 4-Fluoro-*N*-((3-sulfamoylphenyl)carbamoselenoyl)benzamide (**8d**)

Green solid 81% yield; m.p.: 185–188 °C; Rf: 0.2 (40% EtOAc/n-hex); ^1^H NMR (DMSO-*d*_6_, 400 MHz): 12.95 (1H, bs, N*H*, exchange with D_2_O), 12.04 (1H, bs, N*H*, exchange with D_2_O), 8.14–8.09 (3H, m), 7.92 (1H, d, *J* = 7.59 Hz), 7.80 (1H, d, *J* = 7.61 Hz), 7.67 (1H, t, *J* = 7.85 Hz), 7.52 (2H, bs, N*H*_2_, exchange with D_2_O), 7.43 (2H, t, *J* = 8.76 Hz); ^13^C NMR (DMSO-*d*_6_, 100 MHz): 182.2, 167.8, 165.9 (d, *J* = 251.68 Hz), 145.5, 140.5, 132.8 (d, *J* = 9.54 Hz), 130.3, 129.7, 129.4, 124.9, 123.5, 116.5 (d, *J* = 22.10 Hz); ^19^F-NMR (DMSO-*d*_6_, 376 MHz): −105.79; ^77^Se-NMR (DMSO-*d*_6_, 76 MHz): 430.2; ESI-HRMS (*m*/*z*) calculated for [M-H]^−^ ion species C_14_H_11_FN_3_O_3_SSe 400.9749, found 400.9747.

### 4-Iodo-*N*-((3-sulfamoylphenyl)carbamoselenoyl)benzamide (**8e**)

Orange solid 70% yield; m.p.: 213-216 °C. Rf: 0.33 (45% EtOAc/n-hex); ^1^H NMR (DMSO-*d*_6_, 400 MHz): 12.92 (1H, bs, N*H*, exchange with D_2_O), 12.05 (1H, bs, N*H*, exchange with D_2_O), 8.13 (1H, s), 7.98 (2H, d, *J* = 8.36 Hz), 7.92 (1H, d, *J* = 7.40 Hz), 7.79 (3H, m), 7.66 (1H, t, *J* = 7.87 Hz), 7.52 (2H, bs, N*H*_2_, exchange with D_2_O); ^13^C NMR (DMSO-*d*_6_, 100 MHz):182.1, 168.3, 145.5, 140.4, 138.2, 132.3, 131.5, 120.3, 129.6, 124.8, 123.4, 102.6; ^77^Se-NMR (DMSO-*d*_6_, 76 MHz): 434.7; ESI-HRMS (*m*/*z*) calculated for [M-H]^−^ ion species C_14_H_11_IN_3_O_3_SSe 508.8809, found 508.8806.

### 4-Chloro-*N*-((3-sulfamoylphenyl)carbamoselenoyl)benzamide (**8f**)

Yellow solid 75% yield; m.p.: 179–182 °C; Rf: 0.2 (3% MeOH/DCM).; ^1^H NMR (DMSO-*d*_6_, 400 MHz): 12.92 (1H, bs, N*H*, exchange with D_2_O), 12.07 (1H, bs, N*H*, exchange with D_2_O), 8.14 (1H, s), 8.03 (2H, d, *J* = 8.76 Hz), 7.92 (1H, d, *J* = 7.41 Hz), 7.80 (1H, d, *J* = 7.61 Hz), 7.68.7.66 (3H, m), 7.51 (2H, bs, N*H*_2_, exchange with D_2_O); ^13^C NMR (DMSO-*d*_6_, 100 MHz): 182.2, 167.8, 145.5, 140.4, 139.1, 131.7, 130.6, 130.3, 129.7, 129.5, 124.9, 123.5; ^77^Se-NMR (DMSO-*d*_6_, 76 MHz): 433.8; ESI-HRMS (*m*/*z*) calculated for [M-H]^−^ ion species C_14_H_11_ClN_3_O_3_SSe 416.9453, found 416.9450.

### 4-Bromo-2-fluoro-*N*-((3-sulfamoylphenyl)carbamoselenoyl)benzamide (**8g**)

Yellow solid 75% yield; m.p.: 213-216 °C; Rf: 0.13 (1% MeOH/DCM).; ^1^H NMR (DMSO-*d*_6_, 400 MHz): 12.70 (1H, bs, N*H*, exchange with D_2_O), 12.20 (1H, bs, N*H*, exchange with D_2_O), 8.12 (1H, s), 7.90 (1H, d, *J* = 6.73 Hz), 7.82 (2H, d, *J* = 5.20 Hz), 7.73–7.62 (3H, m), 7.52 (2H, bs, N*H*_2_, exchange with D_2_O); ^13^C NMR (DMSO-*d*_6_, 100 MHz): 181.5, 164.9, 160.2 (d, *J* = 255.96 Hz), 145.5, 140.4, 132.9, 130.3, 129.9, 128.8, 127.2 (d, *J* = 9.76 Hz), 125.0, 123.6, 122.3 (d, *J* = 13.89 Hz), 120.6 (d, *J* = 25.03 Hz); ^19^F-NMR (DMSO-*d*_6_, 376 MHz): −109.56; ^77^Se-NMR (DMSO-*d*_6_, 76 MHz): 436.5; ESI-HRMS (*m*/*z*) calculated for [M-H]^−^ ion species C_14_H_10_BrFN_3_O_3_SSe 478.8854, found 478.8852.

### 4-Heptyl-*N*-((4-sulfamoylphenethyl)carbamoselenoyl)benzamide (**9b**)

Orange solid 71% yield; m.p.: 132–135 °C; Rf: 0.32 (40% EtOAc/n-hex); ^1^H NMR (DMSO-*d*_6_, 400 MHz): 11.50 (2H, bs, N*H*, exchange with D_2_O), 7.88 (2H, d, *J* = 7.37 Hz), 7.82 (2H, d, *J* = 7.38 Hz), 7.53 (2H, d, *J* = 7.33 Hz), 7.35 (4H, m), 3.98 (2H, m), 3.12 (2H, t, *J* = 7.3 Hz), 2.68 (2H, t, *J* = 7.5 Hz), 1.62 (2H, m), 1.31 (8H, m), 0.89 (3H, t, *J* = 6.5 Hz); ^13^C NMR (DMSO-*d*_6_, 100 MHz): 181.7, 168.7, 149.2, 143.7, 143.3, 130.1, 120.0, 129.7, 129.2, 126.8, 49.7, 36.0, 34.2, 32.2, 31.5, 29.5, 29.4, 23.0, 14.8; ^77^Se-NMR (DMSO-*d*_6_, 76 MHz): 348.6; ESI-HRMS (*m*/*z*) calculated for [M-H]^−^ ion species C_23_H_30_N_3_O_3_SSe 509.1251, found 509.1253.

### 4-Fluoro-*N*-((4-sulfamoylphenethyl)carbamoselenoyl)benzamide (**9d**)

Orange solid 78% yield; m.p.: 144–147 °C; Rf: 0.17 (1% MeOH/DCM); ^1^H NMR (DMSO-*d*_6_, 400 MHz): 11.70 (1H, bs, N*H*, exchange with D_2_O), 11.44 (1H, bs, N*H*, exchange with D_2_O), 8.04–8.01 (2H, m), 7.82 (2H, d, *J* = 8.14 Hz), 7.53 (2H, d, *J* = 8.15 Hz), 7.41–7.36 (4H, m), 3.98 (2H, m), 3.12 (2H, t, *J* = 7.27 Hz); ^13^C NMR (DMSO-*d*_6_, 100 MHz): 181.6, 167.9, 165.8 (d, *J* = 251.31 Hz), 143.8, 143.3, 132.7 (d, *J* = 9.47 Hz), 130.1, 129.4, 126.8, 116.4 (d, *J* = 21.80 Hz), 49.7, 34.1; ESI-HRMS (*m*/*z*) calculated for [M-H]^−^ ion species C_16_H_15_FN_3_O_3_SSe 509.1251, found 509.1250.

### 4-Iodo-*N*-((4-sulfamoylphenethyl)carbamoselenoyl)benzamide (**9e**)

Red solid 78% yield; m.p.: 210–213 °C; Rf: 0.28 (45% EtOAc/n-hex); ^1^H NMR (DMSO-*d*_6_, 400 MHz): 11.74 (1H, bs, N*H*, exchange with D_2_O), 11.41 (1H, bs, N*H*, exchange with D_2_O), 7.94 (2H, d, *J* = 8.20 Hz), 7.81 (2H, d, *J* = 8.02 Hz), 7.70 (2H, d, *J* = 8.19 Hz), 7.52 (2H, d, *J* = 7.94 Hz), 7.36 (2H, bs, N*H*, exchange with D_2_O), 3.97 (2H, m), 3.12 (2H, t, *J* = 7.09 Hz); ^13^C NMR (DMSO-*d*_6_, 100 MHz): 181.5, 168.3, 143.7, 143.3, 138.2, 132.3, 131.4, 130.0, 126.8, 102.4, 49.7, 34.1; ^77^Se-NMR (DMSO-*d*_6_, 76 MHz): 354.4; ESI-HRMS (*m*/*z*) calculated for [M-H]^−^ ion species C_16_H_15_IN_3_O_3_SSe 536.9122, found 536.9125.

### (*E*)-4-(Phenyldiazenyl)-*N*-((4-sulfamoylphenethyl)carbamoselenoyl)benzamide (**9f**)

Orange solid 88% yield; m.p.: 235–238 °C; Rf: 0.21 (40% EtOAc/n-hex); ^1^H NMR (DMSO-*d*_6_, 400 MHz): 11.86 (1H, bs, N*H*, exchange with D_2_O), 11.46 (1H, bs, N*H*, exchange with D_2_O), 8.15 (2H, d, *J* = 8.38 Hz), 8.02–7.98 (5H, m), 7.83 (2H, d, *J* = 8.03 Hz), 7.68–7.67 (4H, m), 7.55 (2H, d, *J* = 8.04 Hz), 7.37 (2H, bs, N*H*, exchange with D_2_O), 4.00 (2H, m), 3.14 (2H, t, *J* = 7.11 Hz); ^13^C NMR (DMSO-*d*_6_, 100 MHz): 181.5, 168.1, 155.1, 152.8, 143.7, 143.3, 134.9, 133.2, 131.1, 130.5, 130.0, 126.8, 123.8, 123.1, 49.7, 34.1; ^77^Se-NMR (DMSO-*d*_6_, 76 MHz): 357.1; ESI-HRMS (*m*/*z*) calculated for [M-H]^−^ ion species C_22_H_20_N_5_O_3_SSe 515.0530, found 515.0531.

### *N*-((4-(*N*-(Thiazol-2-yl)sulfamoyl)phenyl)carbamoselenoyl)benzamide (**10a**)

Yellow solid 85% yield; m.p.: 231–233 °C; Rf: 0.22 (3% MeOH/DCM); ^1^H NMR (DMSO-*d*_6_, 400 MHz): 13.03 (1H, bs, N*H*, exchange with D_2_O), 12.83 (1H, bs, N*H*, exchange with D_2_O), 11.98 (1H, bs, N*H*, exchange with D_2_O), 8.01 (2H, d, *J* = 7.48 Hz), 7.88 (4H, aps), 7.72 (1H, t, *J* = 7.39 Hz), 7.59 (2H, t, *J* = 7.61 Hz), 7.32 (1H, d, *J* = 4.60 Hz), 6.90 (1H, d, *J* = 4.56 Hz); ^13^C NMR (DMSO-*d*_6_, 100 MHz): 181.8, 169.8, 143.0, 141.1, 134.2, 132.8, 129.7, 129.4, 127.3, 126.3, 125.4, 113.3, 109.3; ^77^Se-NMR (DMSO-*d*_6_, 76 MHz): 436.9; ESI-HRMS (*m*/*z*) calculated for [M-H]^−^ ion species C_17_H_13_N_4_O_3_S_2_Se 465.9673, found 465.9670.

### 3-Bromo-*N*-((4-(*N*-(thiazol-2-yl)sulfamoyl)phenyl)carbamoselenoyl)-5-(trifluoromethoxy)benzamide (**10b**)

Yellow solid 77% yield; m.p.: 208–211 °C; Rf: 0.17 (50% EtOAc/n-hex); ^1^H NMR (DMSO-*d*_6_, 400 MHz): 12.84 (1H, bs, N*H*, exchange with D_2_O), 12.80 (1H, bs, N*H*, exchange with D_2_O), 12.29 (1H, bs, N*H*, exchange with D_2_O), 8.24 (1H, s), 7.88 (4H, aps), 8.05 (1H, s), 7.96 (1H, s), 7.87 (4H, aps), 7.32 (1H, d, *J* = 4.54 Hz), 6.90 (1H, d, *J* = 4.52 Hz); ^13^C NMR (DMSO-*d*_6_, 100 MHz): 181.6, 169.8, 149.3, 142.9, 136.7, 131.8, 129.4, 127.9, 127.4, 126.3, 125.4, 123.1, 121.5, 120.4, 109.3; ^19^F-NMR (DMSO-*d*_6_, 376 MHz): −56.88; ^77^Se-NMR (DMSO-*d*_6_, 76 MHz): 453.7; ESI-HRMS (*m*/*z*) calculated for [M-H]^−^ ion species C_18_H_11_BrF_3_N_4_O_4_S_2_Se 627.8601, found 627.8603.

### 4-Heptyl-*N*-((4-(*N*-(thiazol-2-yl)sulfamoyl)phenyl)carbamoselenoyl)benzamide (**10c**)

Yellow solid 81% yield; m.p.: 204–207 °C; Rf: 0.1 (1% MeOH/DCM); ^1^H NMR (DMSO-*d*_6_, 400 MHz): 13.08 (1H, bs, N*H*, exchange with D_2_O), 12.85 (1H, bs, N*H*, exchange with D_2_O), 11.86 (1H, bs, N*H*, exchange with D_2_O), 7.95 (2H, d, *J* = 7.98 Hz), 7.87 (4H, aps), 7.40 (2H, d, *J* = 8.09 Hz), 7.32 (1H, d, *J* = 4.60 Hz), 6.90 (1H, d, *J* = 4.58 Hz), 2.70 (2H, t, *J* = 7.52 Hz), 1.63 (2H, m), 1.33–1.29 (8H, m), 0.89 (3H, t, *J* = 6.64 Hz); ^13^C NMR (DMSO-*d*_6_, 100 MHz): 181.8, 169.9, 149.5, 143.0, 141.1, 130.1, 129.9, 129.3, 127.3, 126.3, 125.4, 109.3, 36.0, 32.2, 31.5, 29.5, 29.4, 23.0, 14.9; ESI-HRMS (*m*/*z*) calculated for [M-H]^−^ ion species C_24_H_27_N_4_O_3_S_2_Se 564.0768, found 564.0769.

### 4-Nitro-*N*-((4-(*N*-(thiazol-2-yl)sulfamoyl)phenyl)carbamoselenoyl)benzamide (**10d**)

Red solid 76% yield; m.p.: 218–221 °C; Rf: 0.2 (60% EtOAc/n-hex); ^1^H NMR (DMSO-*d*_6_, 400 MHz): 12.84 (2H, bs, N*H*, exchange with D_2_O), 12.36 (1H, bs, N*H*, exchange with D_2_O), 8.39 (2H, d, *J* = 8.40 Hz), 8.20 (2H, d, *J* = 8.15 Hz), 7.88 (4H, aps), 7.32 (1H, d, *J* = 4.36 Hz), 6.90 (1H, d, *J* = 4.43 Hz); ESI-HRMS (*m*/*z*) calculated for [M-H]^−^ ion species C_17_H_12_N_5_O_5_S_2_Se 564.0768, found 564.0773.

### 4-Fluoro-*N*-((4-(*N*-(thiazol-2-yl)sulfamoyl)phenyl)carbamoselenoyl)benzamide (**10e**)

Yellow solid 80% yield; m.p.: 216–219 °C; ^1^H NMR (DMSO-*d*_6_, 400 MHz): 12.99 (1H, bs, N*H*, exchange with D_2_O), 12.85 (1H, bs, N*H*, exchange with D_2_O), 12.04 (1H, bs, N*H*, exchange with D_2_O), 8.11–8.07 (2H, m), 7.88 (4H, aps), 7.42 (2H, t, *J* = 8.80 Hz), 7.32 (1H, d, *J* = 4.56 Hz), 6.90 (1H, d, *J* = 4.56 Hz); ^13^C NMR (DMSO-*d*_6_, 100 MHz): 181.8, 169.8, 165.9 (d, *J* = 251.71 Hz), 143.0, 141.1, 132.8 (d, *J* = 9.53 Hz), 127.7, 127.3, 126.3, 125.4, 120.7, 116.4 (d, *J* = 22.12 Hz), 109.3; ^19^F-NMR (DMSO-*d*_6_, 376 MHz): −105.75; ^77^Se-NMR (DMSO-*d*_6_, 76 MHz): 437.7; ESI-HRMS (*m*/*z*) calculated for [M-H]^−^ ion species C_17_H_12_FN_4_O_3_S_2_Se 483.9578, found 483.9580.

### 4-Iodo-*N*-((4-(*N*-(thiazol-2-yl)sulfamoyl)phenyl)carbamoselenoyl)benzamide (**10f**)

Yellow solid 78% yield; m.p.: 199–202 °C; Rf: 0.2 (60% EtOAc/n-hex); ^1^H NMR (DMSO-*d*_6_, 400 MHz): 12.95 (1H, bs, N*H*, exchange with D_2_O), 12.84 (1H, bs, N*H*, exchange with D_2_O), 12.03 (1H, bs, N*H*, exchange with D_2_O), 7.97 (2H, d, *J* = 8.32 Hz), 7.87 (4H, aps), 7.77 (2H, d, *J* = 8.27 Hz), 7.32 (1H, d, *J* = 4.57 Hz), 6.89 (1H, d, *J* = 4.55 Hz); ^13^C NMR (DMSO-*d*_6_, 100 MHz): 181.7, 169.8, 142.9, 141.1, 138.2, 132.2, 131.9, 131.5, 127.3, 126.3, 125.4, 109.2, 102.6; ^77^Se-NMR (DMSO-*d*_6_, 76 MHz): 442.0; ESI-HRMS (*m*/*z*) calculated for [M-H]^−^ ion species C_17_H_12_IN_4_O_3_S_2_Se 591.8639, found 591.8644

### 4-Chloro-*N*-((4-(*N*-(thiazol-2-yl)sulfamoyl)phenyl)carbamoselenoyl)benzamide (**10g**)

Yellow solid 51% yield; m.p.: 215–217 °C; Rf: 0.1 (3% MeOH/DCM). ^1^H NMR (DMSO-*d*_6_, 400 MHz): 12.94 (1H, bs, N*H*, exchange with D_2_O), 12.84 (1H, bs, N*H*, exchange with D_2_O), 12.08 (1H, bs, N*H*, exchange with D_2_O), 8.02 (2H, d, *J* = 8.44 Hz), 7.87 (4H, aps), 7.66 (2H, d, *J* = 8.51 Hz), 7.32 (1H, d, *J* = 4.60 Hz), 6.90 (1H, d, *J* = 4.59 Hz); ^13^C NMR (DMSO-*d*_6_, 100 MHz): 181.8, 169.8, 143.0, 141.1, 139.0, 131.7, 129.4, 127.3, 126.3, 125.4, 109.3; ^77^Se-NMR (DMSO-*d*_6_, 76 MHz): 442.1; ESI-HRMS (*m*/*z*) calculated for [M-H]^−^ ion species C_17_H_12_ClN_4_O_3_S_2_Se 499.9283, found 499.9281

### *N*-((2-Hydroxy-5-sulfamoylphenyl)carbamoselenoyl)-4-nitrobenzamide (**11c**)

Orange solid 80% yield; m.p.: 185–187 °C; Rf: 0.22 (60% EtOAc/n-hex); ^1^H NMR (DMSO-*d*_6_, 400 MHz): 13.24 (1H, bs, N*H*, exchange with D_2_O), 12.36 (1H, bs, N*H*, exchange with D_2_O), 11.23 (1H, bs, O*H*, exchange with D_2_O), 9.12 (1H, s), 8.39 (2H, d, *J* = 8.71 Hz), 8.21 (2H, d, *J* = 8.67 Hz), 7.66 (1H, dd, *J* = 8.49, 1.69 Hz), 7.29 (2H, bs, N*H*_*2*_, exchange with D_2_O), 7.13 (1H, d, *J* = 8.55 Hz); ^13^C NMR (DMSO-*d*_6_, 100 MHz): 179.8, 167.8, 153.3, 150.8, 138.7, 135.0, 131.3, 127.1, 126.4, 124.2, 122.9, 116.3; ^77^Se-NMR (DMSO-*d*_6_, 76 MHz): 451.3; ESI-HRMS (*m*/*z*) calculated for [M-H]^−^ ion species C_14_H_11_N_4_O_6_SSe 443.9643, found 443.9642

### *N*-((2-Hydroxy-5-sulfamoylphenyl)carbamoselenoyl)-4-iodobenzamide (**11d**)

Yellow solid 82% yield; m.p.: 261–264 °C; ^1^H NMR (DMSO-*d*_6_, 400 MHz): 13.32 (1H, bs, N*H*, exchange with D_2_O), 12.01 (1H, bs, N*H*, exchange with D_2_O), 11.19 (1H, bs, O*H*, exchange with D_2_O), 9.10 (1H, d, *J* = 1.69 Hz), 7.98 (2H, d, *J* = 8.33 Hz), 7.77 (2H, d, *J* = 8.37 Hz), 7.65 (1H, dd, *J* = 8.55, 2.00 Hz), 7.27 (2H, bs, N*H*_*2*_, exchange with D_2_O), 7.12 (1H, d, *J* = 8.55 Hz); ^13^C NMR (DMSO-*d*_6_, 100 MHz): 179.9, 168.7, 153.3, 138.2, 134.9, 132.2, 131.5, 127.1, 126.3, 123.0, 116.2, 102.5; ^77^Se-NMR (DMSO-*d*_6_, 76 MHz): 440.4; ESI-HRMS (*m*/*z*) calculated for [M-H]^−^ ion species C_14_H_11_IN_3_O_4_SSe 524.8758, found 524.8756

### 4-Chloro-*N*-((2-hydroxy-5-sulfamoylphenyl)carbamoselenoyl)benzamide (**11f**)

Orange solid 43% yield; m.p.: 260–263 °C; Rf: 0.12 (5% MeOH/DCM); ^1^H NMR (DMSO-*d*_6_, 400 MHz): 13.32 (1H, bs, N*H*, exchange with D_2_O), 12.07 (1H, bs, N*H*, exchange with D_2_O), 11.20 (1H, bs, O*H*, exchange with D_2_O), 9.10 (1H, s), 8.02 (2H, d, *J* = 8.43 Hz), 7.66 (3H, d, *J* = 8.34 Hz), 7.28 (2H, bs, N*H*_*2*_, exchange with D_2_O), 7.12 (1H, d, *J* = 8.56 Hz); ^13^C NMR (DMSO-*d*_6_, 100 MHz): 180.0, 168.4, 153.3, 139.1, 135.0, 131.8, 131.6, 129.5, 127.2, 126.3, 123.0, 116.3; ^77^Se-NMR (DMSO-*d*_6_, 76 MHz): 439.4; ESI-HRMS (*m*/*z*) calculated for [M-H]^−^ ion species C_14_H_11_ClN_3_O_4_SSe 432.9402, found 432.9401

### 4-Bromo-2-fluoro-*N*-((2-hydroxy-5-sulfamoylphenyl)carbamoselenoyl)benzamide (**11g**)

Yellow solid 54% yield; m.p.: 252–255 °C; ^1^H NMR (DMSO-*d*_6_, 400 MHz): 13.09 (1H, bs, N*H*, exchange with D_2_O), 12.17 (1H, bs, N*H*, exchange with D_2_O), 11.27 (1H, bs, O*H*, exchange with D_2_O), 9.12 (1H, d, *J* = 1.47 Hz), 7.83 (1H, d, *J* = 9.47 Hz), 7.73 (1H, t, *J* = 7.92 Hz), 7.67–7.62 (2H, m), 7.29 (2H, bs, N*H*_*2*_, exchange with D_2_O), 7.12 (1H, d, *J* = 8.56 Hz); ^13^C NMR (DMSO-*d*_6_, 100 MHz):179.2, 165.3, 160.1 (d, *J* = 255.56 Hz), 153.2, 135.0, 132.9, 128.8. 127.2 (d, *J* = 9.97 Hz), 127.0, 126.3, 122.7, 122.2 (d, *J* = 13.95 Hz), 120.5 (d, *J* = 25.25 Hz), 116.2; ^19^F-NMR (DMSO-*d*_6_, 376 MHz): −109.64; ^77^Se-NMR (DMSO-*d*_6_, 76 MHz): 448.8; ESI-HRMS (*m*/*z*) calculated for [M-H]^−^ ion species C_14_H_10_BrFN_3_O_4_SSe 494.8803, found 494.8801.

### 4-(Chloromethyl)-*N*-((2-hydroxy-5-sulfamoylphenyl)carbamoselenoyl)benzamide (**11h**)

Yellow solid 74% yield; m.p.: 216–219 °C.); ^1^H NMR (DMSO-*d*_6_, 400 MHz): 13.37 (1H, bs, N*H*, exchange with D_2_O), 11.96 (1H, bs, N*H*, exchange with D_2_O), 11.19 (1H, bs, O*H*, exchange with D_2_O), 9.11 (1H, s), 8.03 (2H, d, *J* = 7.93 Hz), 7.66 (4H, apd, *J* = 7.82 Hz), 7.27 (2H, bs, N*H*_*2*_, exchange with D_2_O), 7.12 (1H, d, *J* = 8.54 Hz), 4.90 (2H, s); ^13^C NMR (DMSO-*d*_6_, 100 MHz): 180.0, 168.8, 153.3, 143.7, 134.9, 130.1, 129.8, 129.6, 127.1, 126.2, 122.9, 116.2, 46.1; ^77^Se-NMR (DMSO-*d*_6_, 76 MHz): 438.6; ESI-HRMS (*m*/*z*) calculated for [M-H]^−^ ion species C_15_H_13_ClN_3_O_4_SSe 446.9559, found 446.9561.

### 3-Bromo-*N*-((4-((4-sulfamoylbenzyl)selanyl)phenyl)carbamoselenoyl)-5-(trifluoromethoxy)benzamide (**12**)

Red solid 77% yield; m.p.: 92–95 °C.; ^1^H NMR (DMSO-*d*_6_, 400 MHz): 12.72 (1H, bs, N*H*, exchange with D_2_O), 12.21 (1H, bs, N*H*, exchange with D_2_O), 8.24 (1H, s), 8.05 (1H, s), 7.96 (1H, s), 7.75 (2H, d, *J* = 7.61 Hz), 7.64 (2H, d, *J* = 7.65 Hz), 7.57 (2H, d, *J* = 7.78 Hz), 7.50 (2H, d, *J* = 7.32 Hz), 7.33 (2H, bs, N*H*_*2*_, exchange with D_2_O), 4.38 (2H, s); ^13^C NMR (DMSO-*d*_6_, 100 MHz): 181.0, 165.8, 149.2, 143.9, 138.8, 136.7, 132.9, 131.8, 130.1, 129.3, 126.6, 126.5, 123.0, 122.1, 121.5, 30.7; ^19^F-NMR (DMSO-*d*_6_, 376 MHz): −56.86; ^77^Se-NMR (DMSO-*d*_6_, 76 MHz): 441.6, 373.6; ESI-HRMS (*m*/*z*) calculated for [M-H]^−^ ion species C_22_H_16_BrF_3_N_3_O_4_SSe_2_ 714.8406, found 714.8404.

### *In Vitro* Carbonic Anhydrase Inhibition

An Applied Photophysics stopped-flow instrument was used to assay the CA catalyzed CO_2_ hydration activity.^26^ Phenol red (at a concentration of 0.2 mM) was used as an indicator, working at the absorbance maximum of 557 nm, with 20 mM Hepes (pH 7.4) as a buffer, and 20 mM Na_2_SO_4_ (to maintain constant ionic strength), following the initial rates of the CA-catalyzed CO_2_ hydration reaction for a period of 10–100 s. The CO_2_ concentrations ranged from 1.7 to 17 mM for the determination of the kinetic parameters and inhibition constants. Enzyme concentrations ranged between 5 and 12 nM. For each inhibitor, at least six traces of the initial 5–10% of the reaction were used to determine the initial velocity. The uncatalyzed rates were determined in the same manner and subtracted from the total observed rates. Stock solutions of the inhibitor (0.1 mM) were prepared in distilled–deionized water, and dilutions up to 0.01 nM were done thereafter with the assay buffer. Inhibitor and enzyme solutions were preincubated together for 15 min at room temperature prior to the assay to allow for the formation of the E–I complex. The inhibition constants were obtained by nonlinear least-squares methods using PRISM 3 and the Cheng-Prusoff equation as reported earlier and represent the mean from at least three different determinations. All CA isoforms were recombinant proteins obtained in-house, as reported earlier.^27−30^

### *In Vitro* Antifungal Activity and Toxicity

All the media were purchased from Life Technologies Limited (Paisley, UK). Reference ATCC strains of *Malassezia globosa* and *Candida albicans* were purchased from ATCC 2021 (https://www.atcc.org), USA. The reference strain of *Malassezia furfur* was purchased from KAIROSafe S.R.L. (https://www.kairosafe.it). The reference strains of *Malassezia pachydermatis* and *Candida glabrata* were purchased from DSMZ (https://www.dsmz.de). MDBK ATCC CCL-22 cells were purchased from ATCC (https://www.atcc.org), USA. HaCat BS CL 168 cells were purchased from the Biobanking of Veterinary Resources of IZSLER (Brescia, Italy). Sheep defibrinated blood was purchased from Thermo Fisher Diagnostics S.p.A. (Milano, Italy).

### Antifungal Activity Evaluation Methods

Inoculum preparations for antifungal assays were performed following the CLSI guidelines for antifungal susceptibility testing of yeasts.^31^ Antifungal activity was evaluated on unicellular yeasts *Candida albicans* ATCC 10231, *Candida glabrata* ATCC 90030 (DSMZ 11226), *Malassezia pachydermatis* CDC 16334 (DSMZ 6172), *Malassezia globosa* ATCC MYA 4612, and *Malassezia furfur* ATCC 14521. All the fungal strains were stored at −80 °C in cryovials. Then *Candida* spp. and *Malassezia pachydermatis* were reactivated in RPMI 1640 broth for 24–48 h at 35 °C. The so obtained fresh cultures were streaked onto Sabouraud dextrose agar and incubated for 24–48 h at 35 °C. *Malassezia globosa* and *furfur* were reactivated in modified RPMI 1640 broth^32^ for 24–48 h at 35 °C (*M. furfur*) and 33 °C (*M. globosa*), and then the cultures were streaked onto Leeming and Notman agar (MLNA) and incubated for 48–72 h at 35 °C for *M. furfur* and at 33 °C for *M. globosa*. The fungal inoculum for the testing was prepared by suspending in phosphate buffer (PB) 10 mM pH 7 4–5 colonies of about 1 mm diameter. The fungal suspension was then adjusted to a final optical density of 0.5 McFarland standard (1–5 × 10^6^ cells/mL). The compound solutions were tested in a range of concentrations from 0.01 μg/mL to 256 μg/mL, and 50 μL were then added in 96 wells microtiter plates with 50 μL of fungal inoculum and incubated for 24–48 h at 35 °C. Growth and sterility controls were performed. After 24–48 h of incubation at 35 °C for *Candida* spp. and *M. pachydermatis*, and 48–72 h at 33 °C for *M. globosa* and 35 °C for *M. furfur*, MIC reading was performed.

### Cytotoxicity Test

Compounds cytotoxicity was evaluated on MDBK and HaCat cells. About 5 × 10^5^ cells/mL were incubated overnight at 37 °C in DMEM medium in a humidified atmosphere with 5% CO_2_ in the presence of 0.5–256 μg/mL of compounds. Negative controls were performed in absence of compounds and positive controls in the presence of 0.2% Triton X-100. After incubation, 10 μL of MTT (3-(4,5-dimethylthiazol-2-yl)-2,5-diphenyltetrazolium bromide) was added to each well and incubated at 37 °C for 6 h. At the end of the incubation, 100 μL of the solubilization solution (10% SDS in 0.01 M HCl) was added to each well and then incubated overnight. The yellow tetrazolium MTT salt is reduced in metabolically active cells to form insoluble purple formazan crystals, which are solubilized by the addition of a detergent. After incubation, plates were read with a spectrophotometer (620 nm).^33^

### Hemolysis Test

Sheep defibrinated blood was centrifuged at 100*g* for 15 min and washed three times with PBS, centrifuged at 1000*g* for 10 min, and resuspended to concentration of 2% (*v*/*v*) in PB containing 308 mM sucrose to maintain cell osmolarity. Fifty microliters of RBCs was incubated with 50 μL of different compound concentrations for 1 h at room temperature. The suspension was then centrifuged at 1000*g* for 5 min and hemolysis measured at 450 nm. Negative controls (0% hemoglobin release), obtained in absence of compounds, and positive controls (100% hemoglobin release), obtained in the presence of 1% Tween 20, were carried out. Hemolysis percentage was calculated as follows:
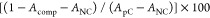
where *A*_comp_ represented the optical density of sample at 450 nm, *A*_pC_ the optical density of positive control, and *A*_NC_ the optical density of negative control. A hemolysis percentage less than or equal to 10% was considered acceptable.^34^

## Supporting Information Available

^1^H, ^13^C, ^19^F and ^77^Se NMR spectra of compounds **7a**–**k**, **8a**–**g**, **9a**–**f**, **10a**–**g**, **11a**–**j**, **12**; SD% values for compounds **3a**, **3c**, **5b**, **7a**–**d**, **9c**, **11e**, and **11i**; *P*-values calculated with *t* test of data in Figures 1–3 (PDF)

## Abbreviations

CA: carbonic anhydrase
CAI: carbonic anhydrase inhibitors
MIC: minimal inhibitory concentration

## References

[ref1] Fones H. N.; Fisher M. C.; Gurr S. J. Emerging fungal threats to plants and animals challenge agriculture and ecosystem resilience. Microbiol. Spectr. 2017, 5, 2–16. 10.1128/microbiolspec.FUNK-0027-2016. PMC11687465 28361733

[ref2] Brown G. D.; Denning D. W.; Gow N. A.; Levitz S. M.; Netea M. G.; White T. C. Hidden killers: human fungal infections. Sci. Transl. Med. 2012, 4, 165rv13 10.1126/scitranslmed.3004404. 23253612

[ref3] Fisher M. C.; Hawkins N. J.; Sanglard D.; Gurr S. J. Worldwide emergence of resistance to antifungal drugs challenges human health and food security. Science. 2018, 360, 739–742. 10.1126/science.aap7999. 29773744

[ref4] Invitation to participate in survey to establish the first WHO fungal priority pathogens list; WHO, 2021. https://www.who.int/news-room/articles-detail/invitation-to-participate-in-survey-to-establish-the-first-who-fungal-priority-pathogens-list-(fppl) (accessed 03-05-2022).

[ref5] Rhimi W.; Theelen B.; Boekhout T.; Otranto D.; Cafarchia C. Malassezia spp. yeasts of emerging concern in fungemia. Front. Cell. Infect. Microbiol. 2020, 10, 370 10.3389/fcimb.2020.00370. 32850475 PMC7399178

[ref6] Morris D. O.; O’Shea K.; Shofer F. S.; Rankin S. Malassezia pachydermatis carriage in dog owners. Emerg. Infect. Dis. 2005, 11, 83–88. 10.3201/eid1101.040882. 15705327 PMC3294355

[ref7] Figueredo L. A.; Cafarchia C.; Desantis S.; Otranto D. Biofilm formation of Malassezia pachydermatis from dogs. Vet. Microbiol. 2012, 160, 126–131. 10.1016/j.vetmic.2012.05.012. 22682201

[ref8] Lepesheva G. I.; Waterman M. R. Sterol 14alpha-demethylase cytochrome P450 (CYP51), a P450 in all biological kingdoms. Biochim. Biophys. Acta 2007, 3, 467–477. 10.1016/j.bbagen.2006.07.018. PMC2324071 16963187

[ref9] Wu Z. L.; Yin X. B.; Lin Z. Q.; Bañuelos G. S.; Yuan L. X.; Liu Y.; Li M. Inhibitory effect of selenium against penicillium expansum and its possible mechanisms of action. Curr. Microbiol. 2014, 69, 192–201. 10.1007/s00284-014-0573-0. 24682262

[ref10] Aggarwal K.; Jain V.; Sangwan S. Comparative study of ketoconazole versus selenium sulphide shampoo in pityriasis versicolor. Indian J. Dermatol. Venereol. Leprol. 2003, 69, 86. 17642841

[ref11] McGinley K. J.; Leyden J. J. Antifungal activity of dermatological shampoos. Arch. Dermatol. Res. 1982, 272, 339–342. 10.1007/BF00509065. 7165342

[ref12] Kieliszek M.; Dourou M. Effect of selenium on the growth and lipid accumulation of yarrowia lipolytica yeast. Biol. Trace Elem. Res. 2021, 4, 1611–1622. 10.1007/s12011-020-02266-w. PMC7886723 32632749

[ref13] Hu X.; Chandler J. D.; Orr M. L.; Hao L.; Liu K.; Uppal K.; Go Y.-M.; Jones D. P. Selenium supplementation alters hepatic energy and fatty acid metabolism in mice. J. Nutr. 2018, 5, 675–684. 10.1093/jn/nxy036. PMC6454983 29982657

[ref14] Kieliszek M.; Błazejak S.; Bzducha-Wróbel A.; Kot A.-M. Effect of selenium on lipid and amino acid metabolism in yeast cells. Biol. Trace Elem. Res. 2019, 187, 316–327. 10.1007/s12011-018-1342-x. 29675568 PMC6315055

[ref15] Chen Z.; Lai H.; Hou L.; Chen T. Rational design and action mechanisms of chemically innovative organoselenium in cancer therapy. Chem. Commun. (Camb). 2019, 2, 179–196. 10.1039/C9CC07683B. 31782422

[ref16] Supuran C. T.; Capasso C. A Highlight on the inhibition of fungal carbonic anhydrases as drug targets for the antifungal armamentarium. Int. J. Mol. Sci. 2021, 22, 4324 10.3390/ijms22094324. 33919261 PMC8122340

[ref17] Supuran C. T. Emerging role of carbonic anhydrase inhibitors. Clin Sci. (Lond). 2021, 135, 1233–1249. 10.1042/CS20210040. 34013961

[ref18] Santhosh L.; Kumar L. R.; Sureshbabu V. V. Synthesis of carbodiimides from 1,3-disubstituted selenoureas by iodine-mediated deselenization. Indian J. Chem. 2018, 58, 1291–1294.

[ref19] Angeli A.; Peat T. S.; Bartolucci G.; Nocentini A.; Supuran C. T.; Carta F. Intramolecular oxidative deselenization of acylselenoureas: a facile synthesis of benzoxazole amides and carbonic anhydrase inhibitors. Org. Biomol. Chem. 2016, 14, 11353–11356. 10.1039/C6OB02299E. 27892589

[ref20] Angeli A.; Carta F.; Bartolucci G.; Supuran C. T. Synthesis of novel acyl selenoureido benzensulfonamides as carbonic anhydrase I, II, VII and IX inhibitors. Bioorg. Med. Chem. 2017, 25, 3567–3573. 10.1016/j.bmc.2017.05.014. 28522267

[ref21] Angeli A.; Abbas G.; Del Prete S.; Carta F.; Capasso C.; Supuran C. T. Acyl selenoureido benzensulfonamides show potent inhibitory activity against carbonic anhydrases from the pathogenic bacterium vibrio cholerae. Bioorg. Chem. 2017, 75, 170–172. 10.1016/j.bioorg.2017.09.016. 28957751

[ref22] Angeli A.; Tanini D.; Peat T. S.; Di Cesare Mannelli L.; Bartolucci G.; Capperucci A.; Ghelardini C.; Supuran C. T.; Carta F. Discovery of new selenoureido analogues of 4-(4-fluorophenylureido)benzenesulfonamide as carbonic anhydrase inhibitors. ACS Med. Chem. Lett. 2017, 8, 963–968. 10.1021/acsmedchemlett.7b00280. 28947945 PMC5601372

[ref23] Angeli A.; Ferraroni M.; Da’dara A. A.; Selleri S.; Pinteala M.; Carta F.; Skelly P. J.; Supuran C. T. Structural insights into schistosoma mansoni carbonic anhydrase (SmCA) inhibition by selenoureido-substituted benzenesulfonamides. J. Med. Chem. 2021, 64, 10418–10428. 10.1021/acs.jmedchem.1c00840. 34232641

[ref24] Angiolella L.; Carradori S.; Maccallini C.; Giusiano G.; Supuran C. T. Targeting Malassezia species for novel synthetic and natural antidandruff agents. Curr. Med. Chem. 2017, 22, 2392–2412. 10.2174/0929867324666170404110631. 28393697

[ref25] Koketsu M.; Yamamura Y.; Aoki H.; Ishihara H. The preparation of acylselenourea and selenocarbamate using isoselenocyanate. Phosphorus, Sulfur Silicon Relat. Elem. 2006, 181, 2699–2708. 10.1080/10426500600862894.

[ref26] Khalifah R. G. The carbon dioxide hydration activity of carbonic anhydrase. I., Stop flow kinetic studies on the native human isoenzymes B and C. J. Biol. Chem. 1971, 246, 2561 10.1016/S0021-9258(18)62326-9. 4994926

[ref27] Karioti A.; Carta F.; Supuran C. T. Phenols and polyphenols as carbonic anhydrase inhibitors. Molecules 2016, 12, 1649 10.3390/molecules21121649. PMC6273245 27918439

[ref28] Vullo D.; Durante M.; Di Leva F. S.; Cosconati S.; Masini E.; Scozzafava A.; Novellino E.; Supuran C. T.; Carta F. Monothiocarbamates strongly inhibit carbonic anhydrases in vitro and possess intraocular pressure lowering activity in an animal model of glaucoma. J. Med. Chem. 2016, 12, 5857–5867. 10.1021/acs.jmedchem.6b00462. 27253845

[ref29] Vannozzi G.; Vullo D.; Angeli A.; Ferraroni M.; Combs J.; Lomelino C.; Andring J.; Mckenna R.; Bartolucci G.; Pallecchi M.; Lucarini L.; Sgambellone S.; Masini E.; Carta F.; Supuran C. T. One-Pot procedure for the synthesis of asymmetric substituted ureido benzene sulfonamides as effective inhibitors of carbonic anhydrase enzymes. J. Med. Chem. 2022, 65, 824–837. 10.1021/acs.jmedchem.1c01906. 34958217

[ref30] De Luca V.; Angeli A.; Mazzone V.; Adelfio C.; Carginale V.; Scaloni A.; Carta F.; Selleri S.; Supuran C. T.; Capasso C. Heterologous expression and biochemical characterisation of the recombinant β-carbonic anhydrase (MpaCA) from the warm-blooded vertebrate pathogen malassezia pachydermatis. J. Enzyme Inhib. Med. Chem. 2022, 37, 62–68. 10.1080/14756366.2021.1994559. 34894958 PMC8667878

[ref31] CLSI. Reference Method for Broth Dilution Antifungal Susceptibility Testing of Yeasts, 4th ed.; Clinical and Laboratory Standard Institute: Wayne, PA, 2017.

[ref32] Rojas F. D.; Sosa M. D. L. A.; Fernandez M. S.; Cattana M. E.; Cordoba S. B.; Giusiano G. E. Antifungal susceptibility of malassezia furfur, malassezia sympodialis, and malassezia globosa to azole drugs and amphotericin B evaluated using a broth microdilution method. Sabouraudia 2014, 6, 641–646. 10.1093/mmy/myu010. 24965946

[ref33] Donofrio G.; Franceschi V.; Capocefalo A.; Cavirani S.; Sheldon I. M. Bovine endometrial stromal cells display osteogenic properties. Reprod. Biol. Endocrinol. 2008, 6, 65 10.1186/1477-7827-6-65. 19087287 PMC2657796

[ref34] Amin K.; Dannenfelser R. M. In vitro hemolysis: guidance for the pharmaceutical scientist. J. Pharm. Sci. 2006, 6, 1173–1176. 10.1002/jps.20627. 16639718

