# Supplementary material for: Seleno Containing Compounds as Potent and Selective Antifungal Agents
Source: ACS Infect Dis. 2022 Aug 19;8(9):1905–19. doi: 10.1021/acsinfecdis.2c00250 (PMC9940851; doi:10.1021/acsinfecdis.2c00250)
Supplement: Supplementary file 1 — id2c00250_si_001.pdf [file id2c00250_si_001.pdf]

Supplementary Material for

Seleno Containing Compounds as Potent and Selective Antifungal Agents

Andrea Angeli[a†], Alice Velluzzi[a†], Silvia Selleri[a], Clemente Capasso[b], Costanza Spadini[c], Mattia Iannarelli[c], Clotilde S. Cabassi[c*], Fabrizio Carta[a*] and Claudiu T. Supuran[a]

[a] NEUROFARBA Department, Sezione di Scienze Farmaceutiche e Nutraceutiche, University of Florence, Via Ugo Schiff 6, 50019 Sesto Fiorentino, Florence, Italy.

[b] Department of Biology, Agriculture and Food Sciences, Institute of Biosciences and Bioresources, 80131, Napoli, Italy.

[c] Department of Veterinary Science, University of Parma, via del Taglio 10, 43126, Parma, Italy.

[†] These Authors contributed equally

Corresponding Author

***Email:** fabrizio.carta@unifi.it

***Email:** clotildesilvia.cabassi@unipr.it.

Index

^{1}H, ^{13}C, ^{19}F, ^{77}Se Spectra of compounds **7a-k, 8a-g, 9a-f, 10a-g, 11a-j, 12** S2-45

SD % values for compounds **3a**, **3c**, **5b**, **7a-d**, **9c**, **11e** and **11i (Table S1)** S46

P-values calculated with T-test of data on **Figures 1-3** (**Tables S2-S4**) S47-S50

NMR Spectra of synthesized compounds

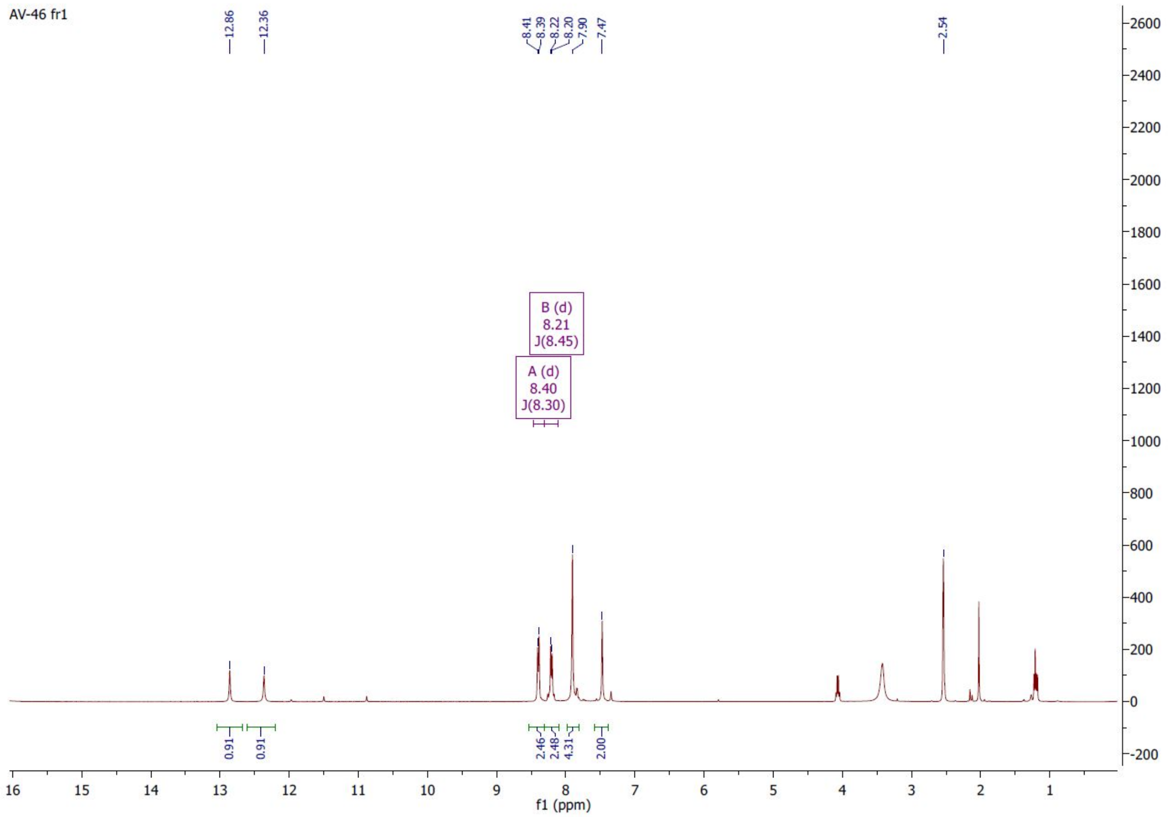

^{1}H NMR spectrum of compound **7f** (400 MHz, DMSO-d_6)

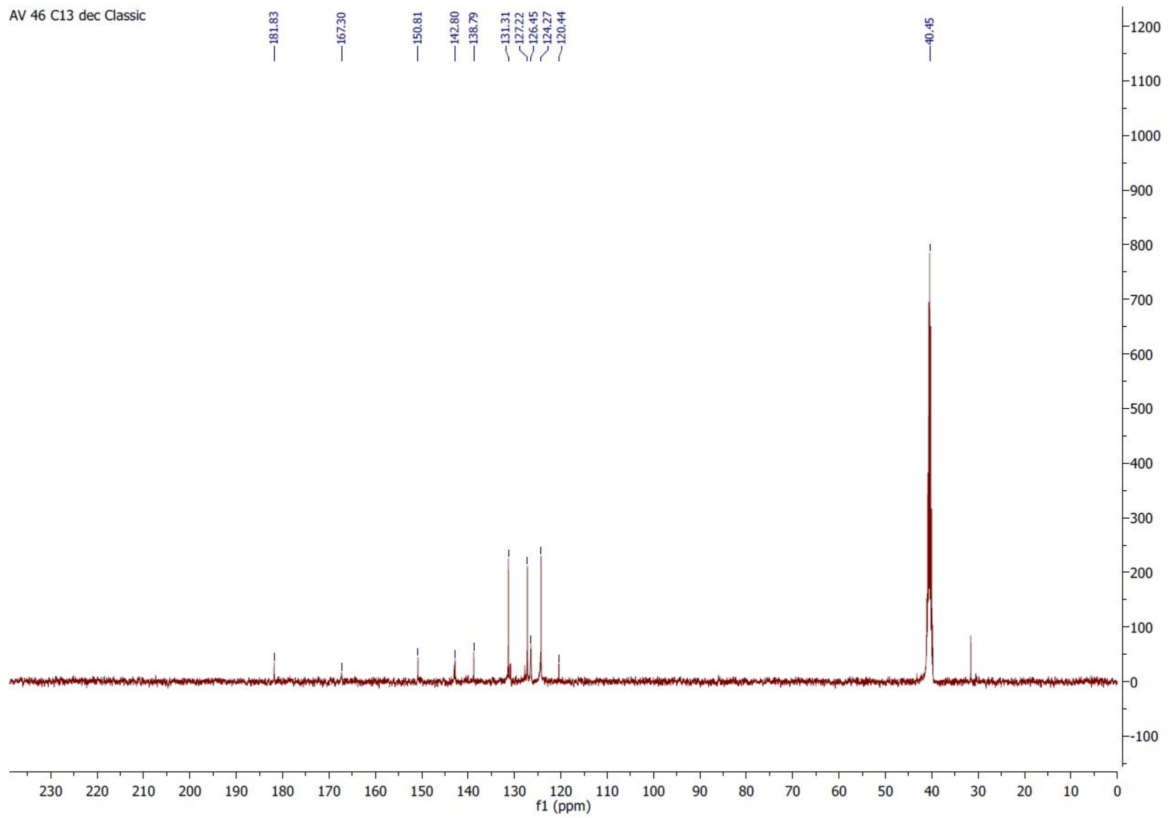

^{13}C NMR spectrum of compound **7f** (100 MHz, DMSO-d_6)

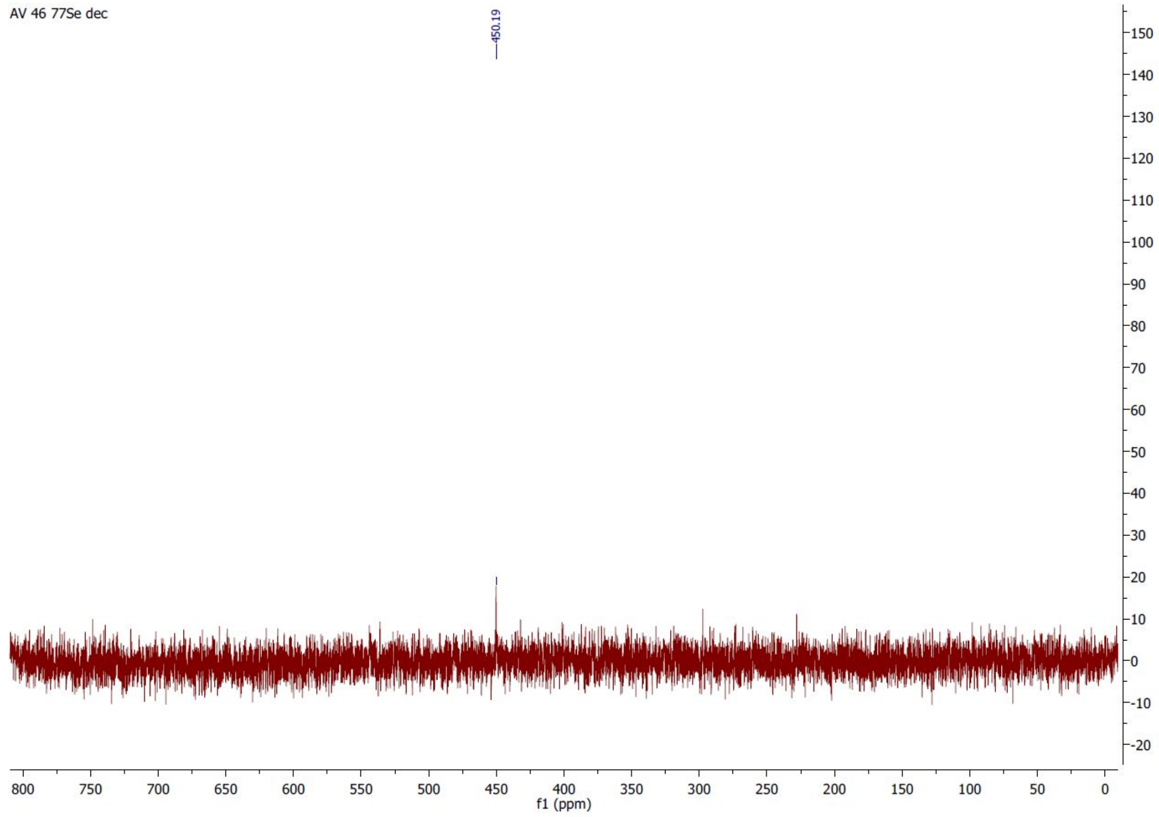

^{77}Se NMR spectrum of compound **7f** (76 MHz, DMSO-d_6)

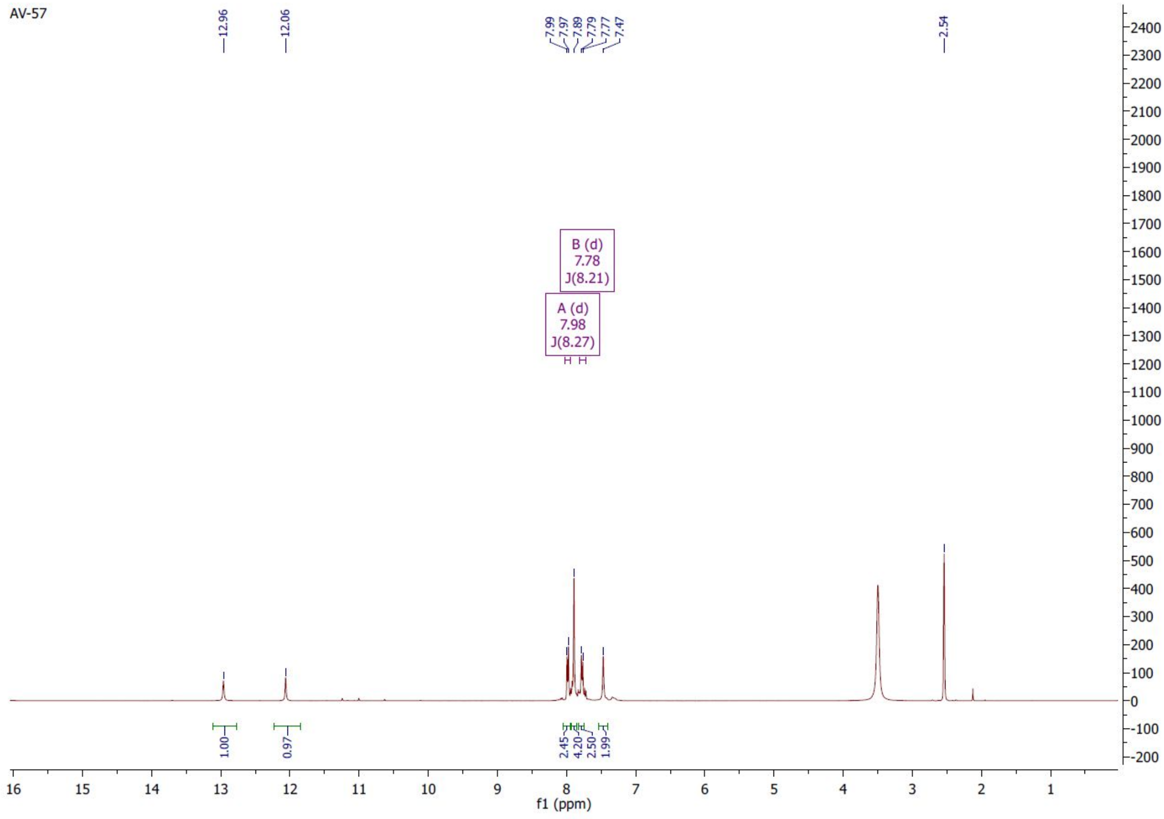

^{1}H NMR spectrum of compound **7g** (400 MHz, DMSO-d_6)

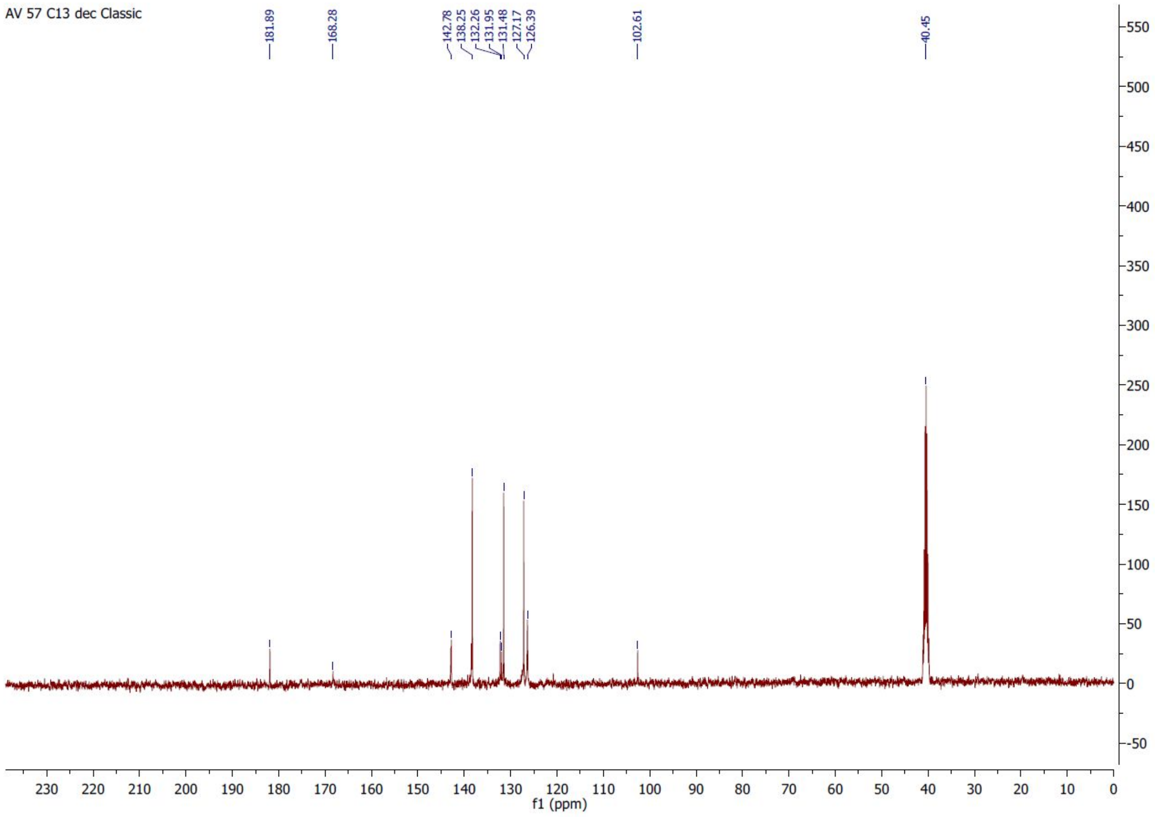

^{13}C NMR spectrum of compound **7g** (100 MHz, DMSO-d_6)

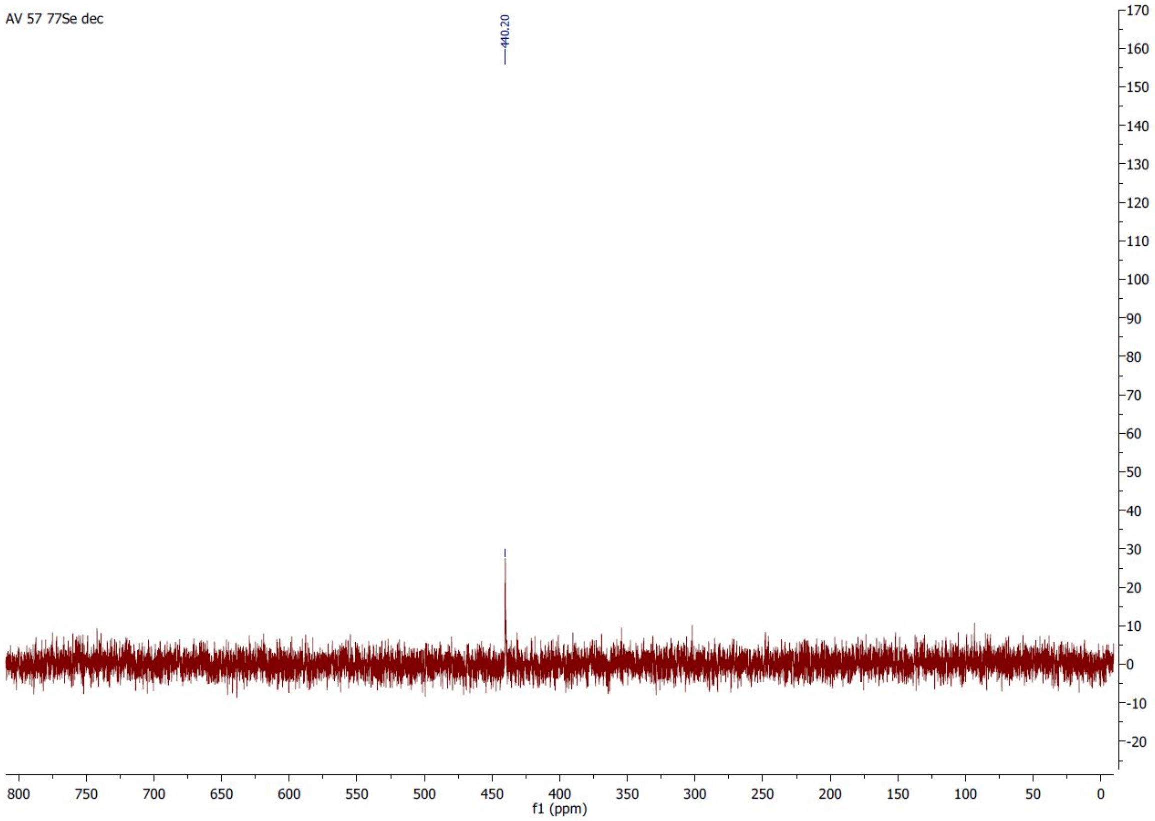

^{77}Se NMR spectrum of compound **7g** (76 MHz, DMSO-d_6)

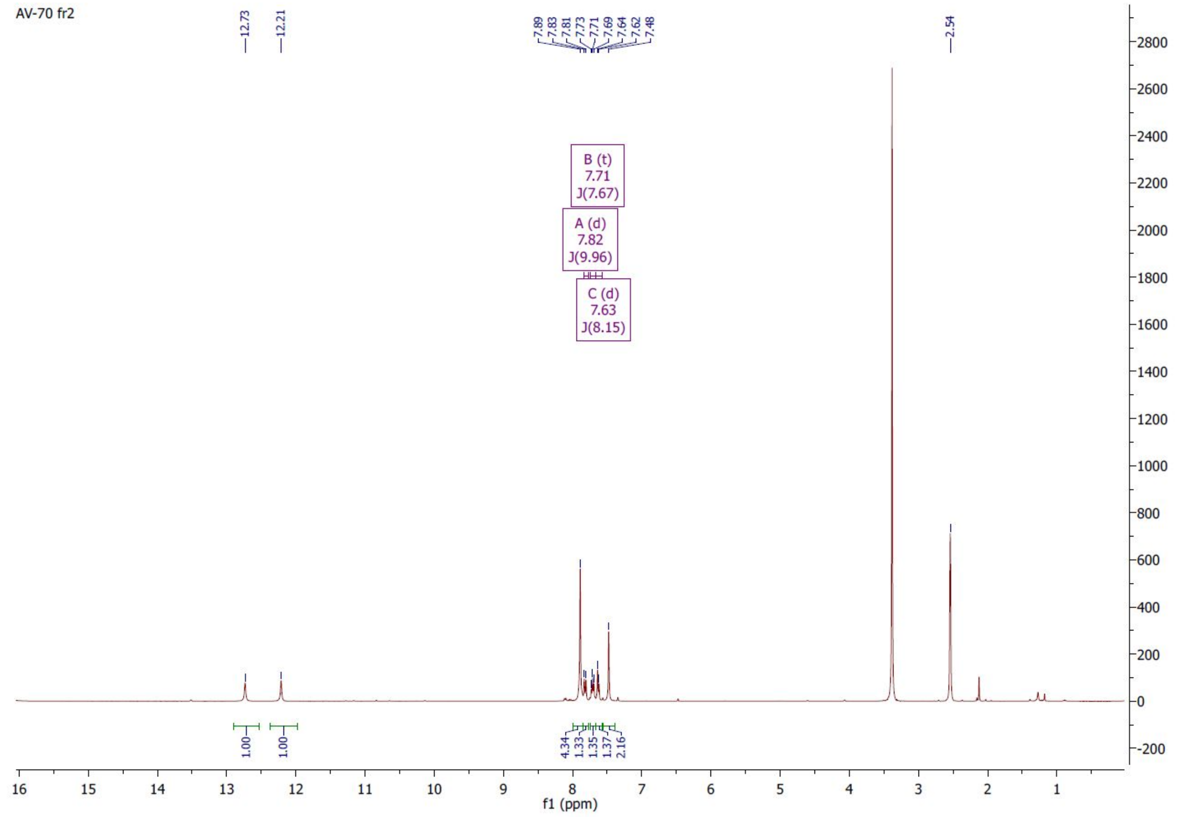

^{1}H NMR spectrum of compound **7h** (400 MHz, DMSO-d_6)

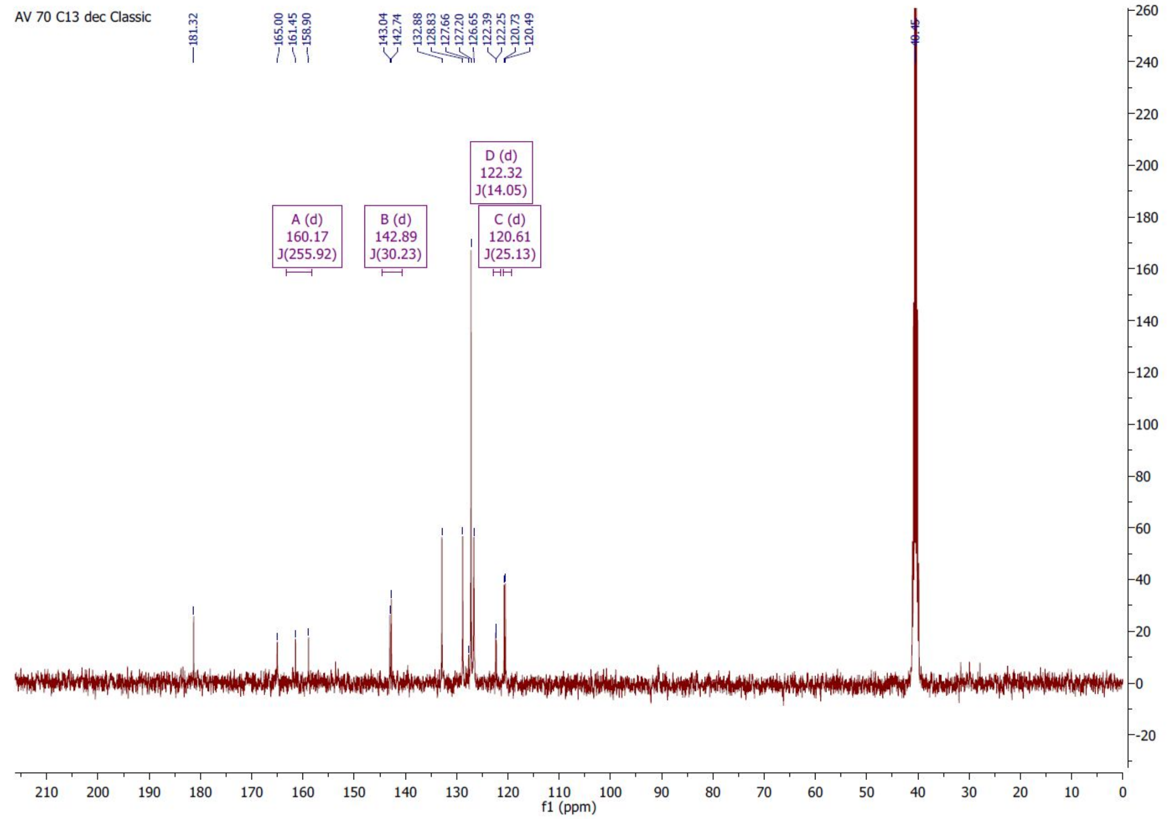

^{13}C NMR spectrum of compound **7h** (100 MHz, DMSO-d_6)

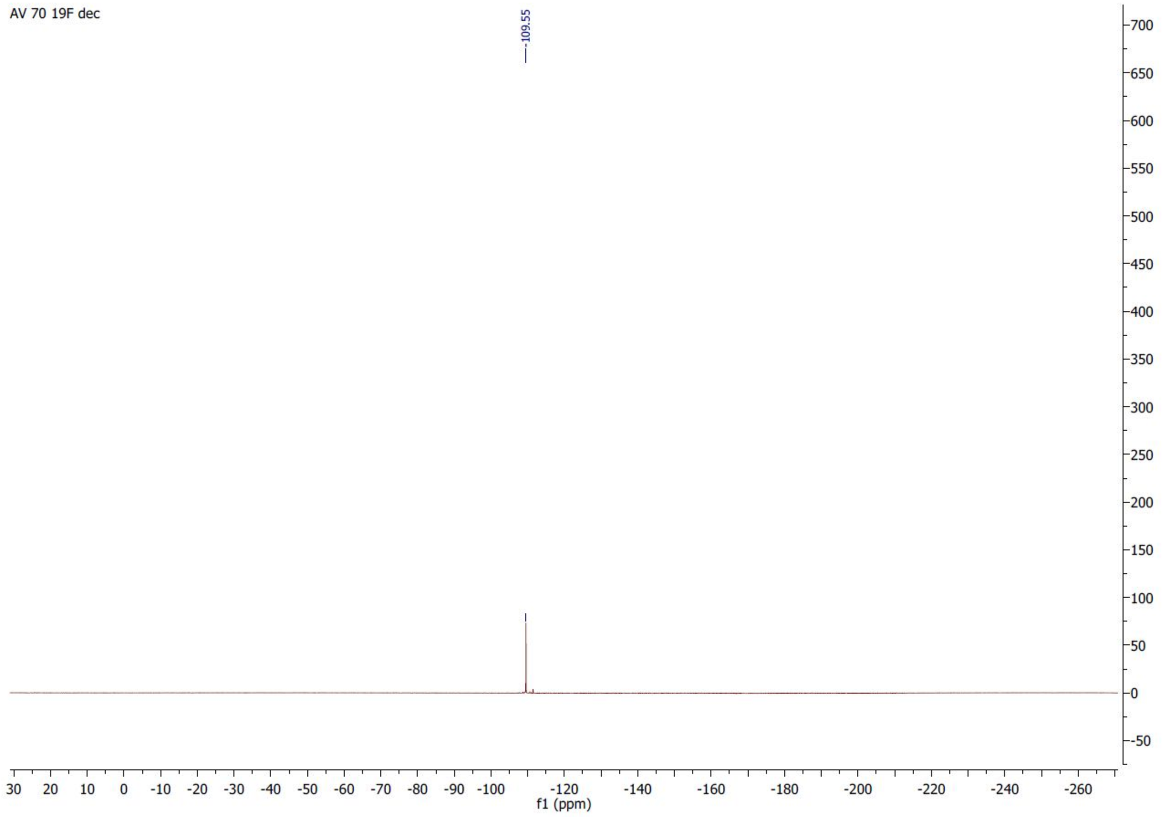

^{19}F NMR spectrum of compound **7h** (376 MHz, DMSO-d_6)

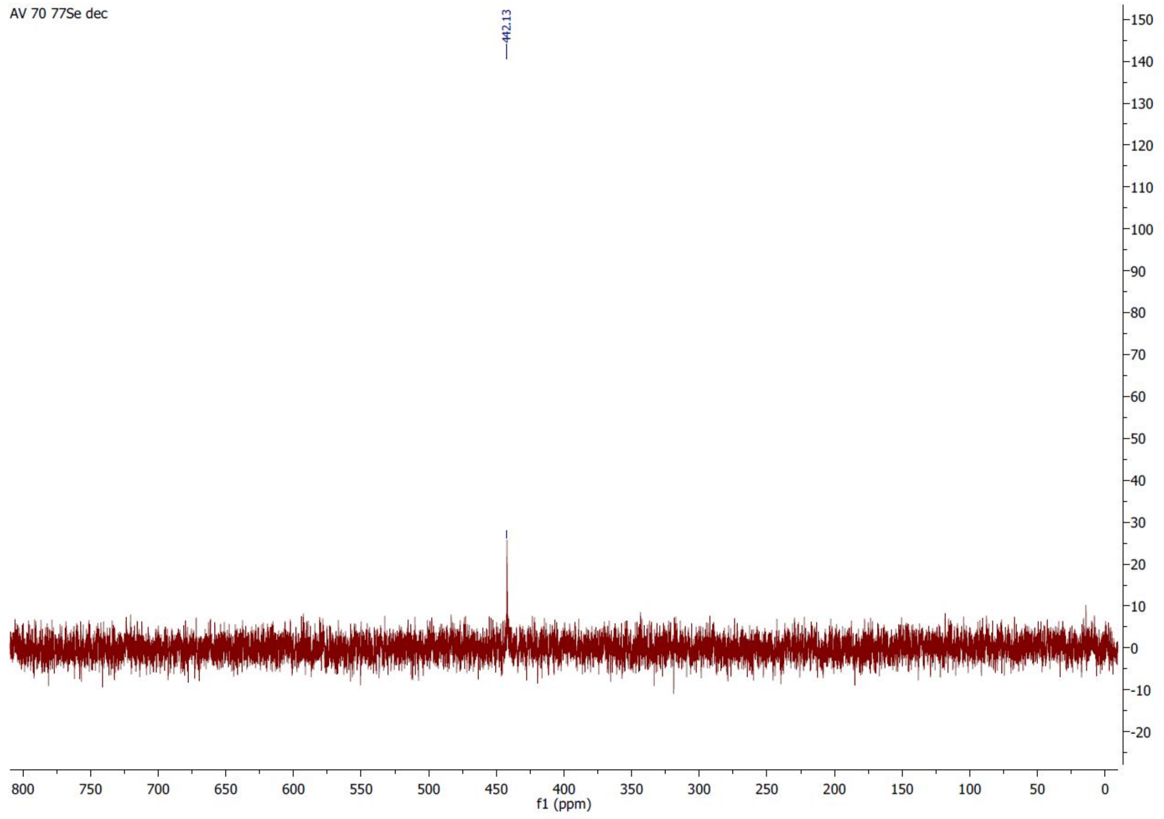

^{77}Se NMR spectrum of compound **7h** (76 MHz, DMSO-d_6)

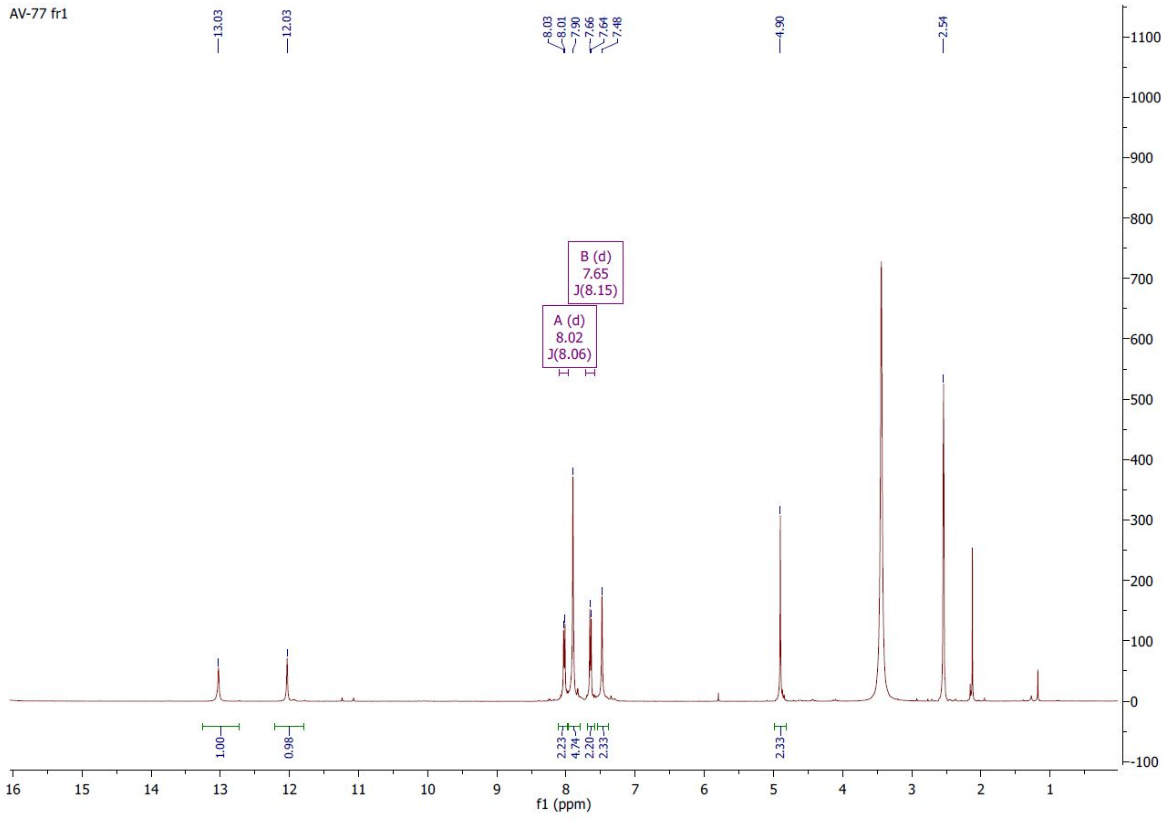

^{1}H NMR spectrum of compound **7i** (400 MHz, DMSO-d_6)

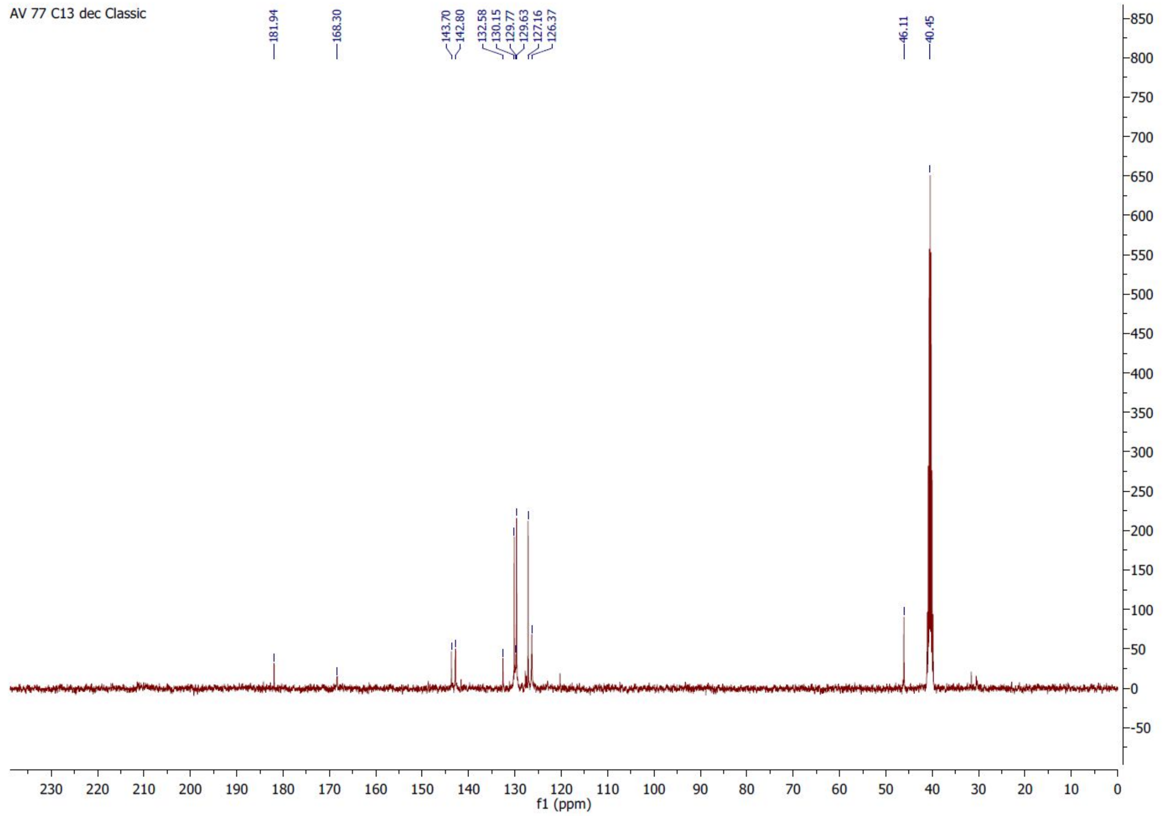

^{13}C NMR spectrum of compound **7i** (100 MHz, DMSO-d_6)

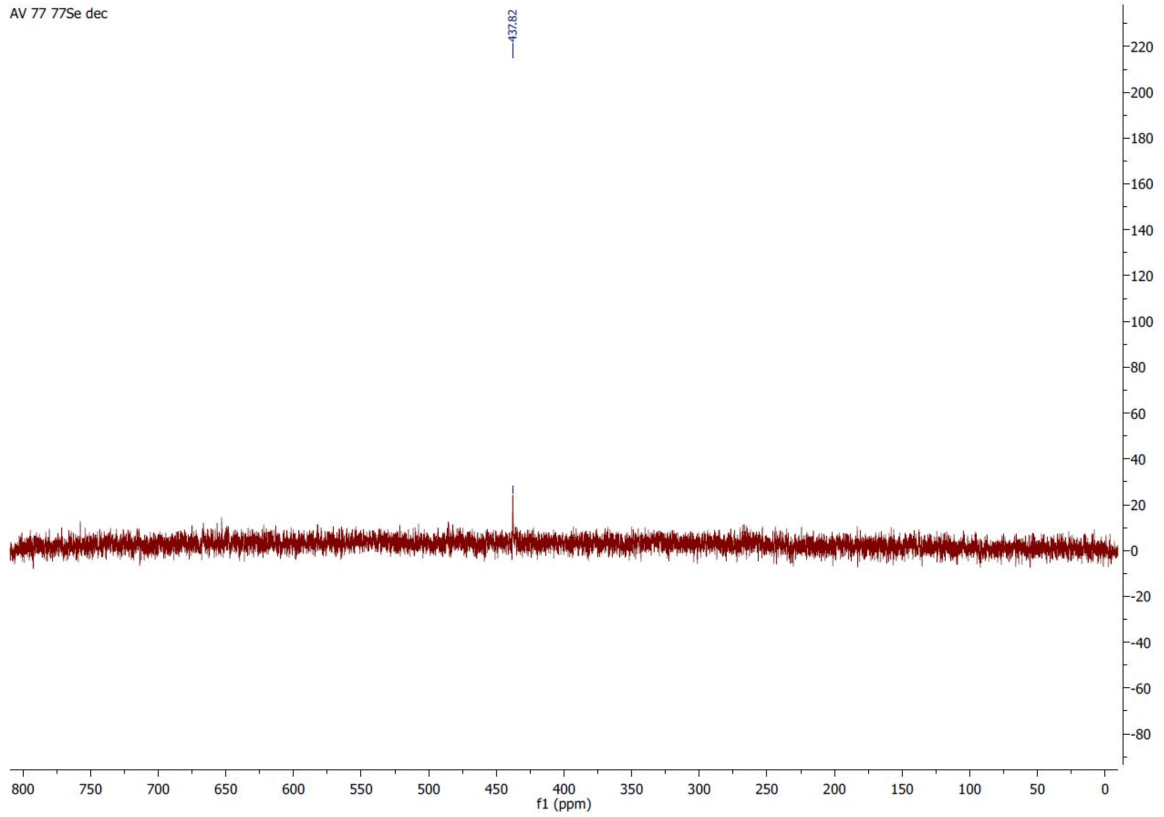

^{77}Se NMR spectrum of compound **7i** (76 MHz, DMSO-d_6)

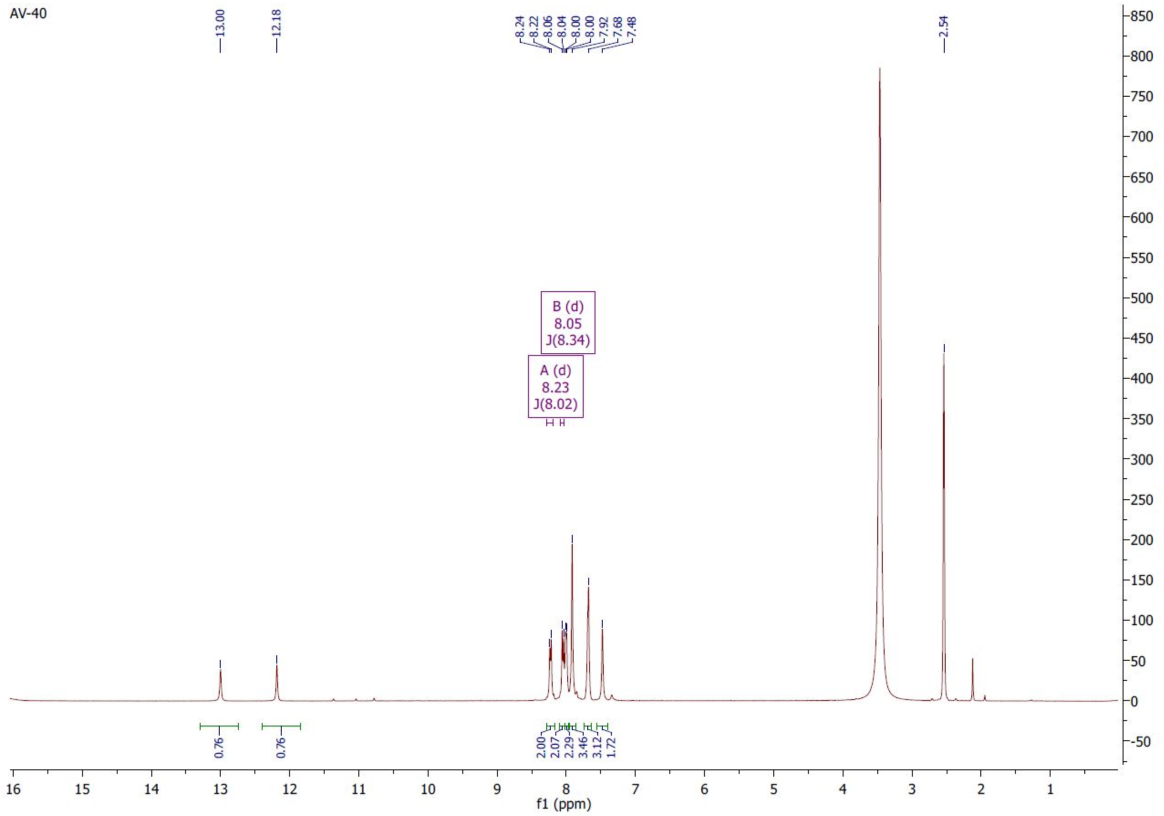

^{1}H NMR spectrum of compound **7j** (400 MHz, DMSO-d_6)

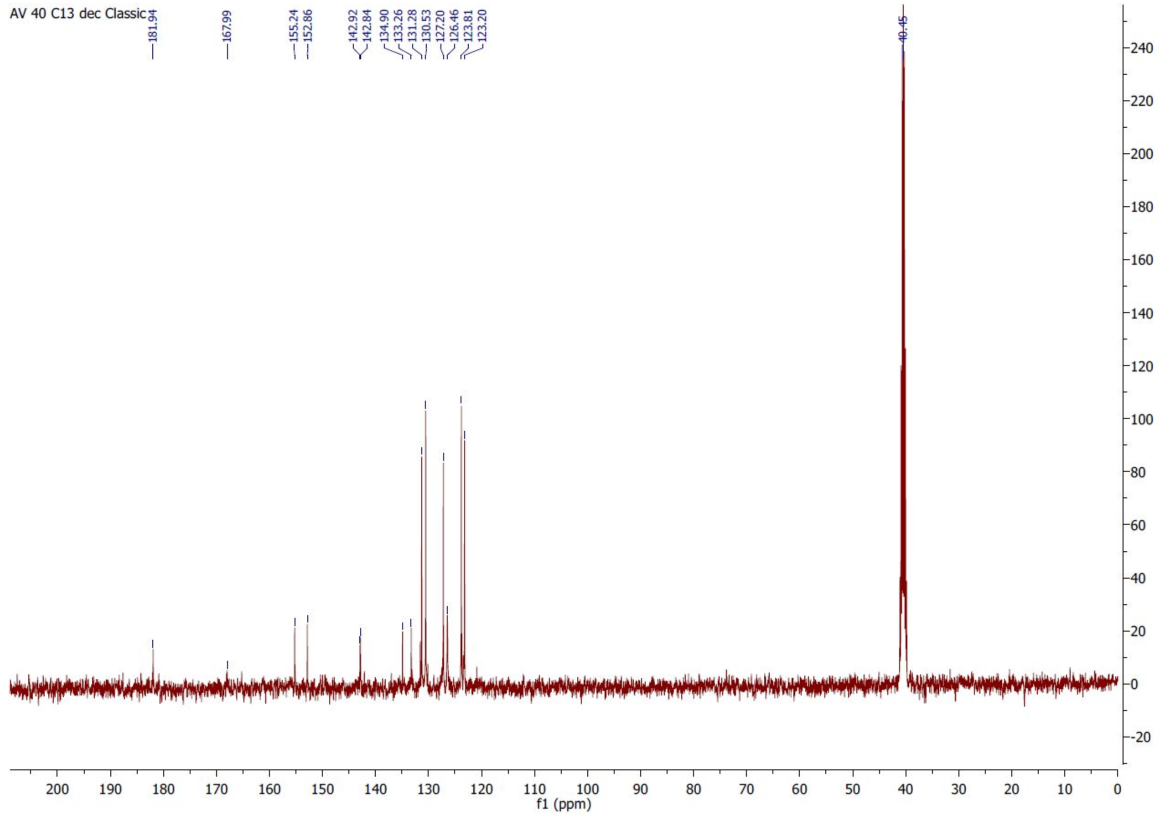

^{13}C NMR spectrum of compound **7j** (100 MHz, DMSO-d_6)

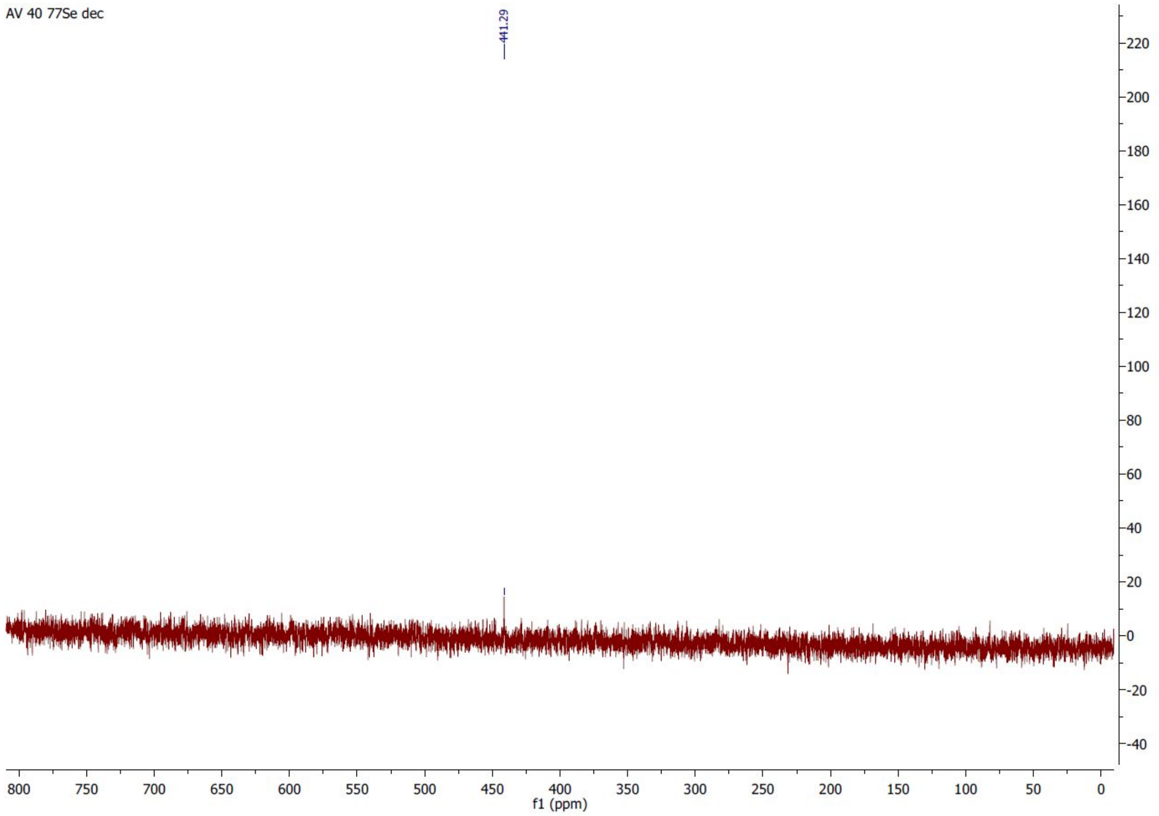

^{77}Se NMR spectrum of compound **7j** (76 MHz, DMSO-d_6)

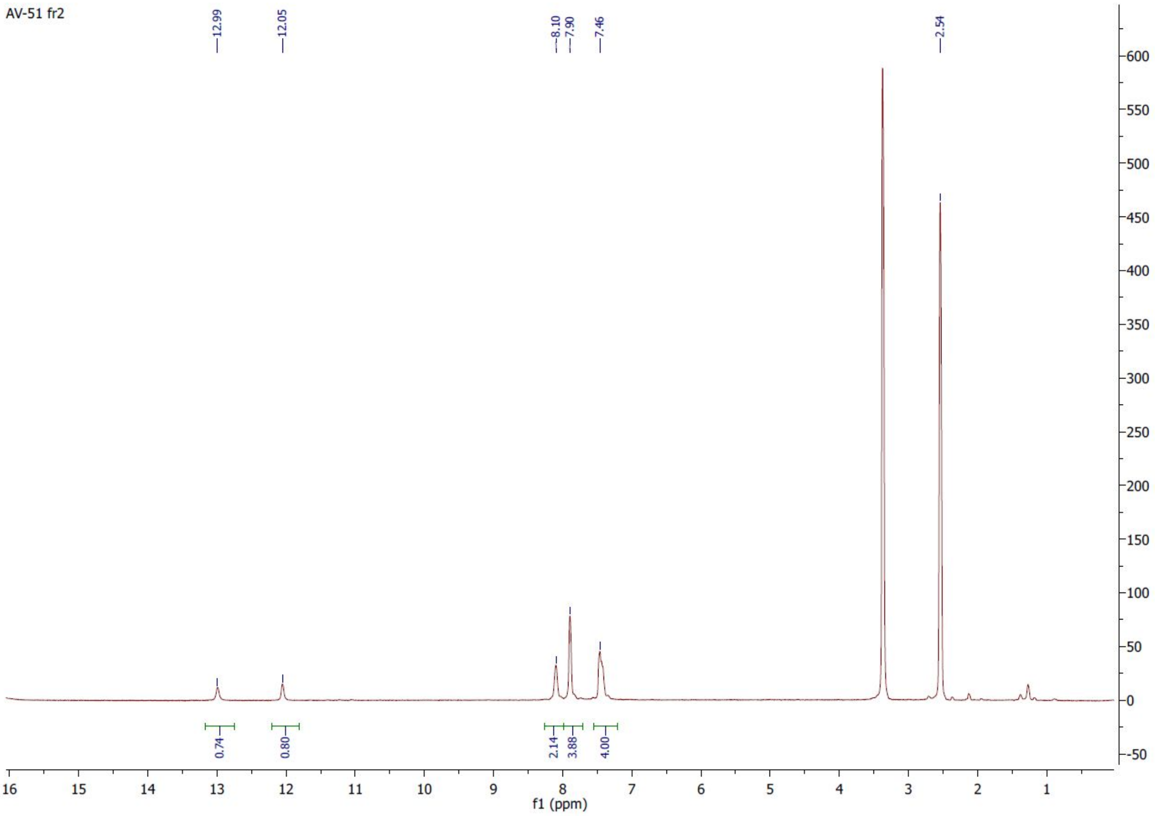

^{1}H NMR spectrum of compound **7k** (400 MHz, DMSO-d_6)

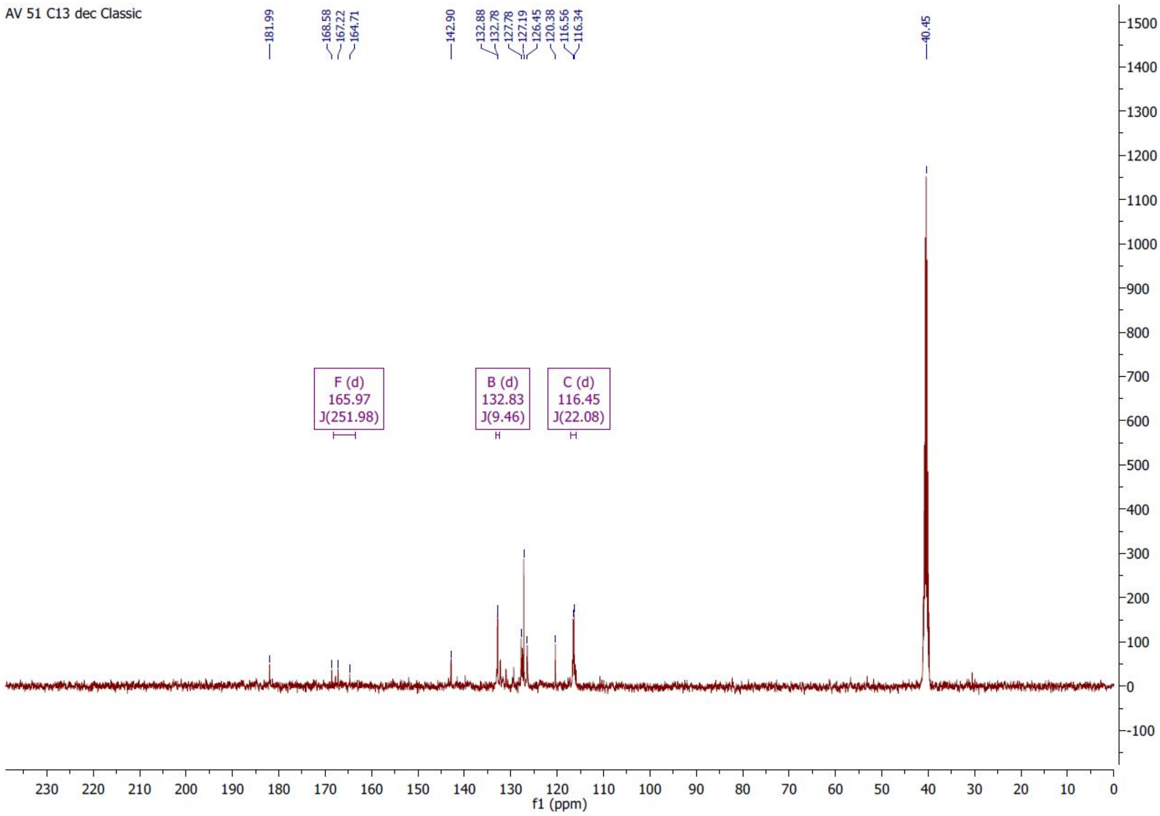

^{13}C NMR spectrum of compound **7k** (100 MHz, DMSO-d_6)

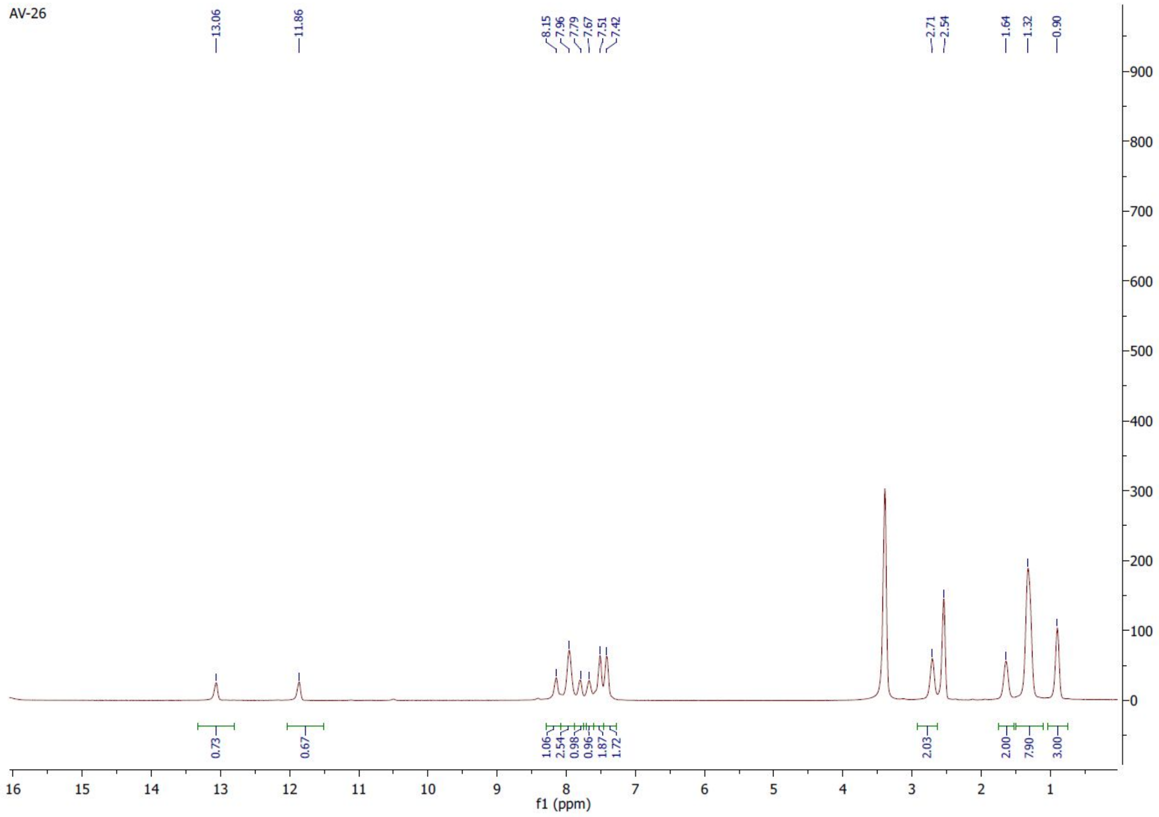

^{1}H NMR spectrum of compound **8b** (400 MHz, DMSO-d_6)

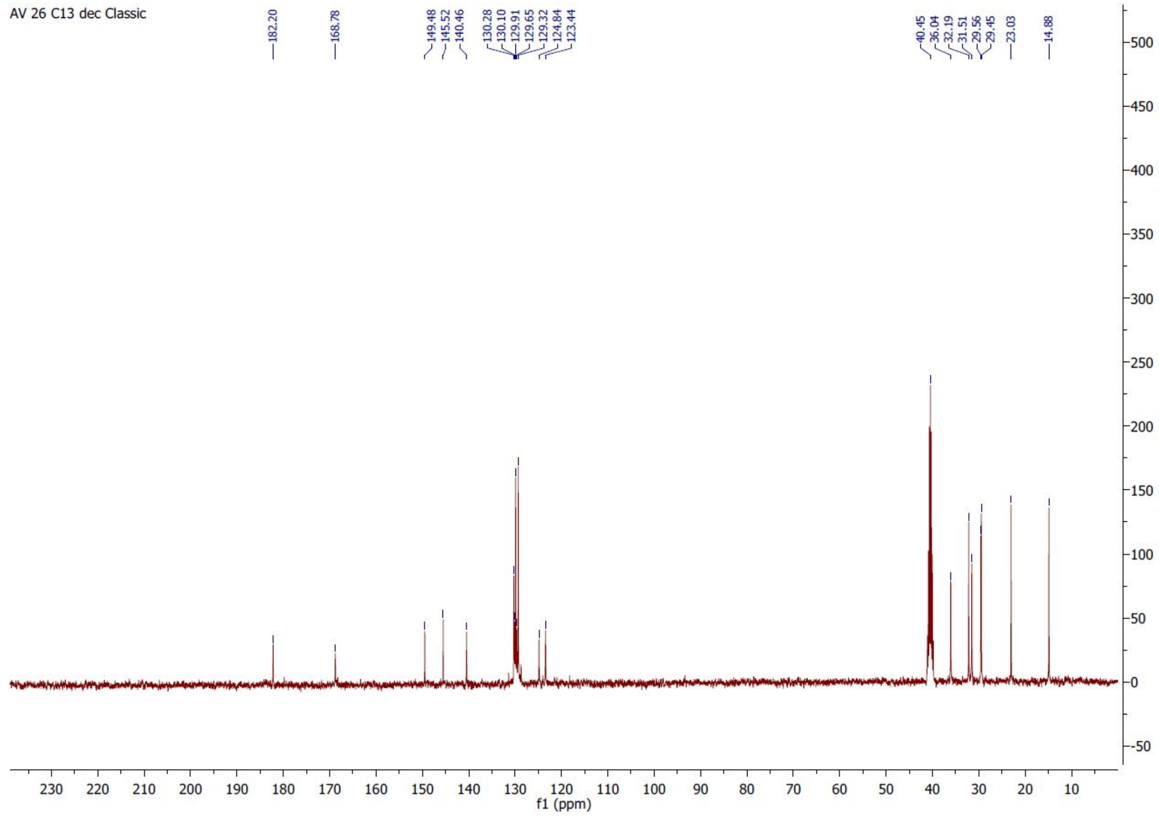

^{13}C NMR spectrum of compound **8b** (100 MHz, DMSO-d_6)

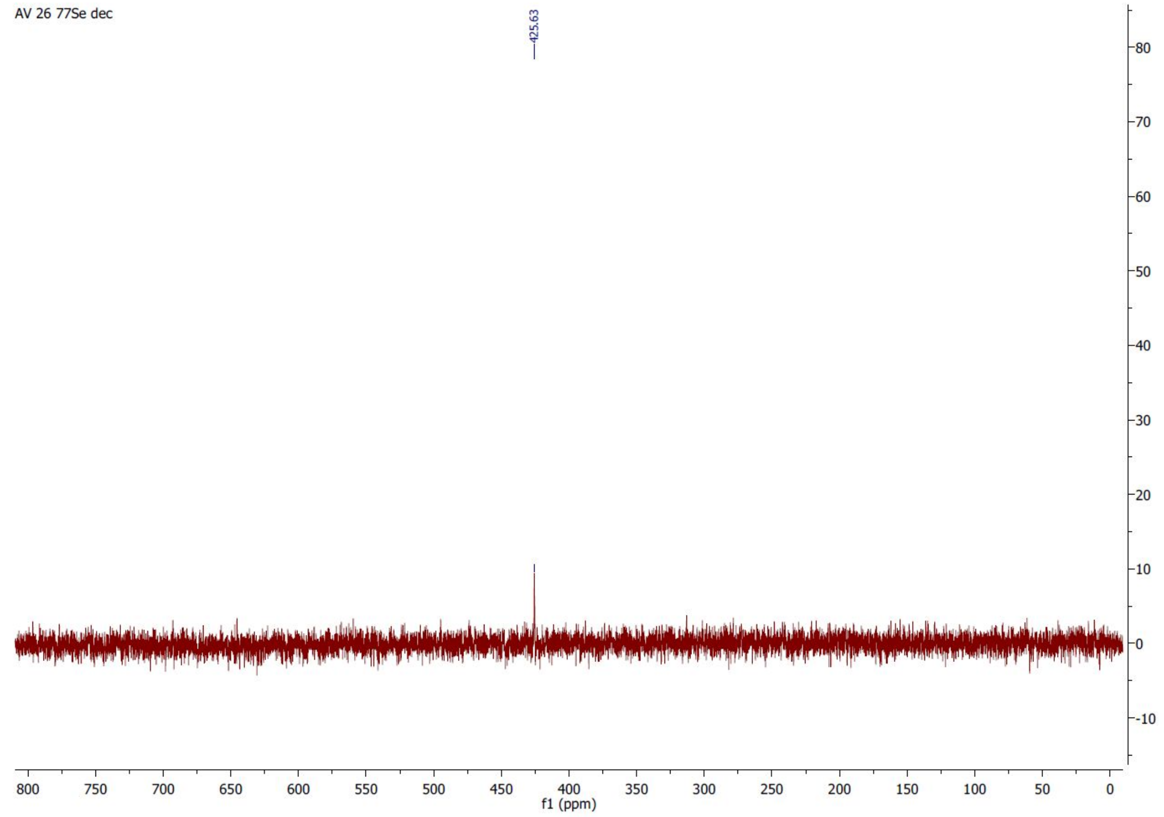

^{77}Se NMR spectrum of compound **8b** (76 MHz, DMSO-d_6)

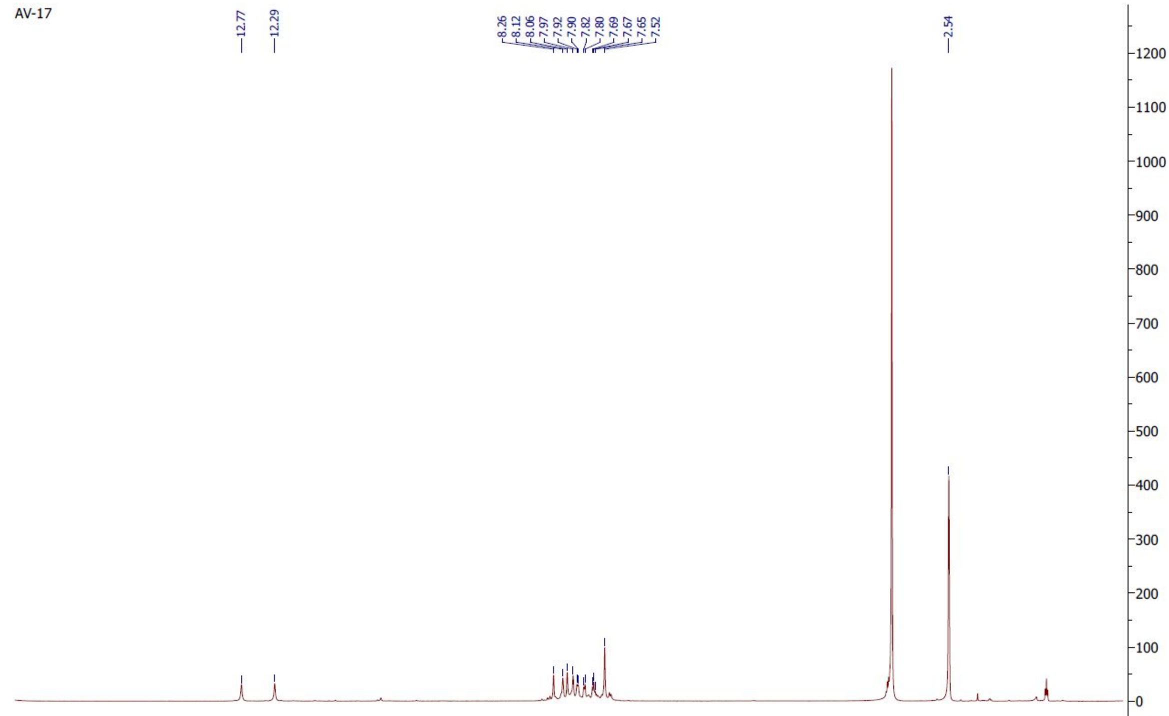

^{1}H NMR spectrum of compound **8c** (400 MHz, DMSO-d_6)

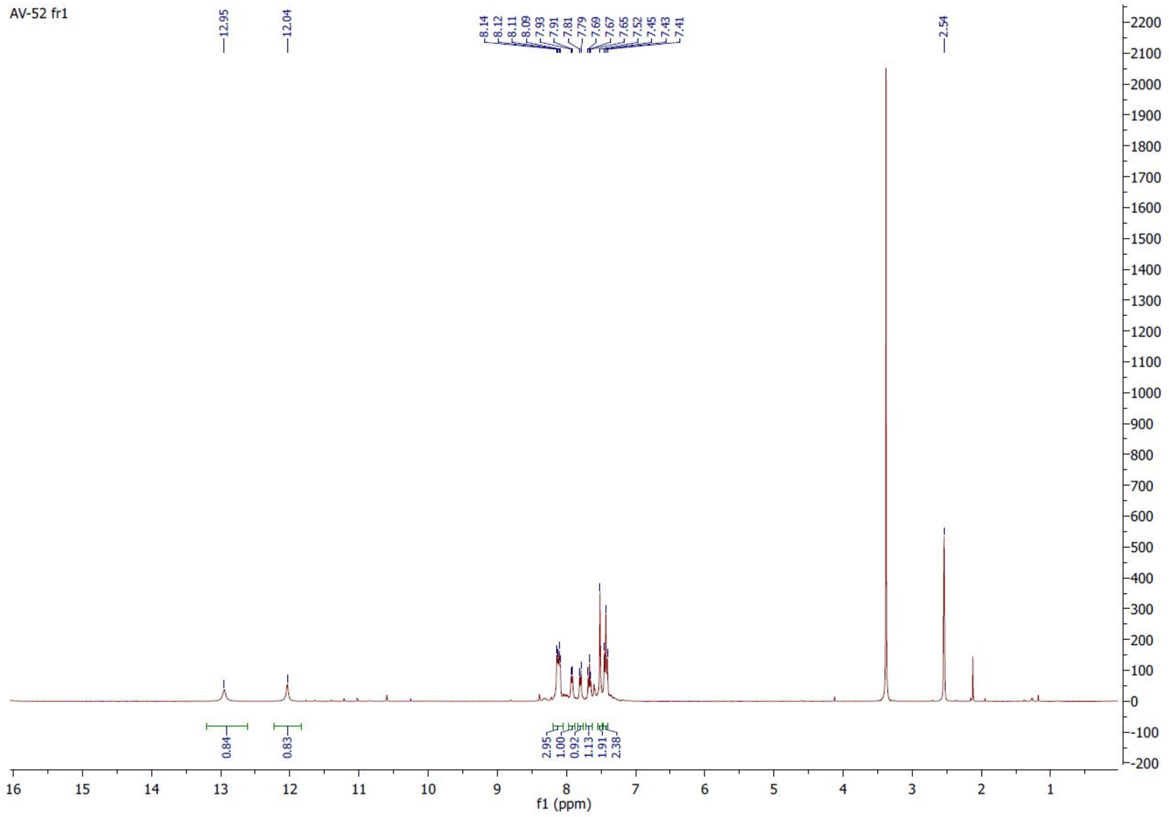

^{1}H NMR spectrum of compound **8d** (400 MHz, DMSO-d_6)

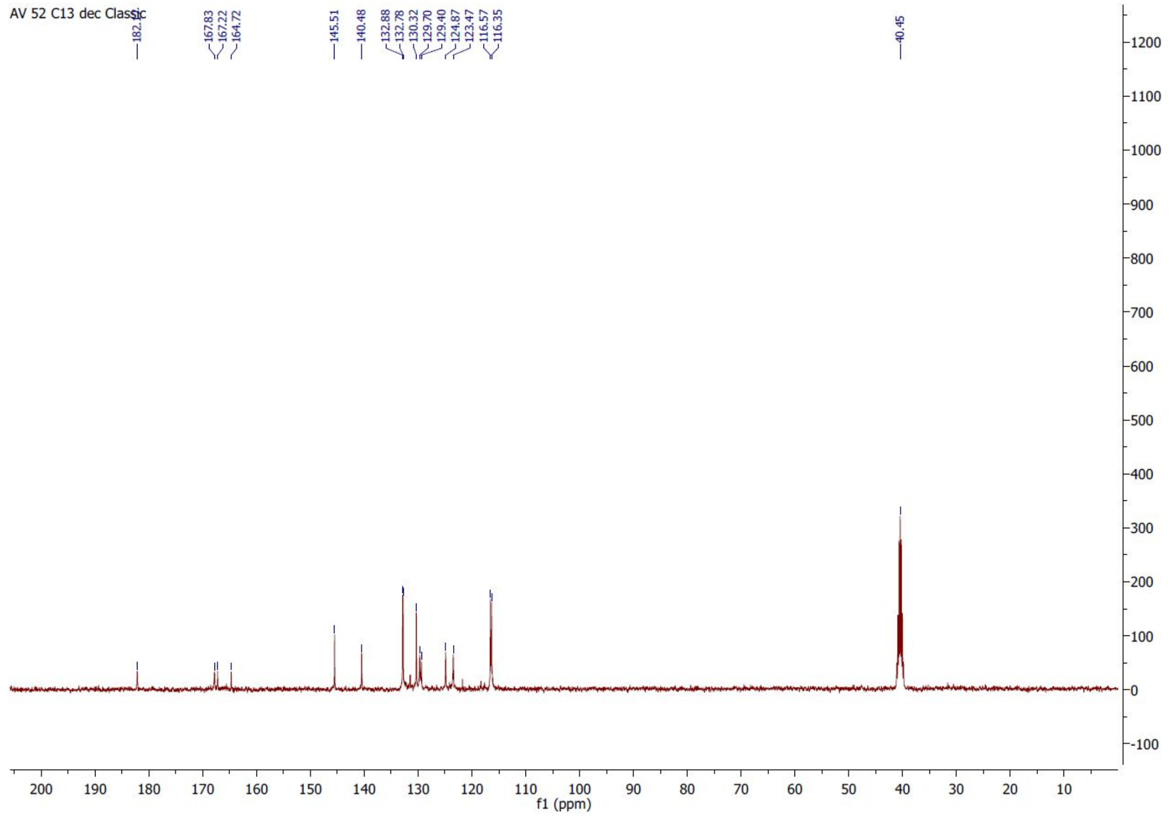

^{13}C NMR spectrum of compound **8d** (100 MHz, DMSO-d_6)

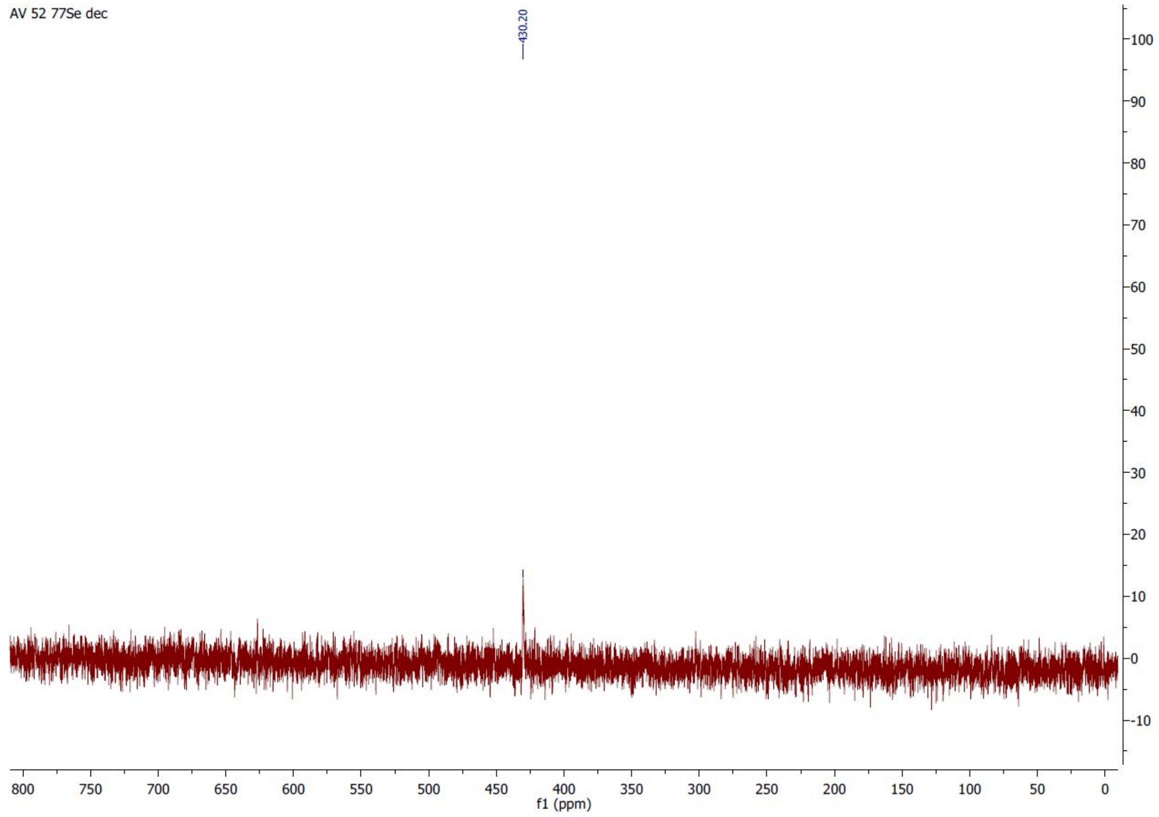

^{77}Se NMR spectrum of compound **8d** (76 MHz, DMSO-d_6)

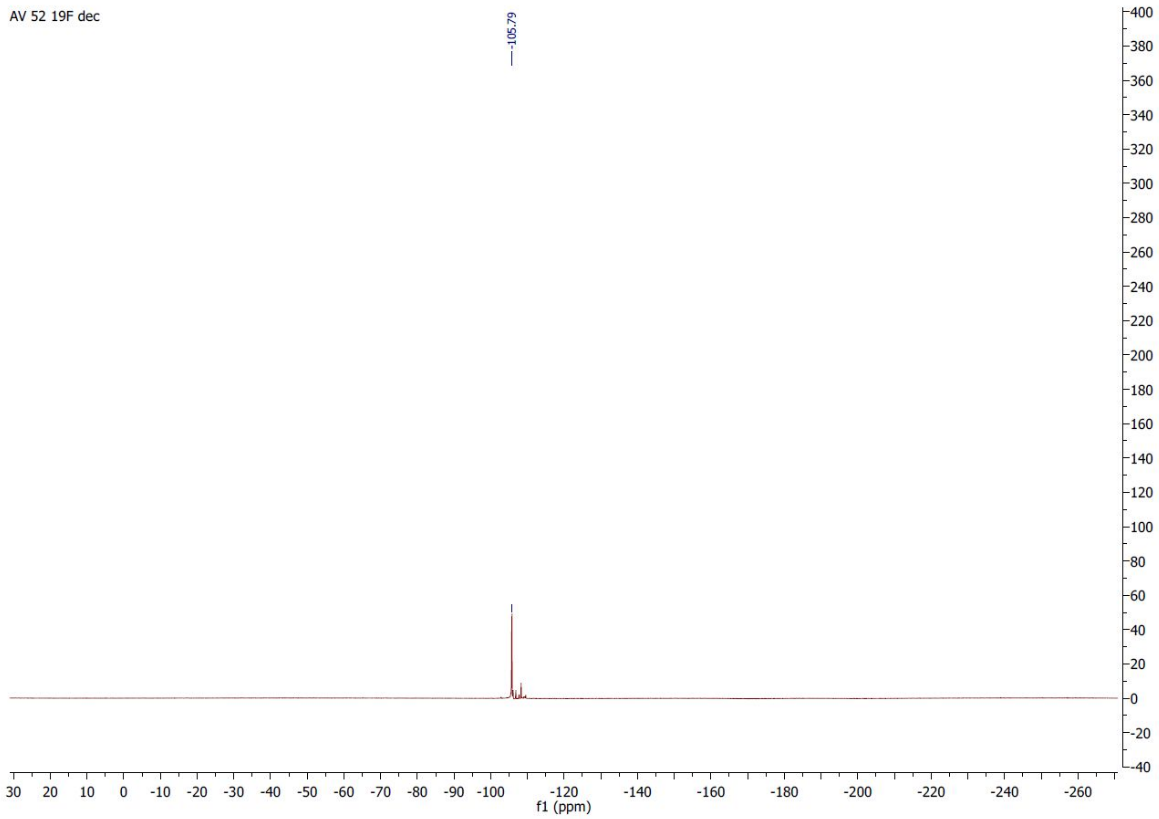

^{19}F NMR spectrum of compound **8d** (376 MHz, DMSO-d_6)

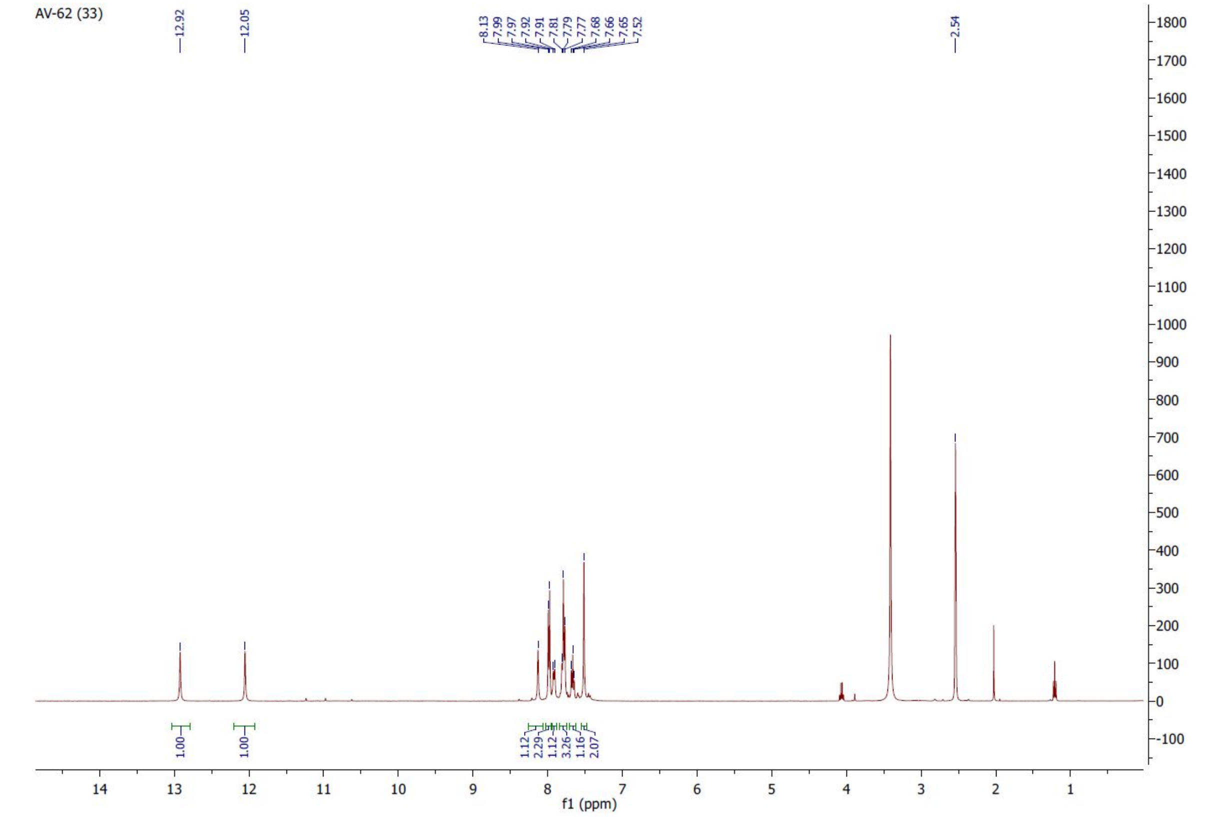

^{1}H NMR spectrum of compound **8e** (400 MHz, DMSO-d_6)

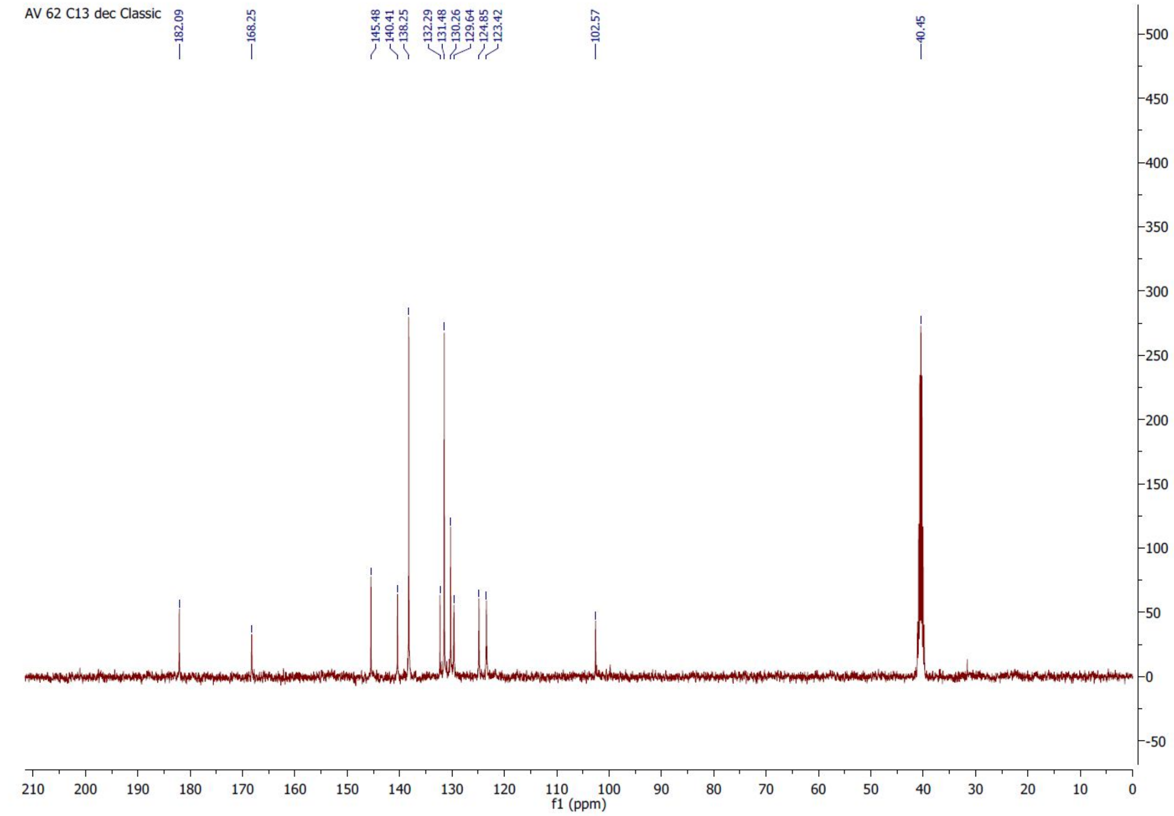

^{13}C NMR spectrum of compound **8e** (100 MHz, DMSO-d_6)

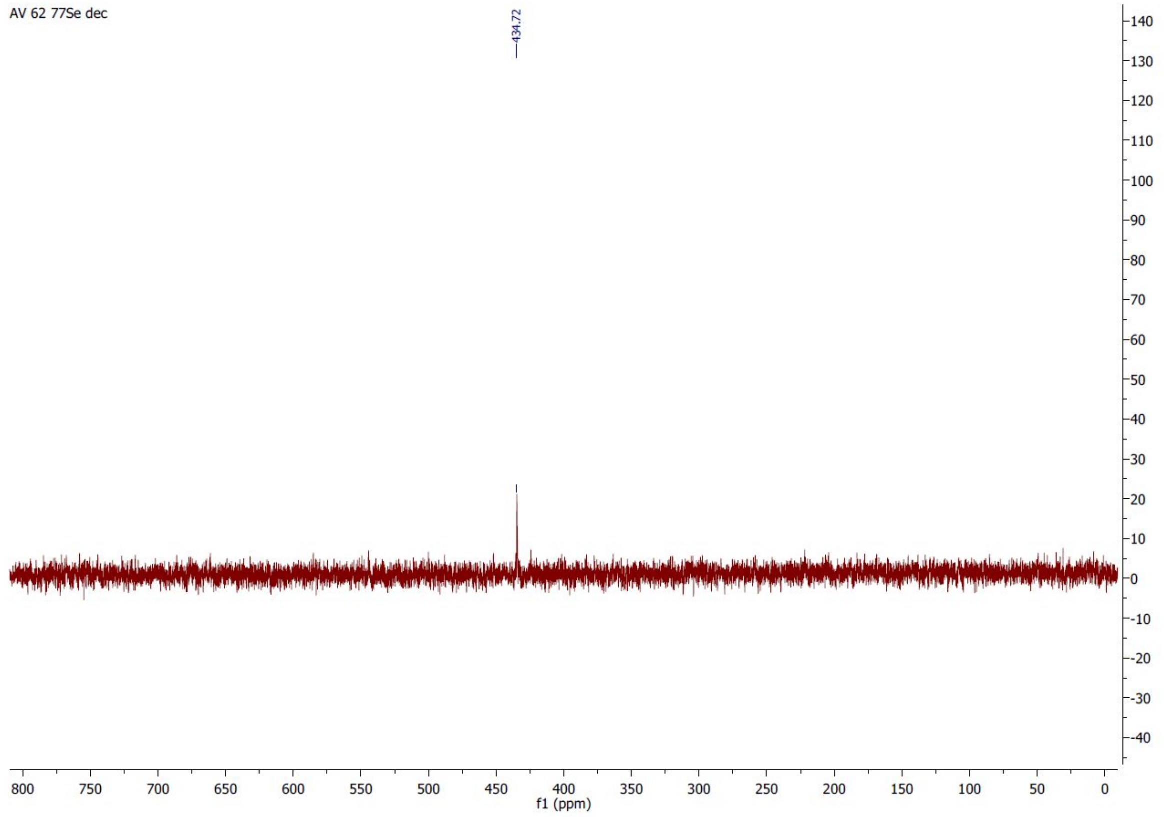

^{77}Se NMR spectrum of compound **8e** (76 MHz, DMSO-d_6)

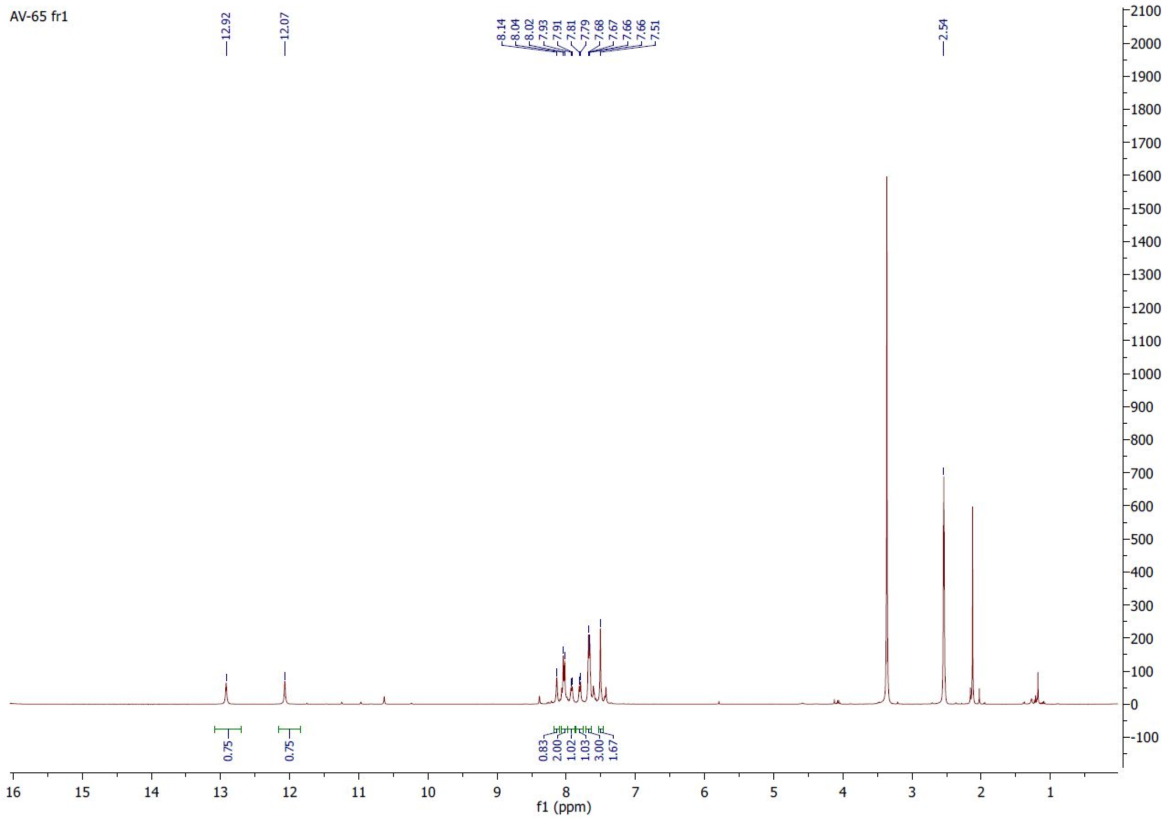

^{1}H NMR spectrum of compound **8f** (400 MHz, DMSO-d_6)

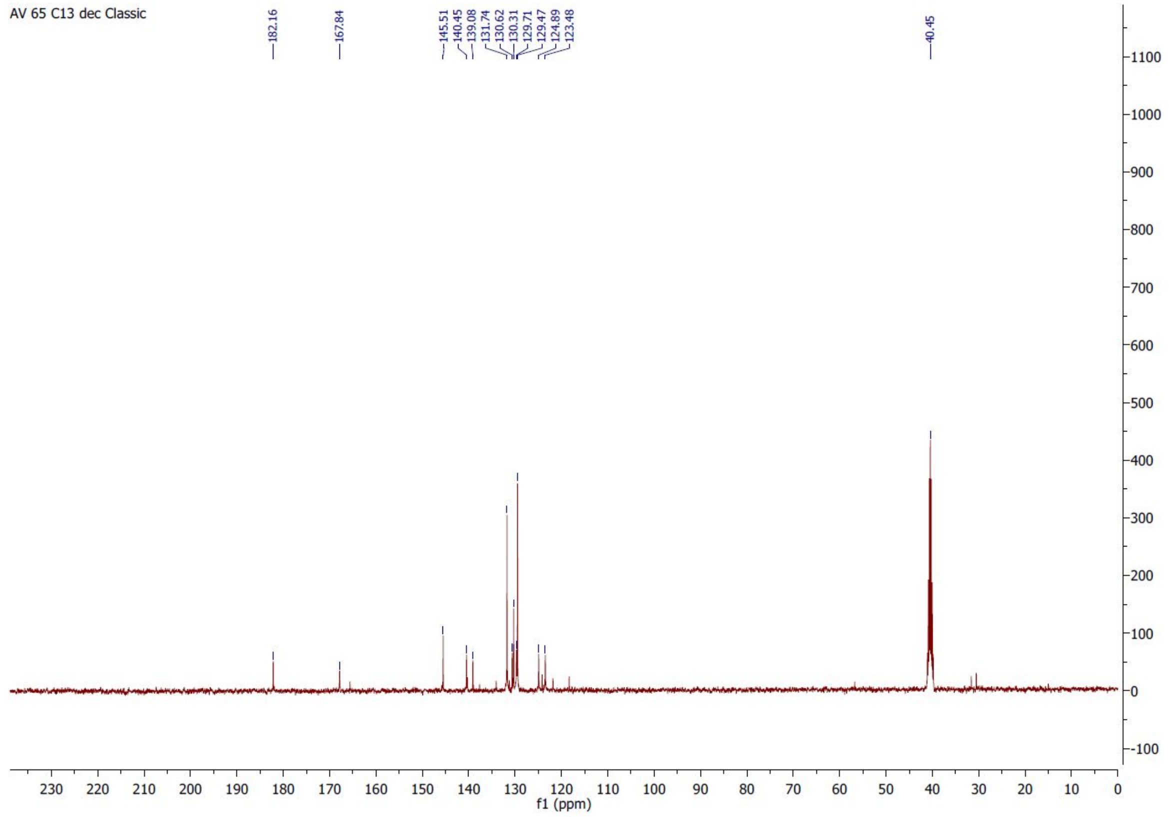

^{13}C NMR spectrum of compound **8f** (100 MHz, DMSO-d_6)

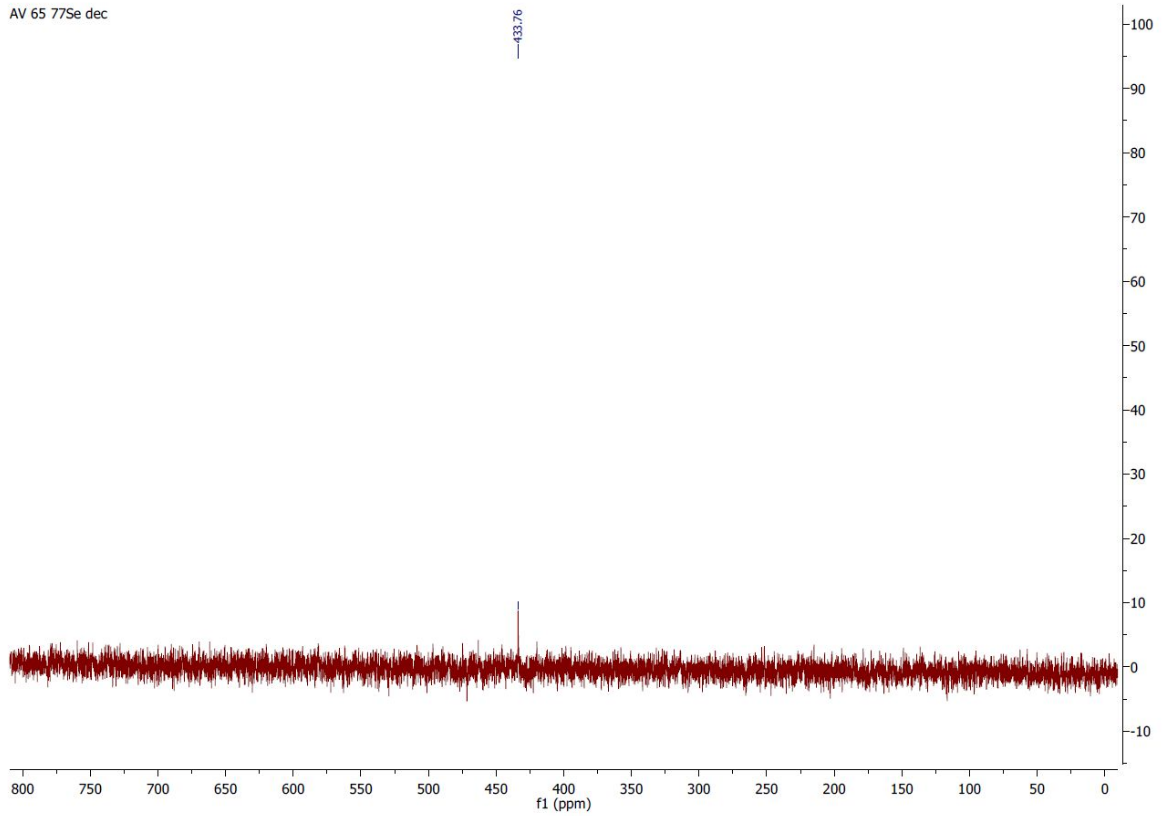

^{77}Se NMR spectrum of compound **8f** (76 MHz, DMSO-d_6)

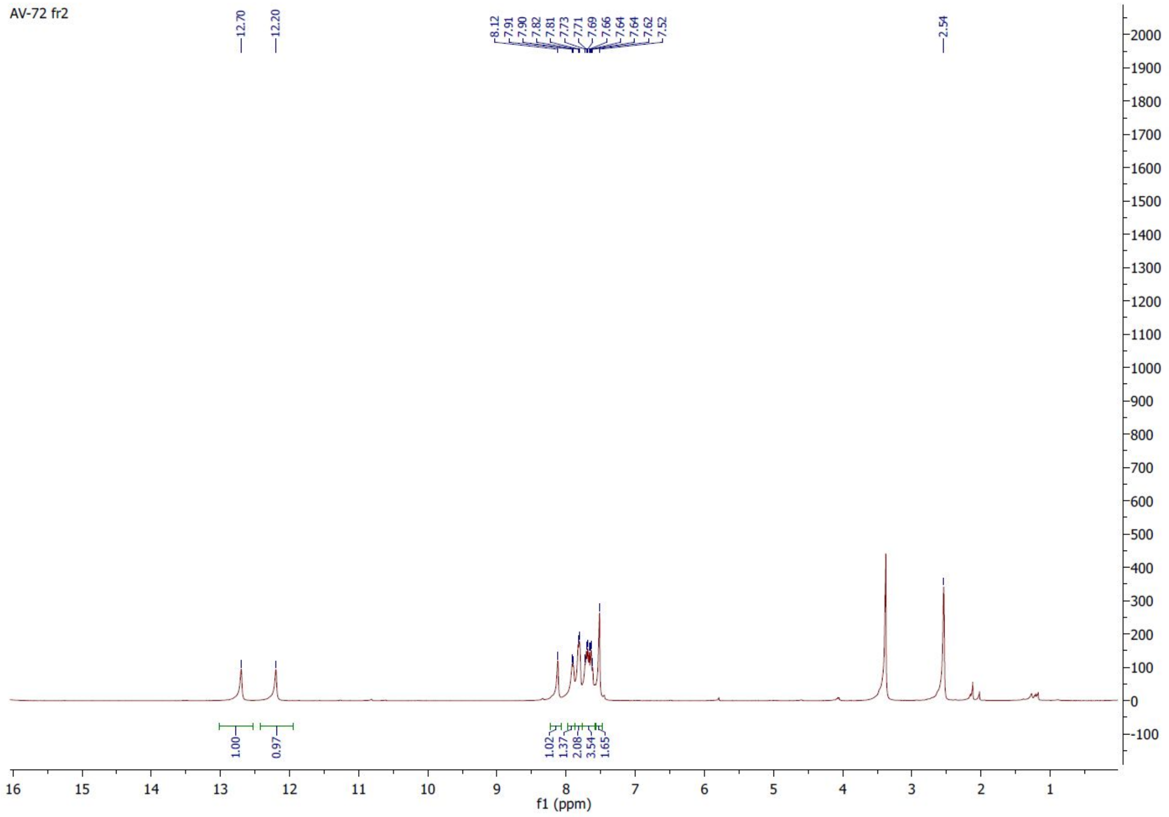

^{1}H NMR spectrum of compound **8g** (400 MHz, DMSO-d_6)

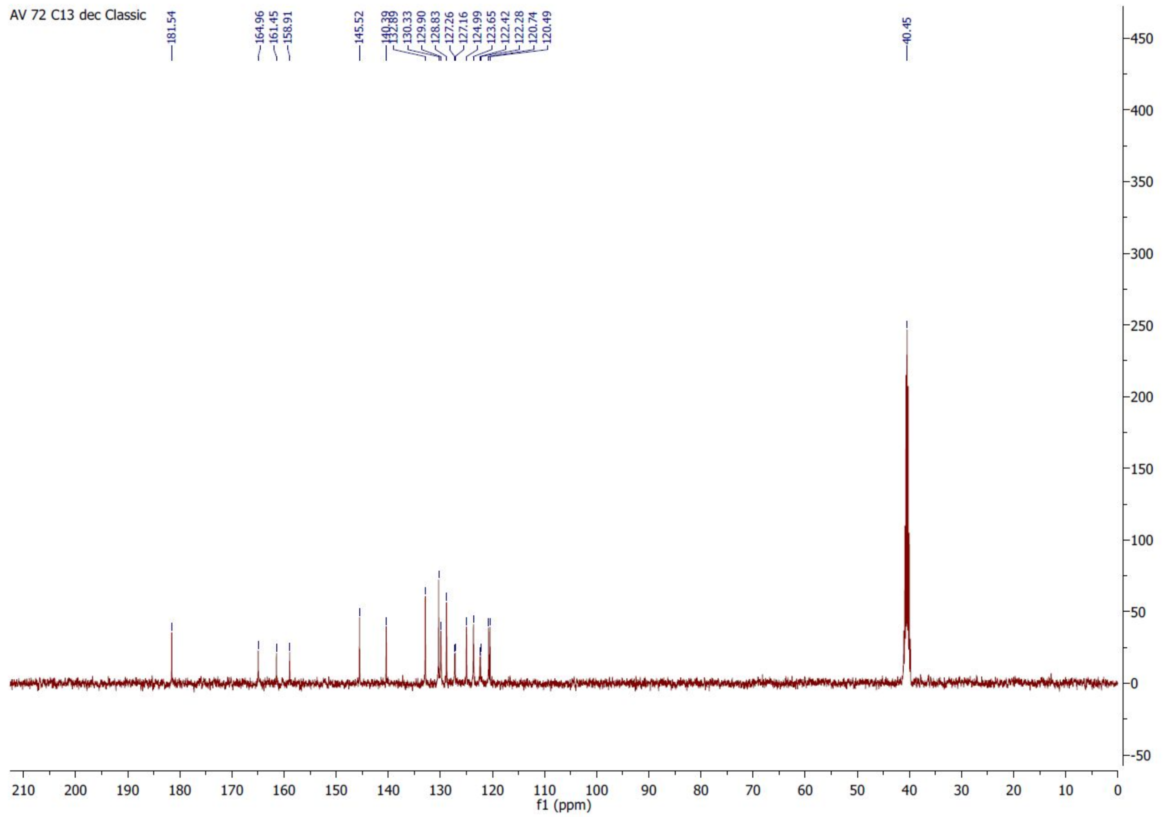

^{13}C NMR spectrum of compound **8g** (100 MHz, DMSO-d_6)

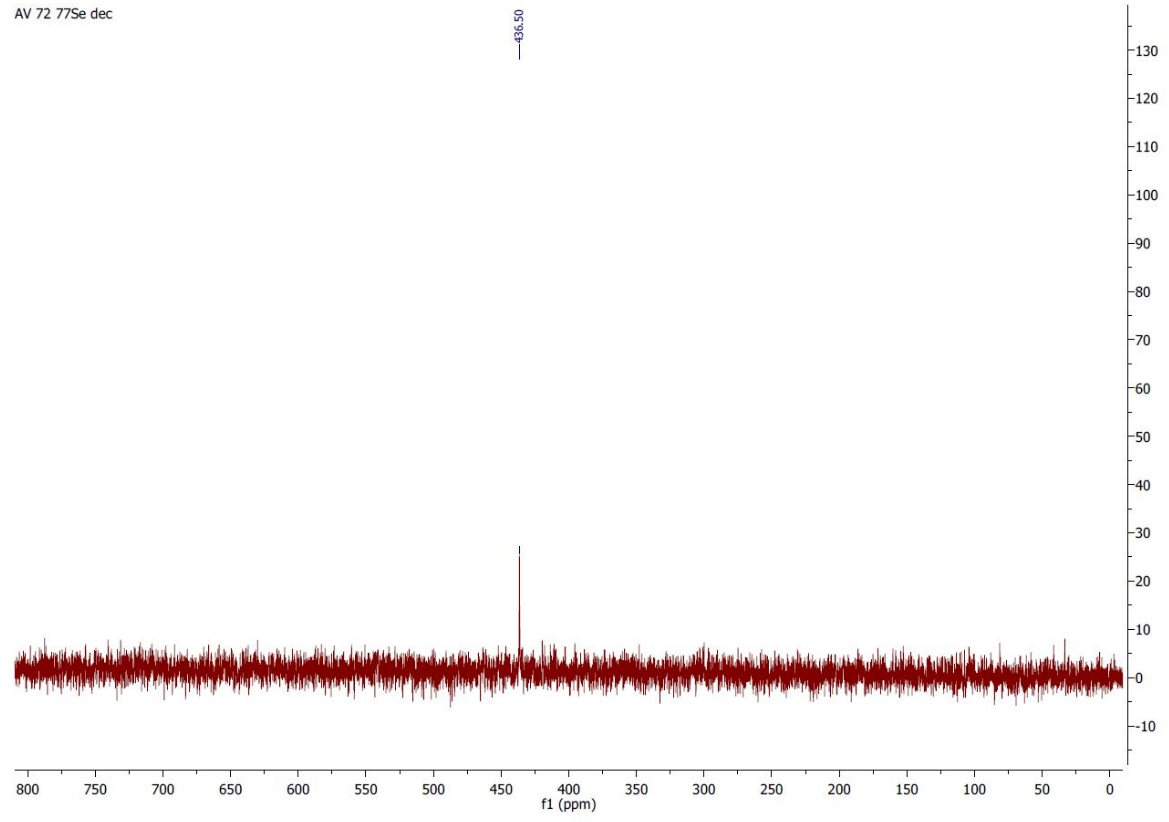

^{77}Se NMR spectrum of compound **8g** (76 MHz, DMSO-d_6)

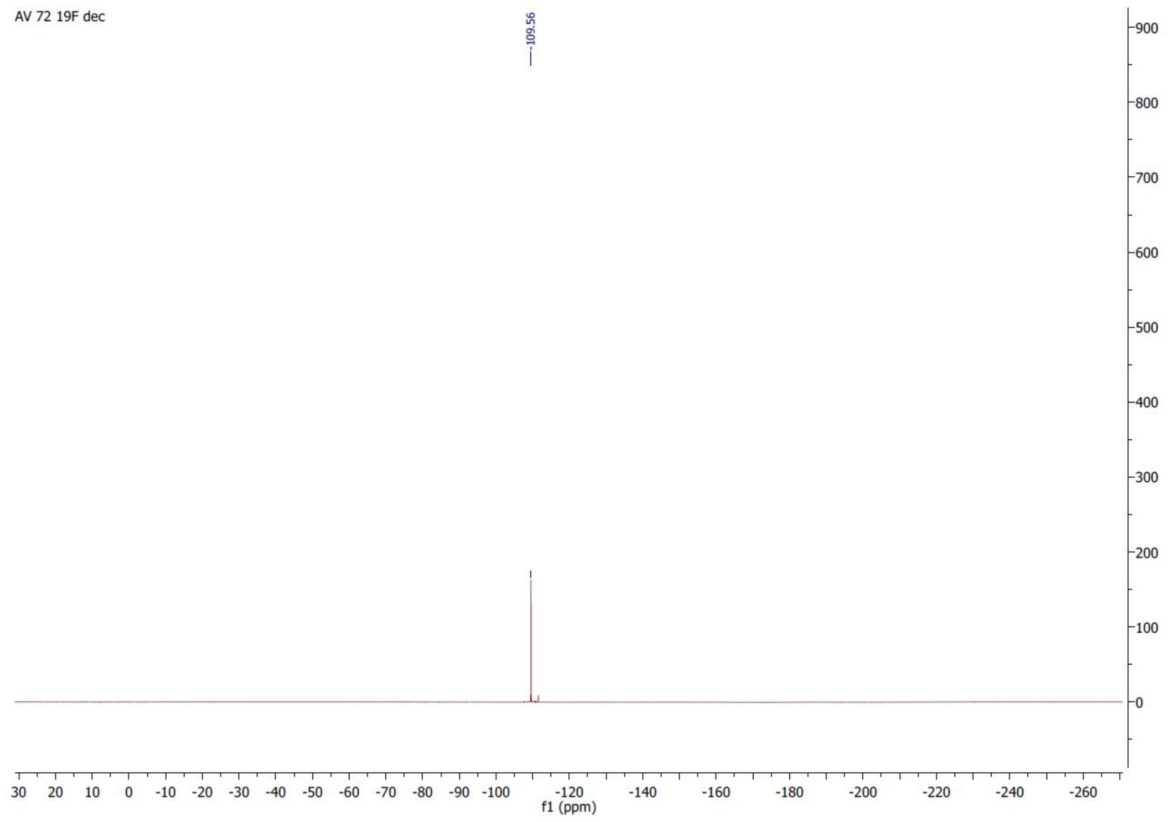

^{19}F NMR spectrum of compound **8g** (376 MHz, DMSO-d_6)

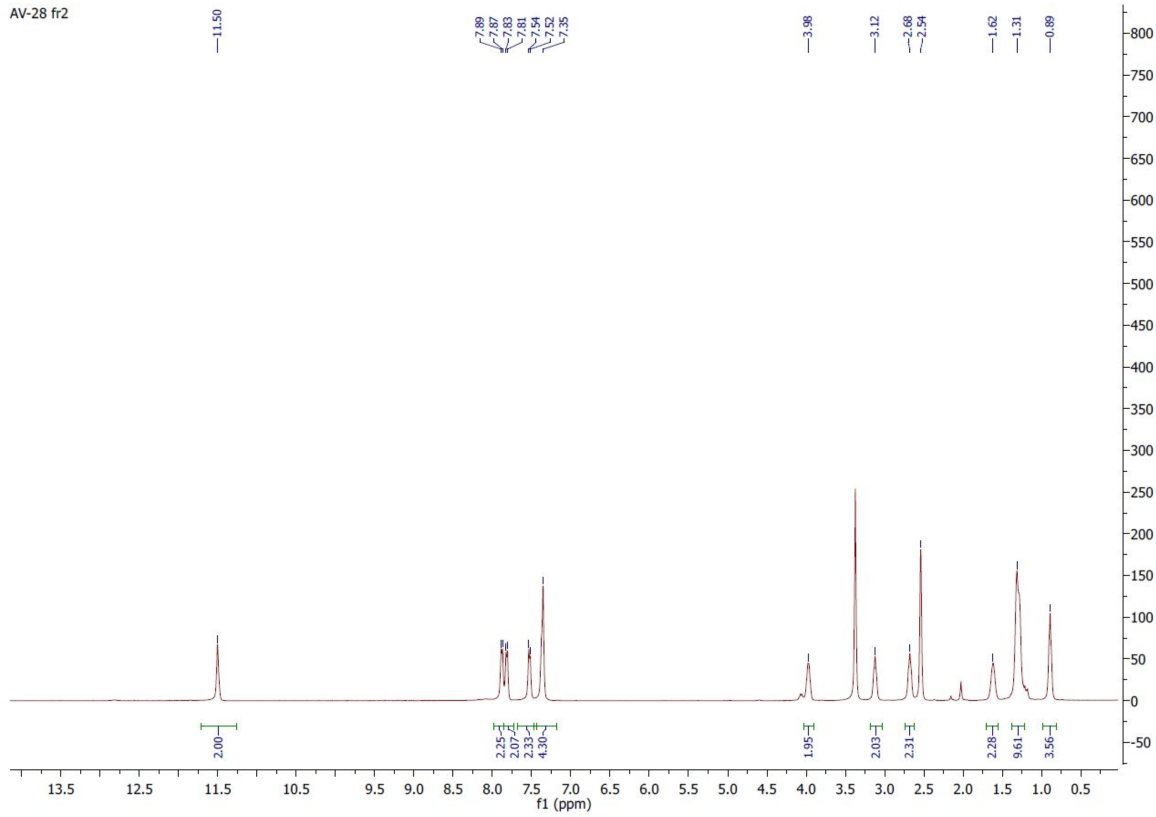

^{1}H NMR spectrum of compound **9c** (400 MHz, DMSO-d_6)

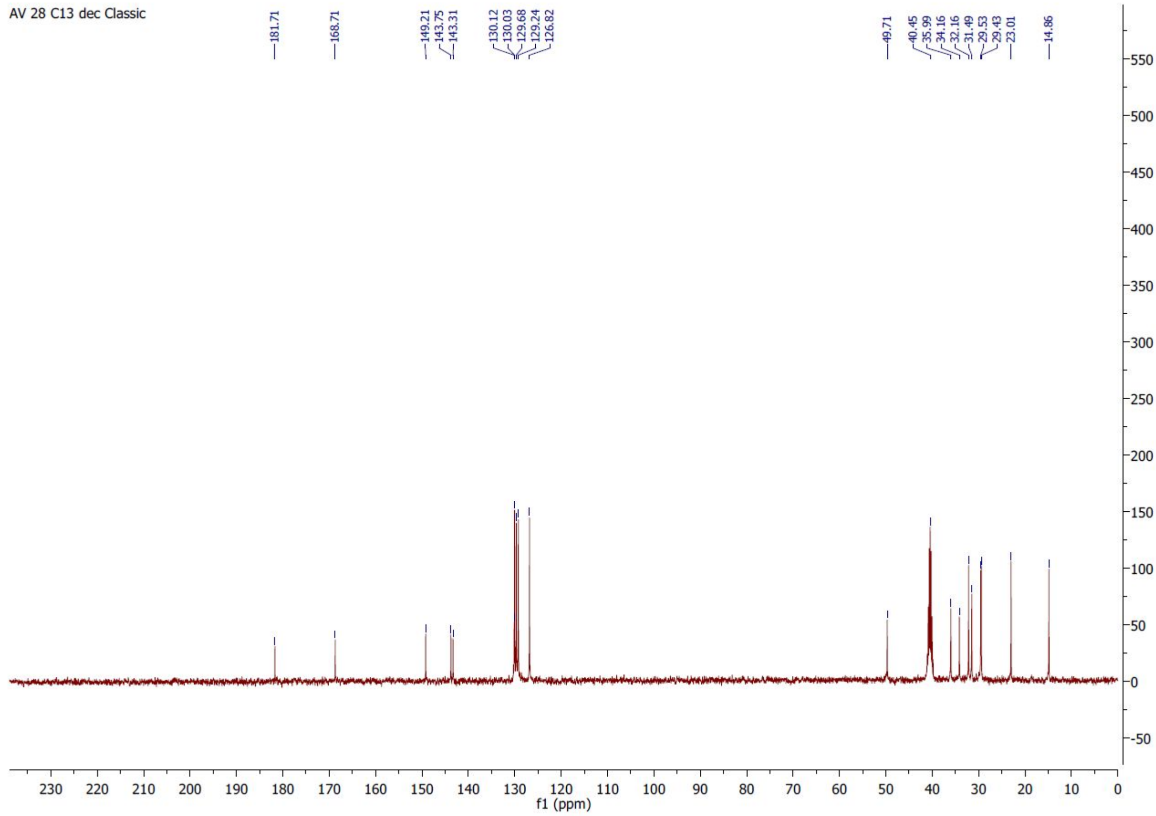

^{13}C NMR spectrum of compound **9c** (100 MHz, DMSO-d_6)

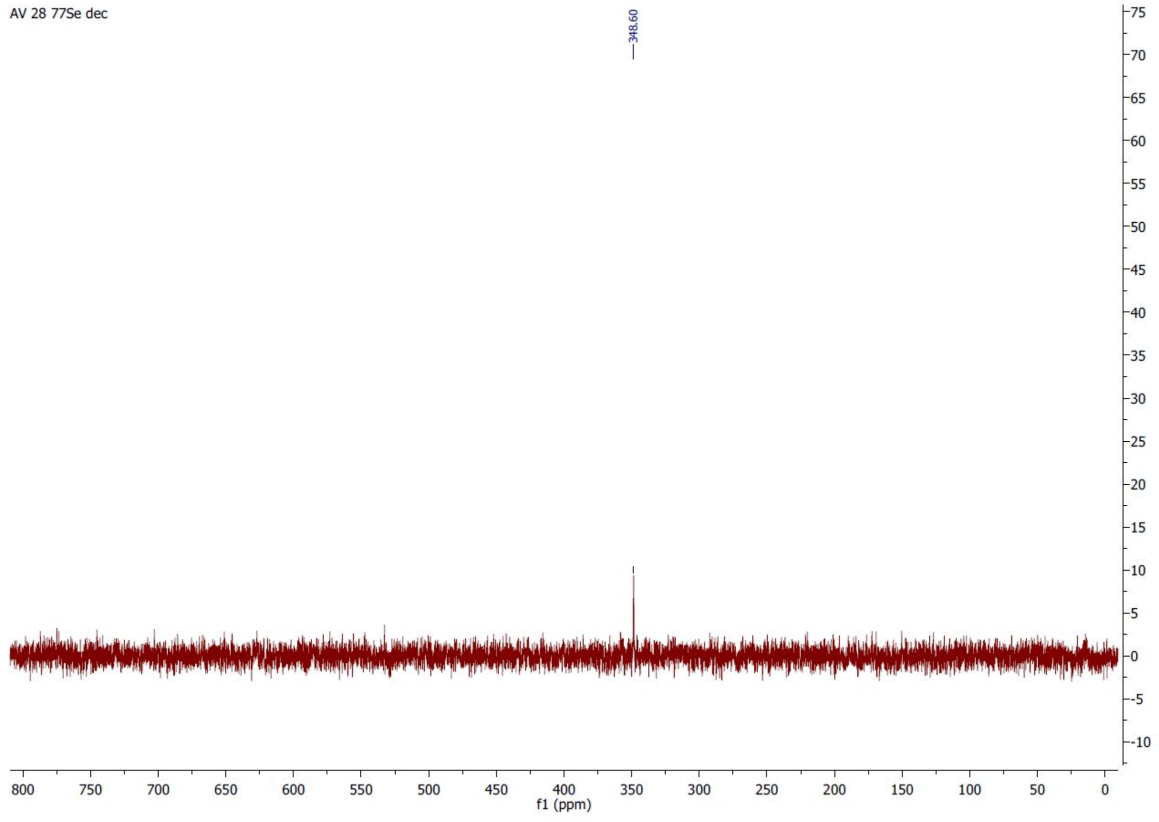

^{77}Se NMR spectrum of compound **9c** (76 MHz, DMSO-d_6)

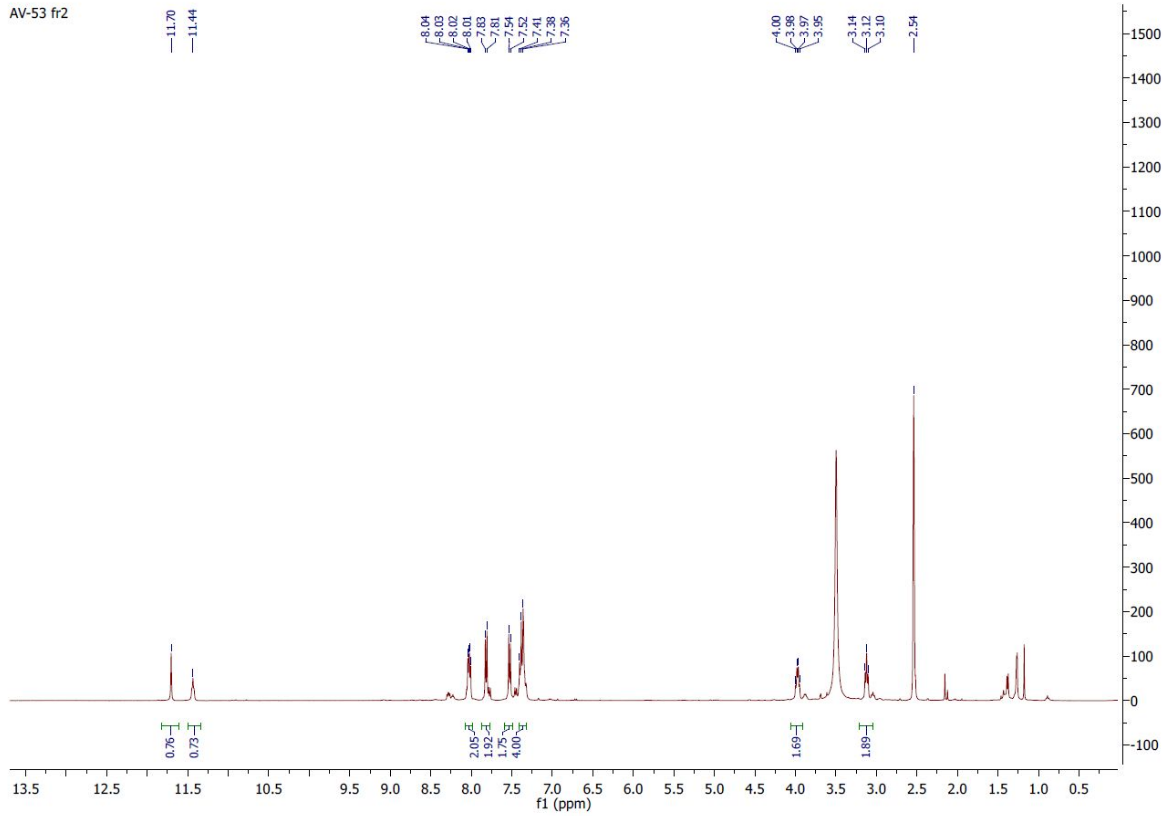

^{1}H NMR spectrum of compound **9e** (400 MHz, DMSO-d_6)

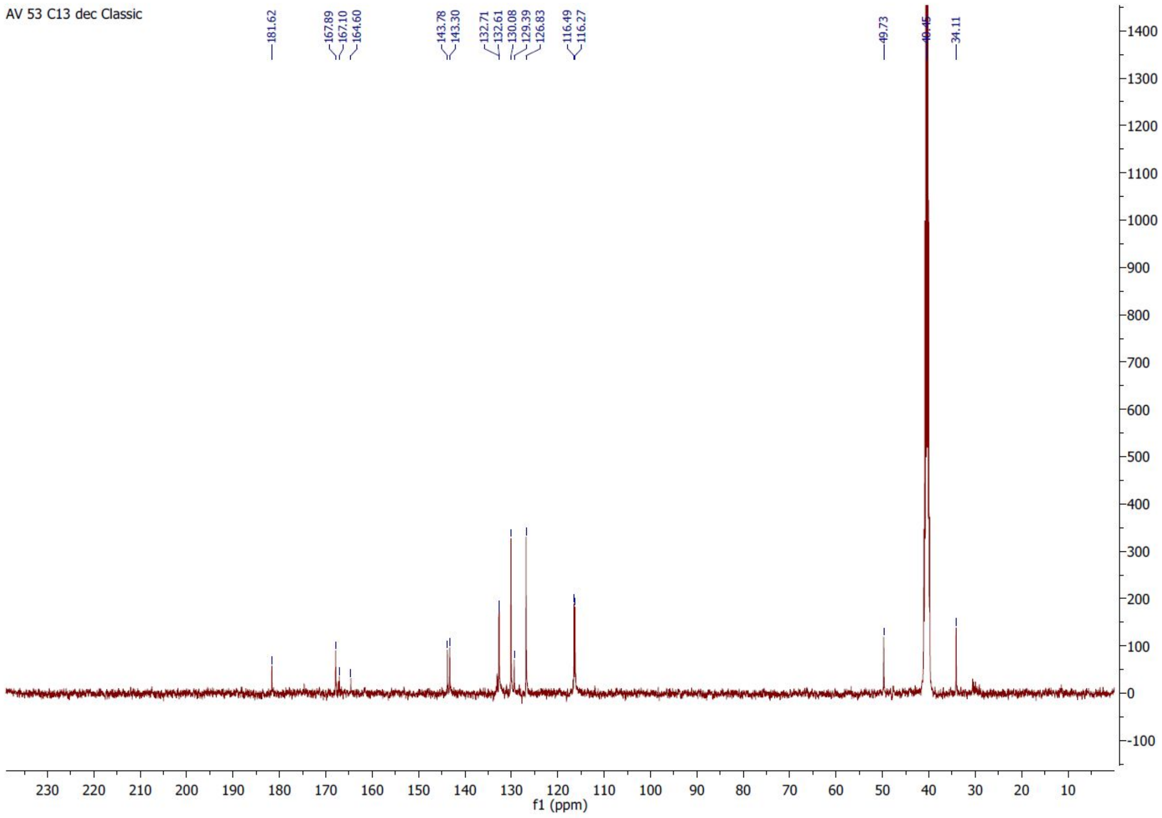

^{13}C NMR spectrum of compound **9e** (100 MHz, DMSO-d_6)

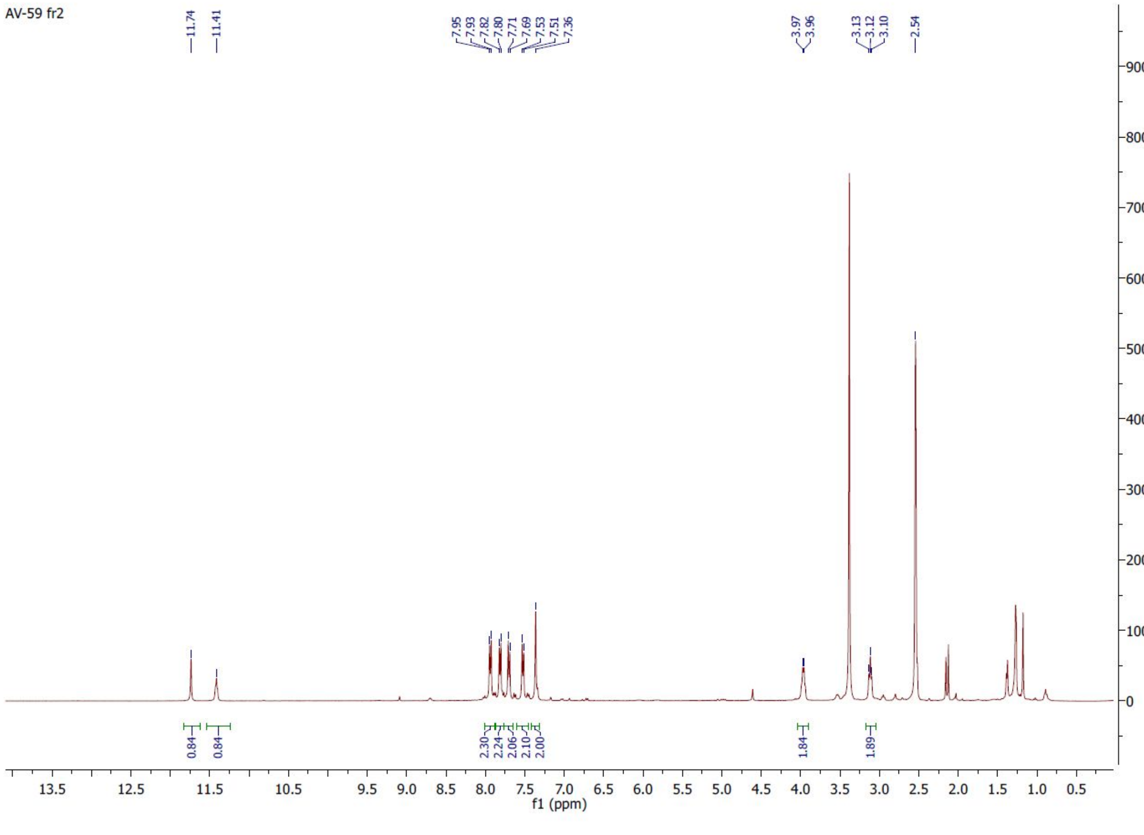

^{1}H NMR spectrum of compound **9f** (400 MHz, DMSO-d_6)

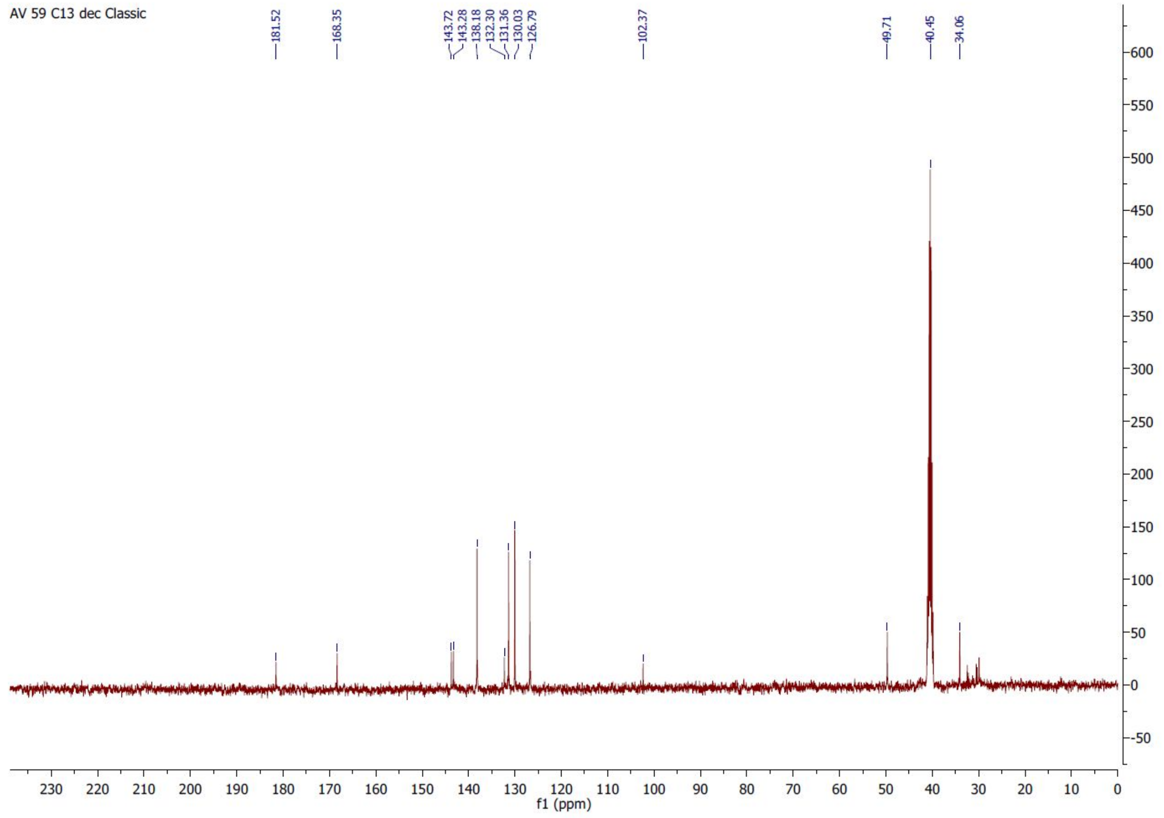

^{13}C NMR spectrum of compound **9f** (100 MHz, DMSO-d_6)

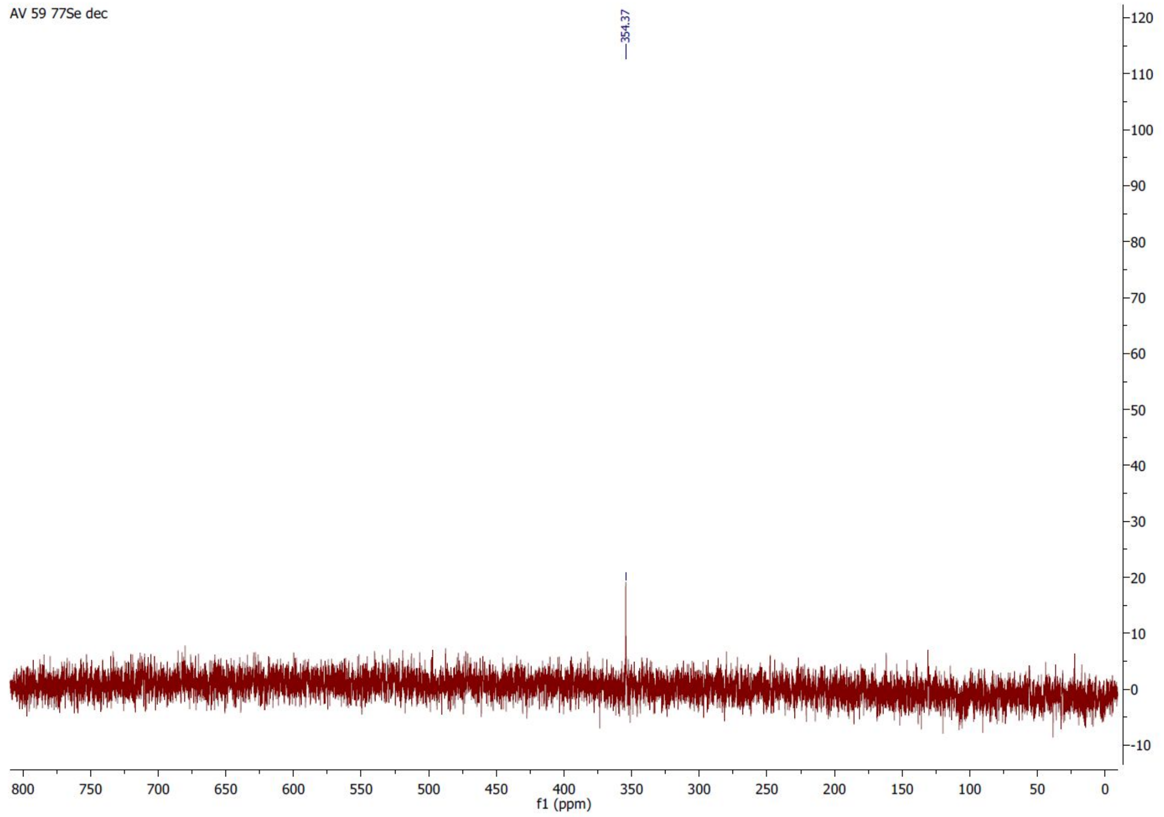

^{77}Se NMR spectrum of compound **9f** (76 MHz, DMSO-d_6)

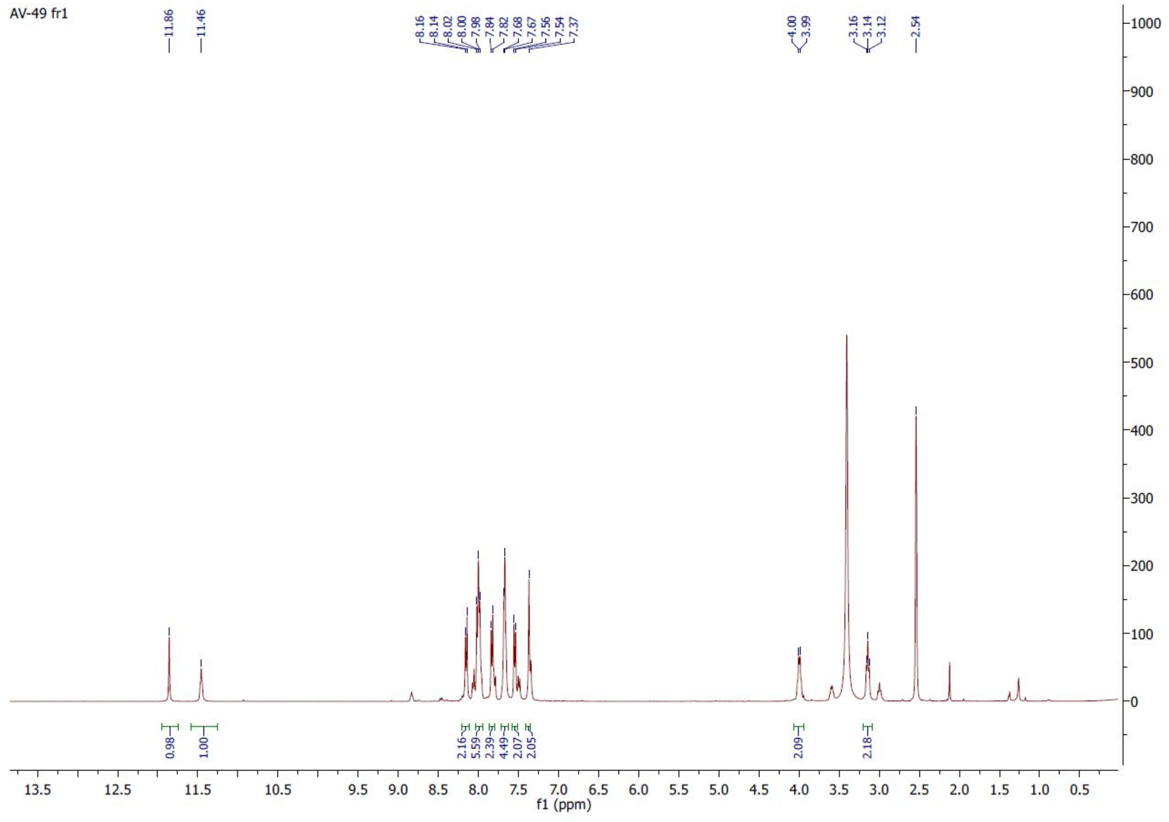

^{1}H NMR spectrum of compound **9g** (400 MHz, DMSO-d_6)

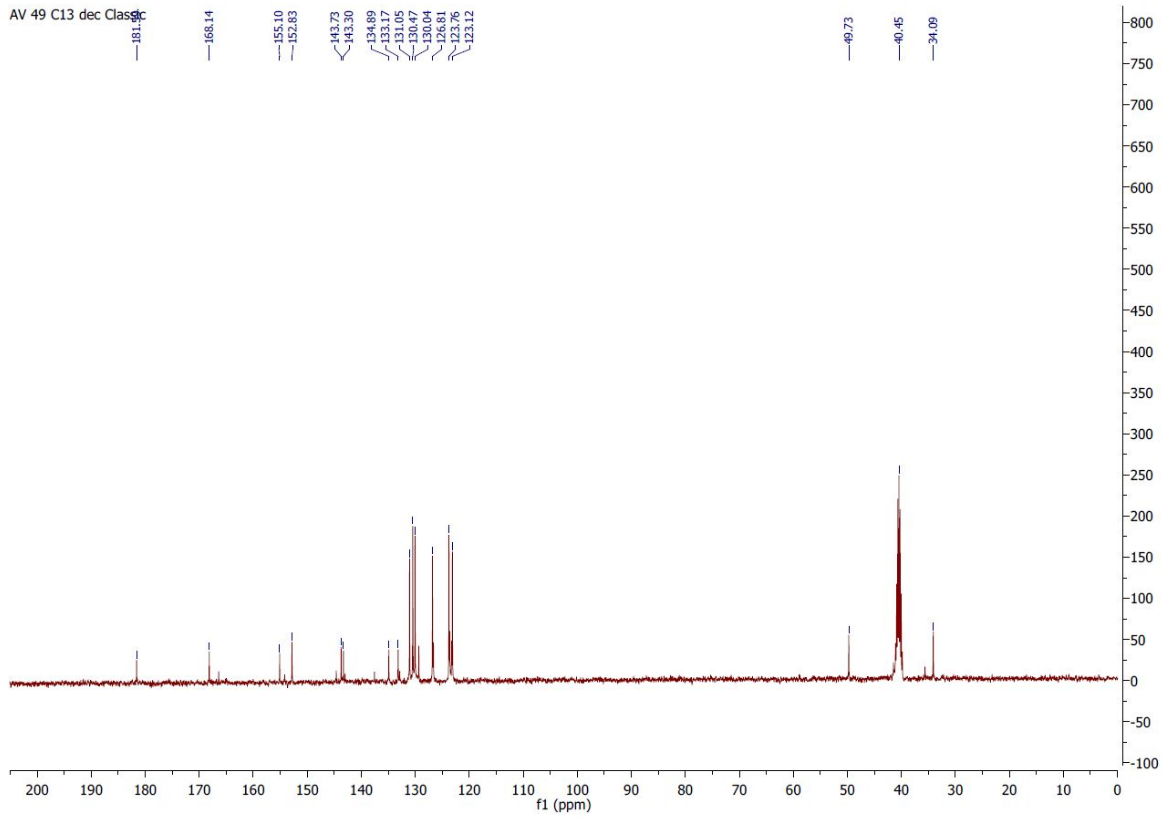

^{13}C NMR spectrum of compound **9g** (100 MHz, DMSO-d_6)

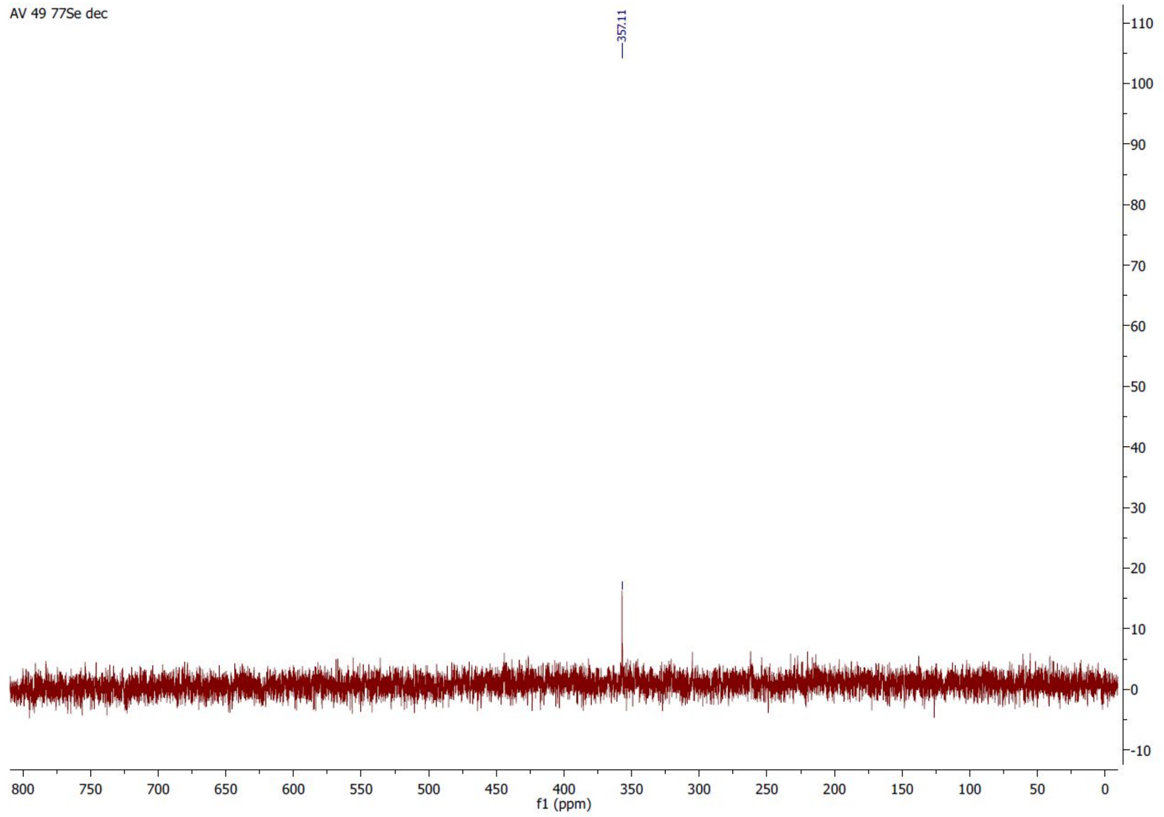

^{77}Se NMR spectrum of compound **9g** (76 MHz, DMSO-d_6)

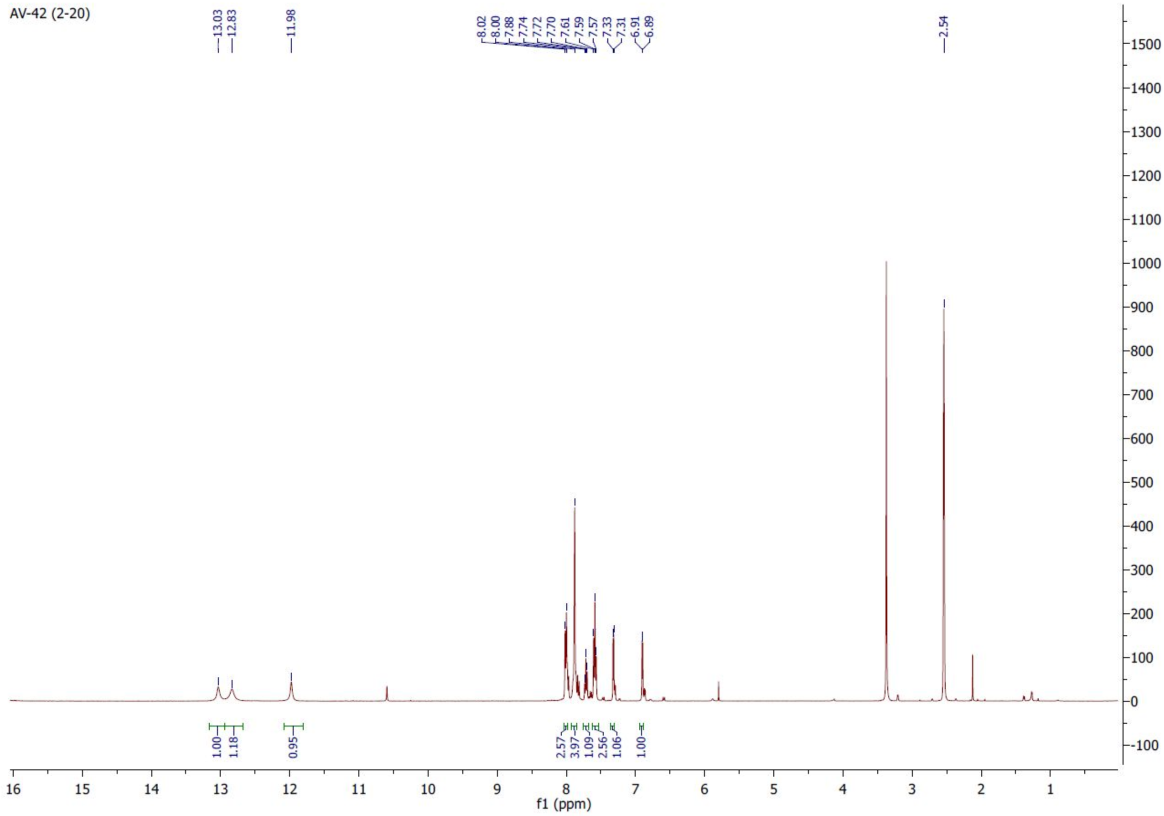

^{1}H NMR spectrum of compound **10a** (400 MHz, DMSO-d_6)

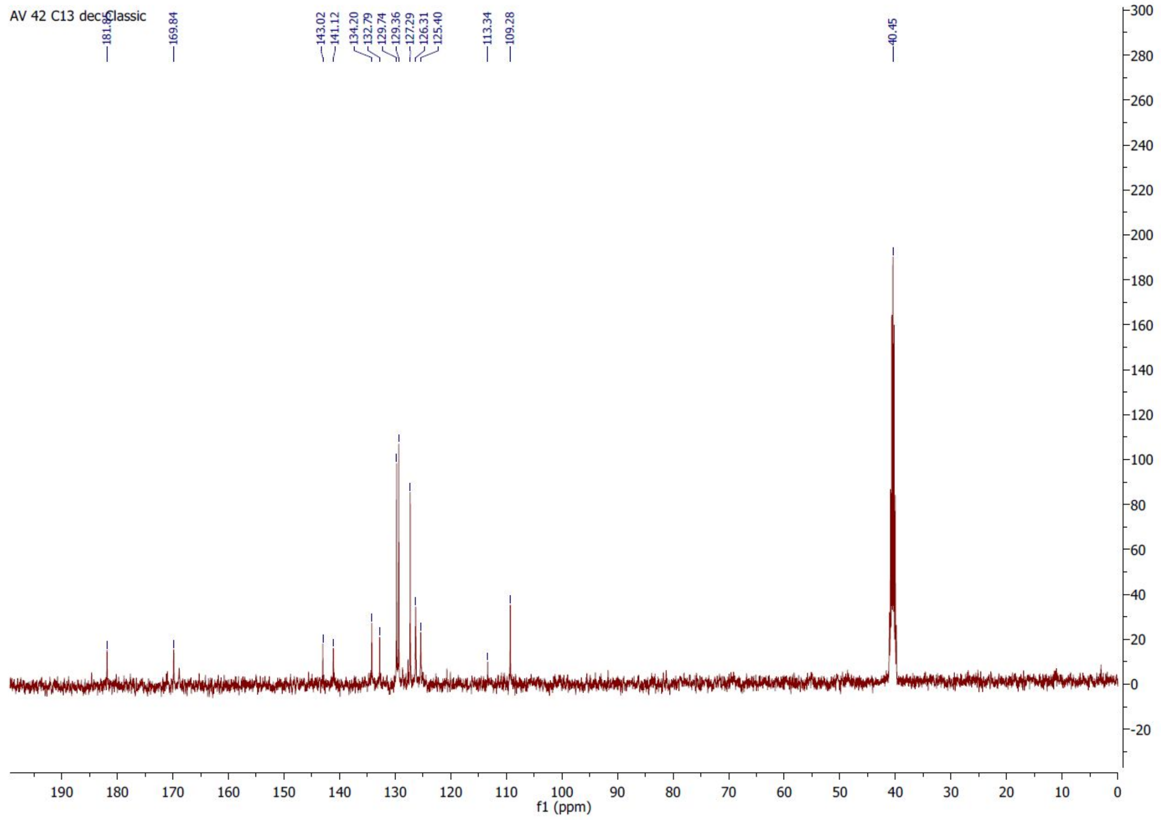

^{13}C NMR spectrum of compound **10a** (100 MHz, DMSO-d_6)

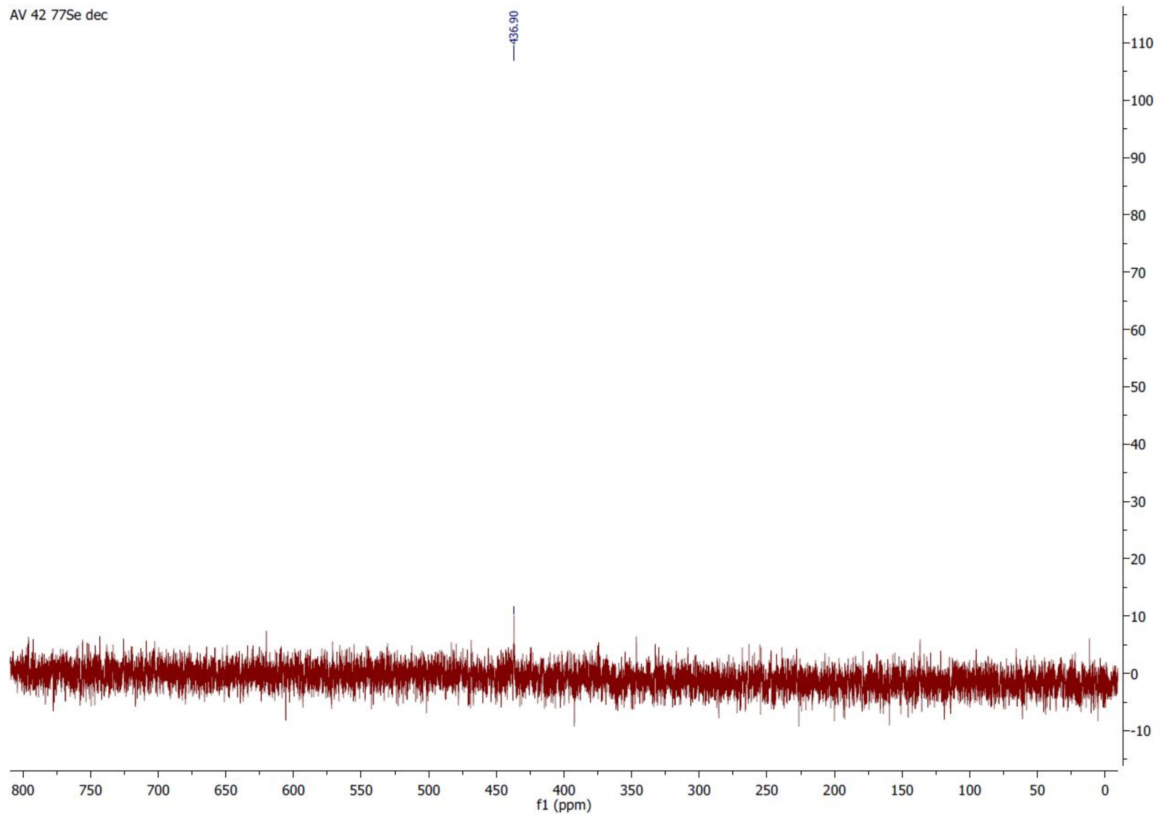

^{77}Se NMR spectrum of compound **10a** (76 MHz, DMSO-d_6)

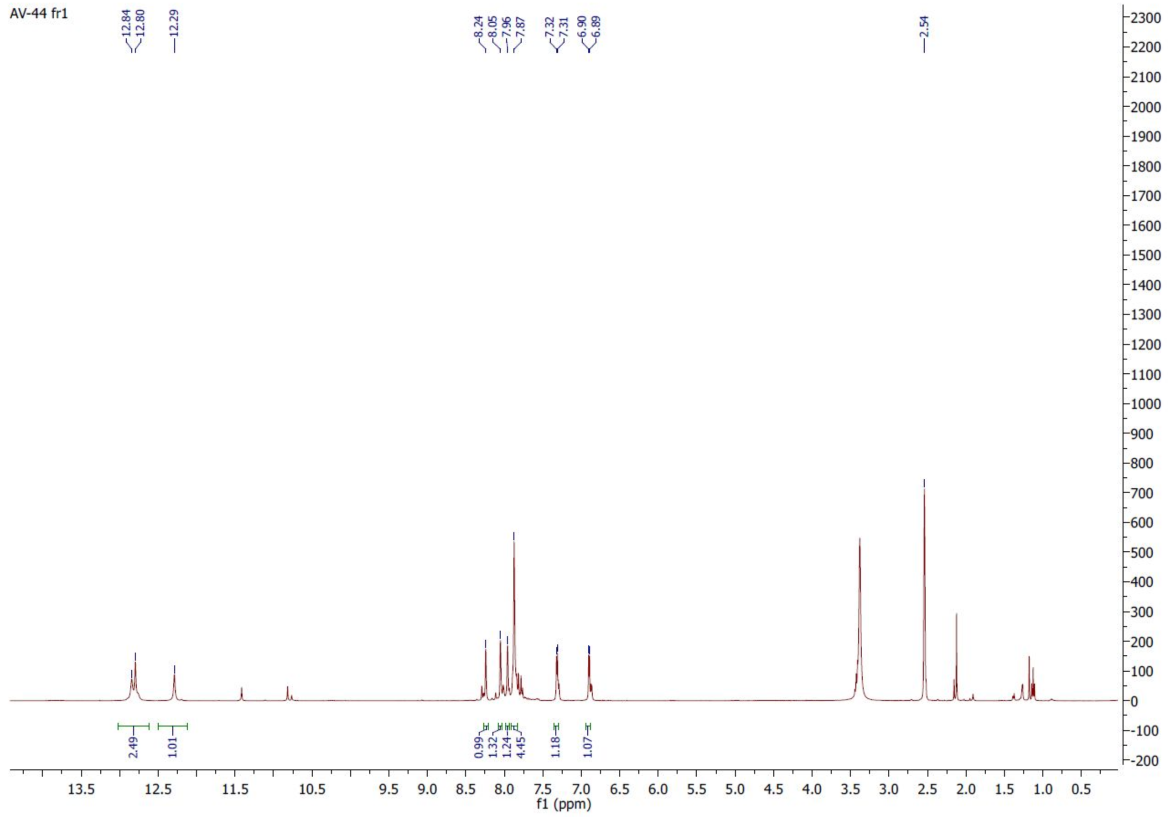

^{1}H NMR spectrum of compound **10b** (400 MHz, DMSO-d_6)

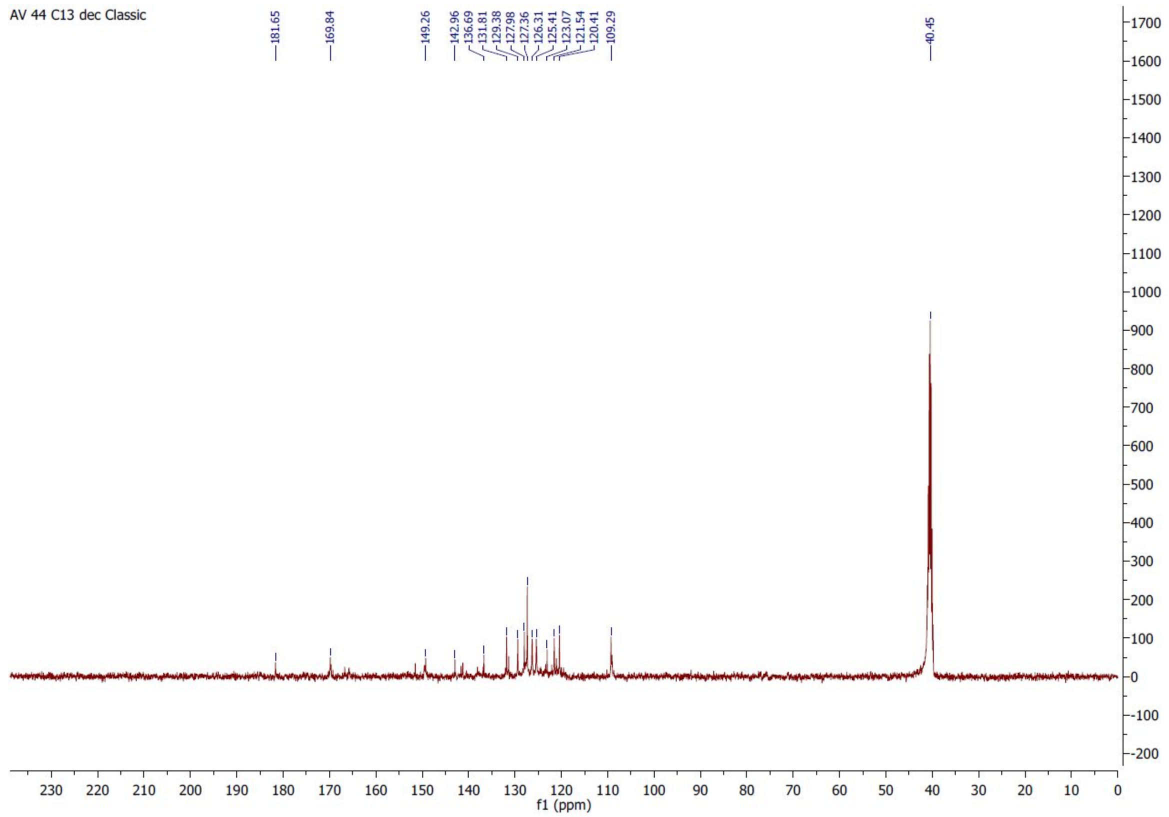

^{13}C NMR spectrum of compound **10b** (100 MHz, DMSO-d_6)

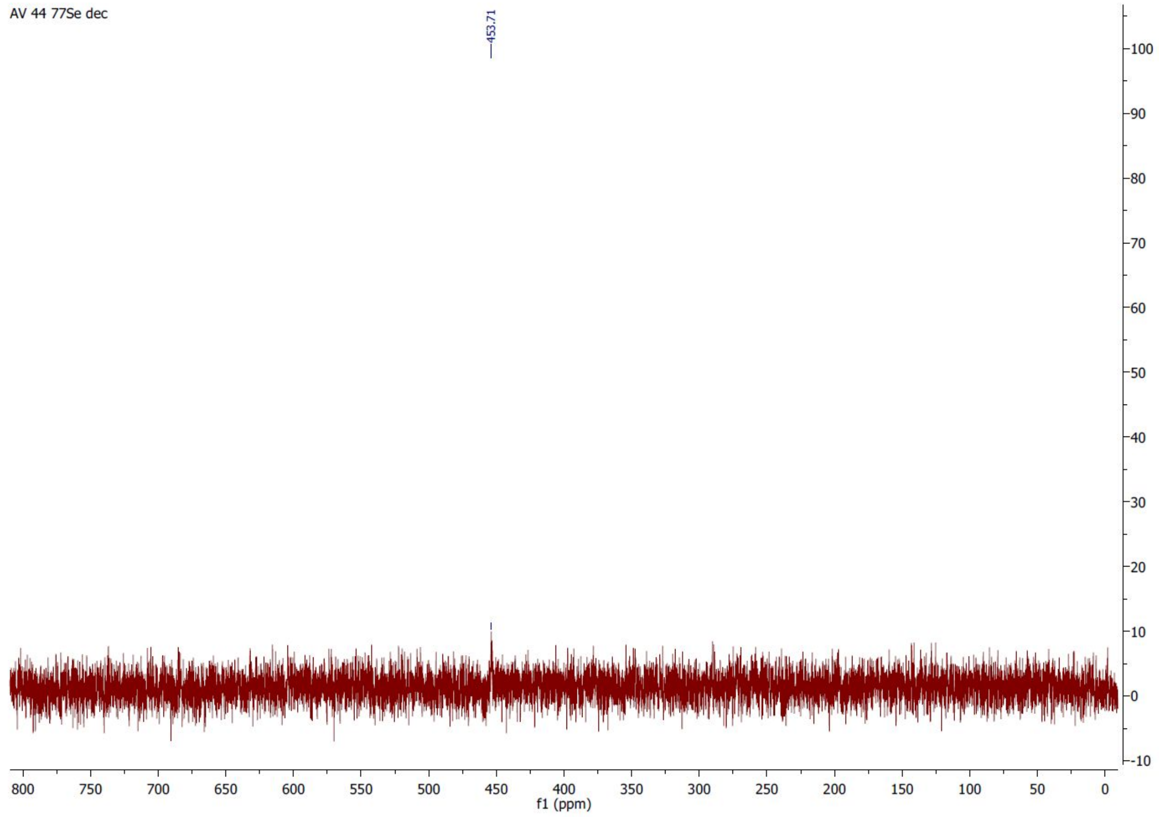

^{77}Se NMR spectrum of compound **10b** (76 MHz, DMSO-d_6)

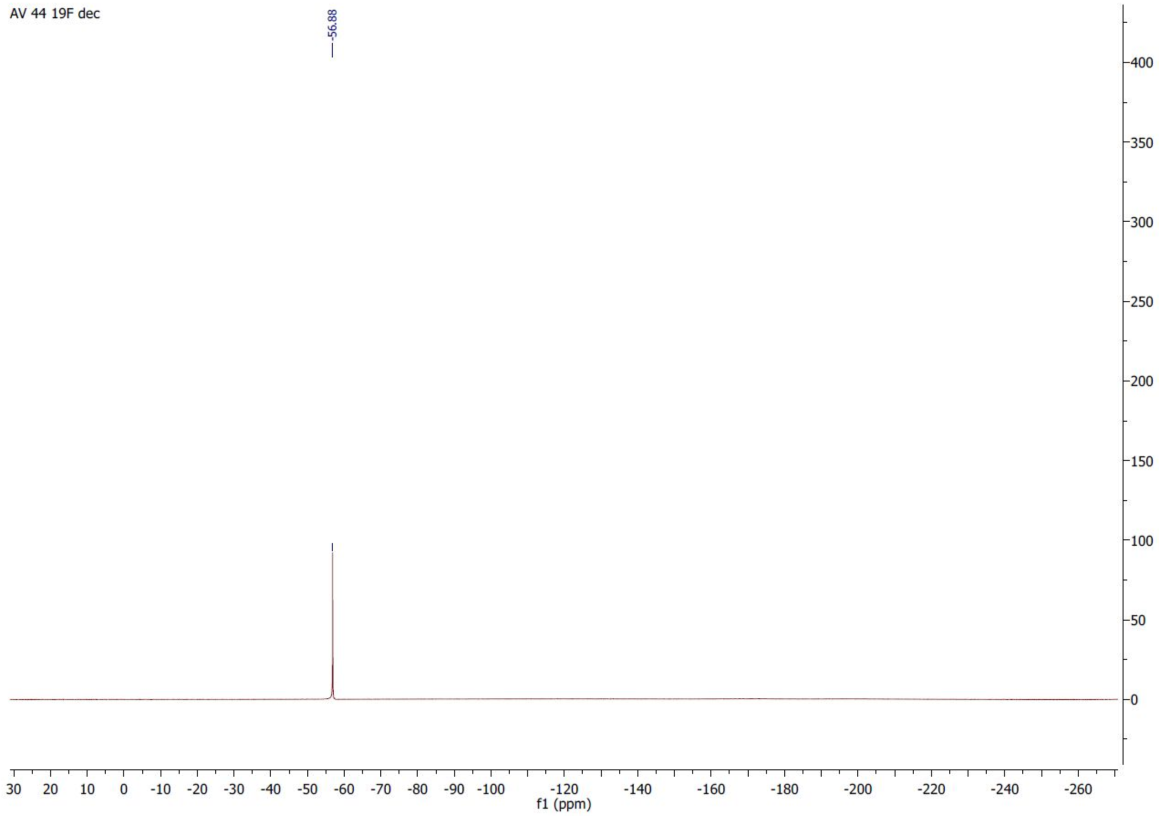

^{19}F NMR spectrum of compound **10b** (376 MHz, DMSO-d_6)

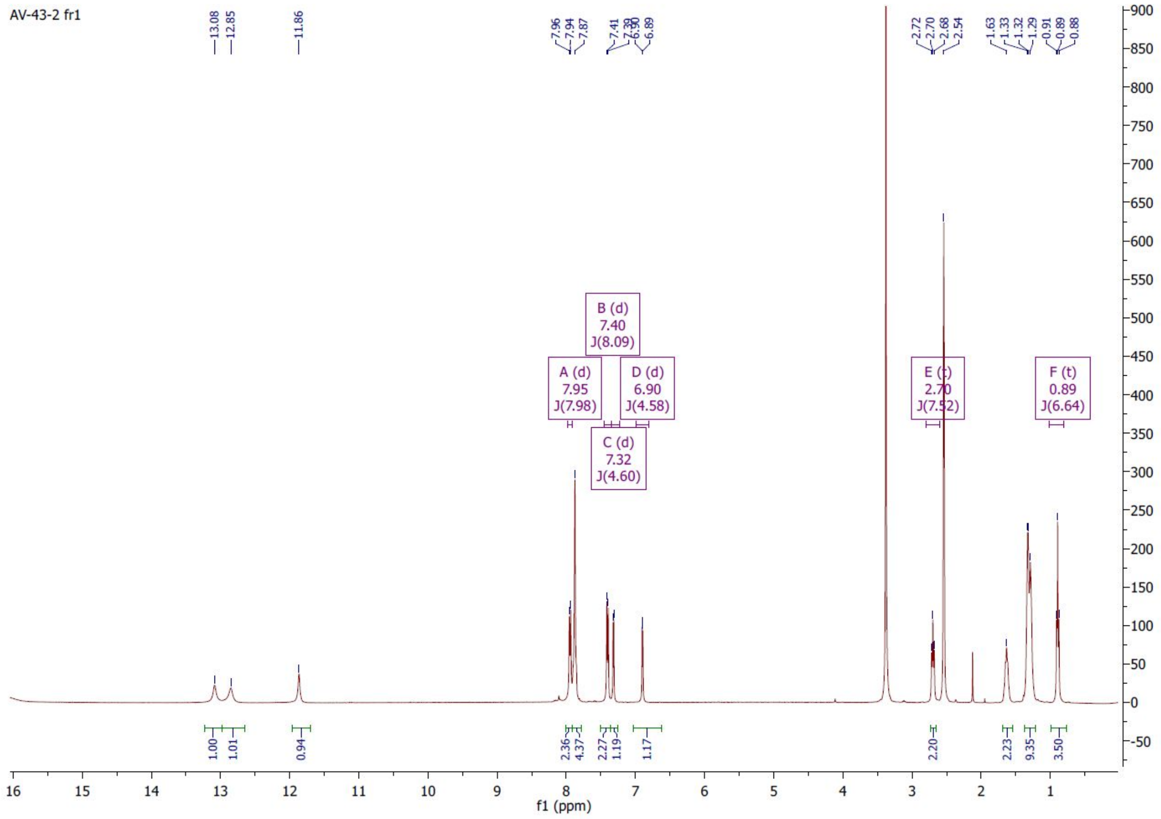

^{1}H NMR spectrum of compound **10c** (400 MHz, DMSO-d_6)

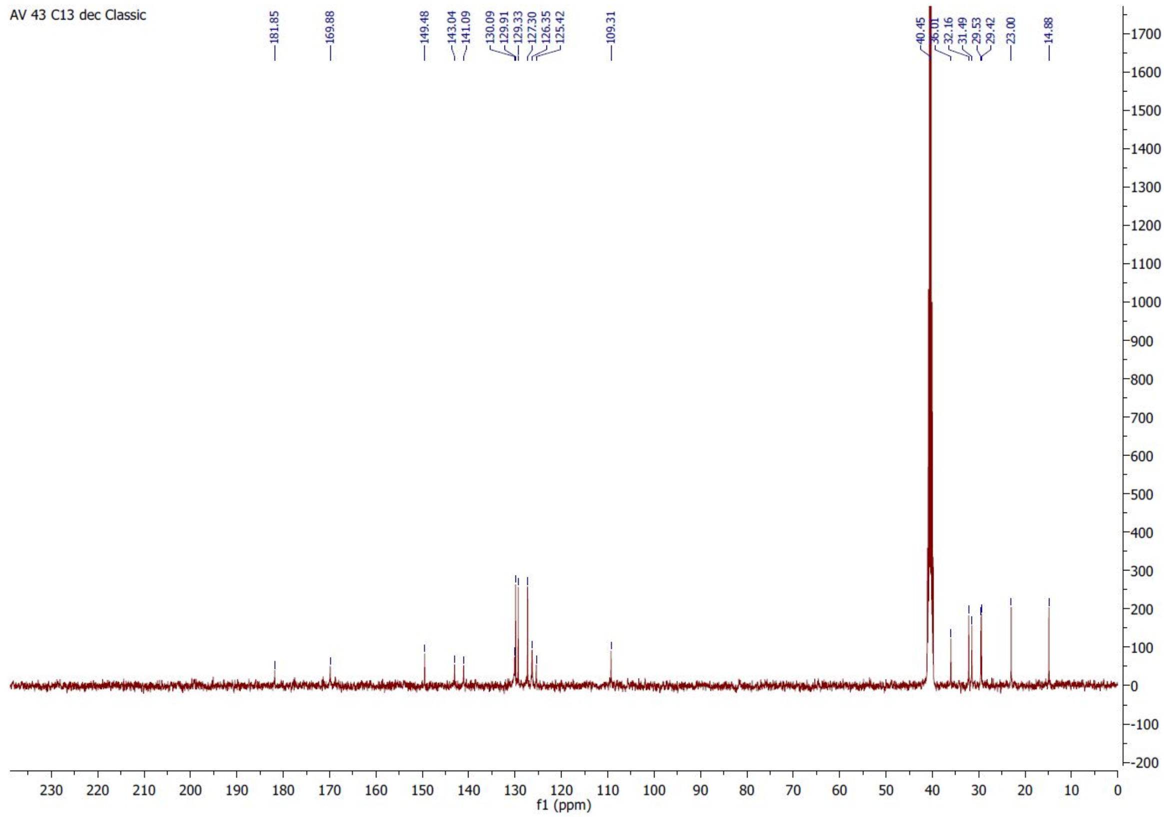

^{13}C NMR spectrum of compound **10c** (100 MHz, DMSO-d_6)

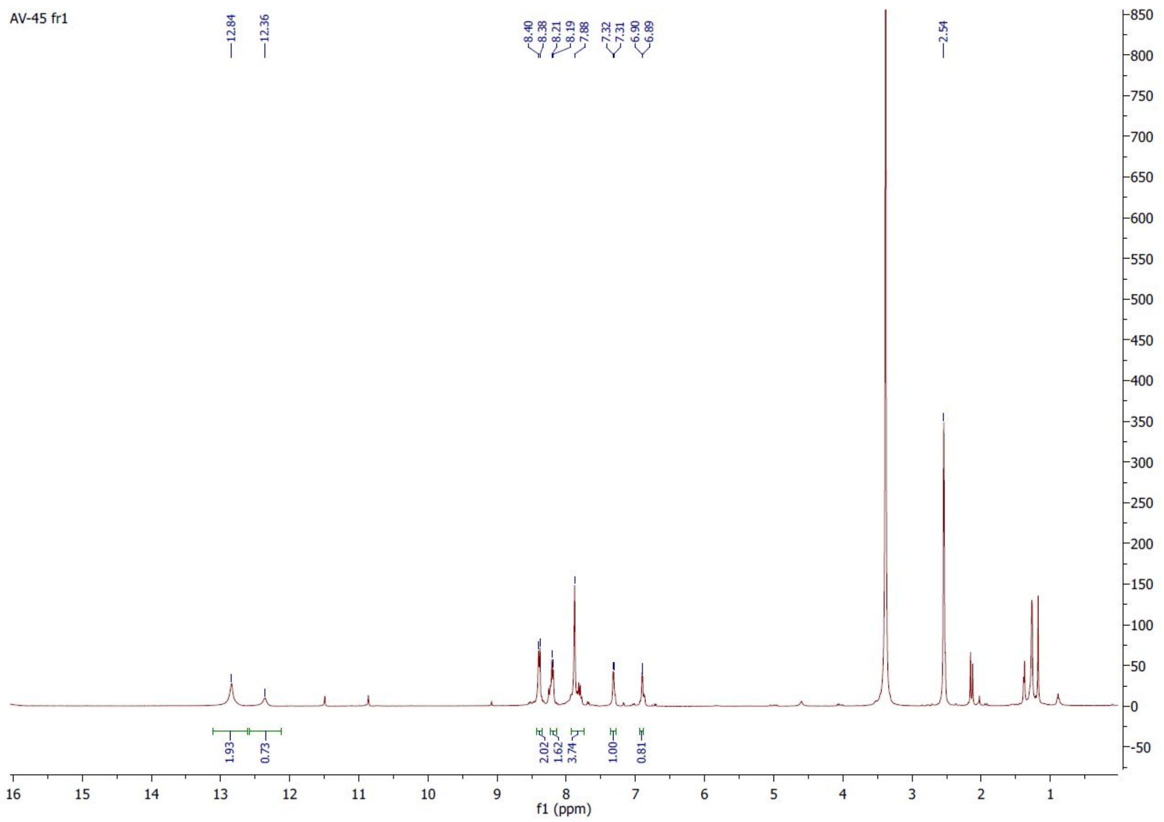

^{1}H NMR spectrum of compound **10d** (400 MHz, DMSO-d_6)

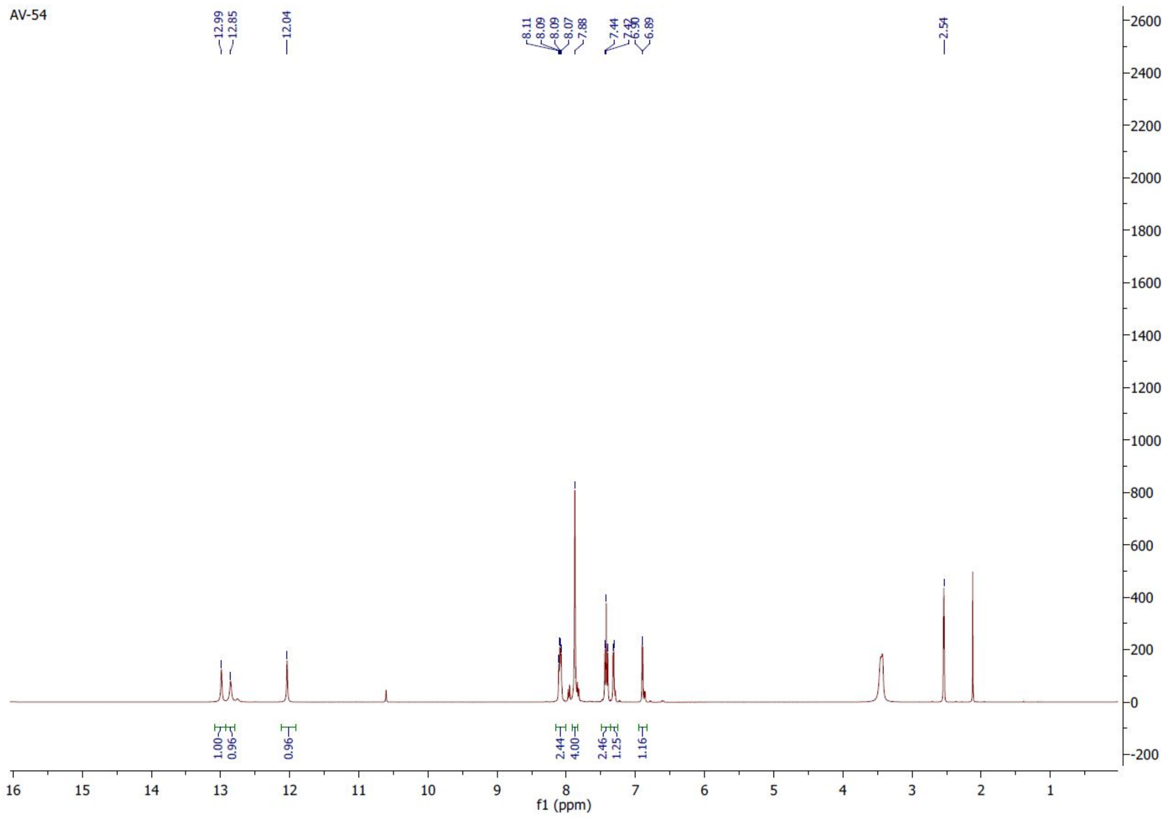

^{1}H NMR spectrum of compound **10e** (400 MHz, DMSO-d_6)

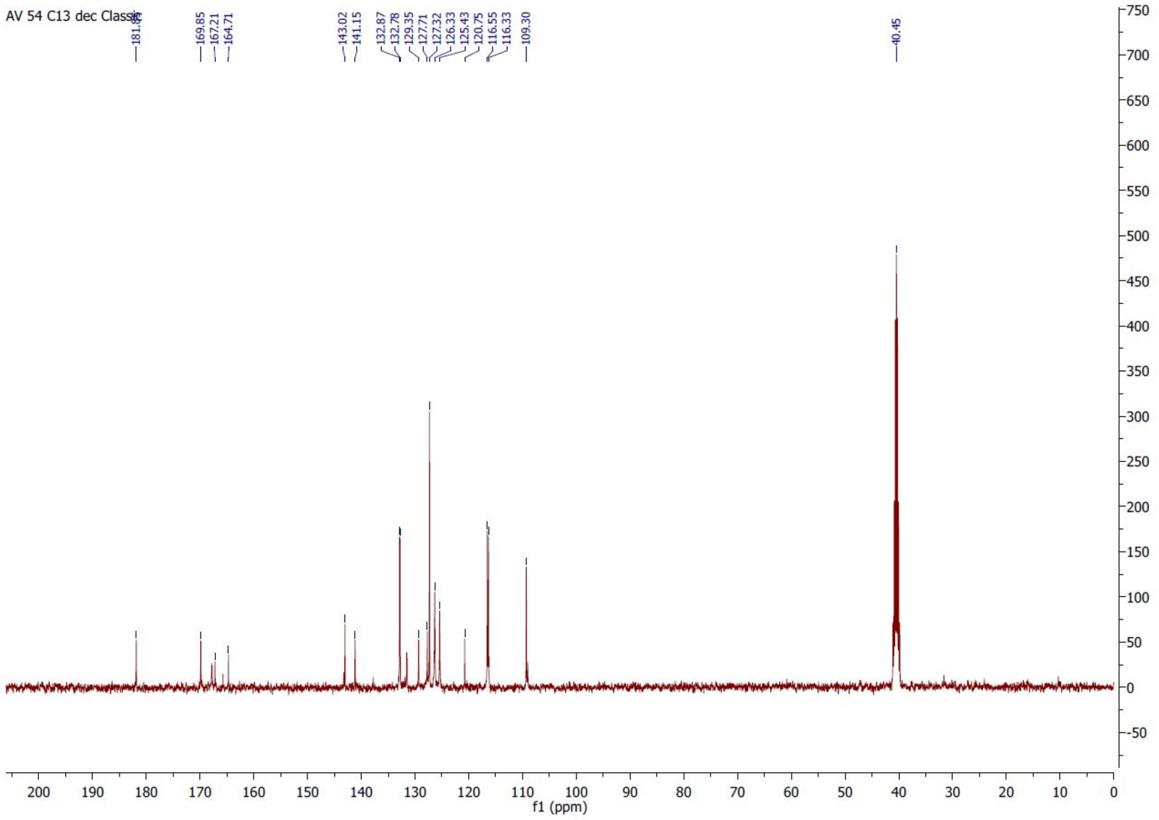

^{13}C NMR spectrum of compound **10e** (100 MHz, DMSO-d_6)

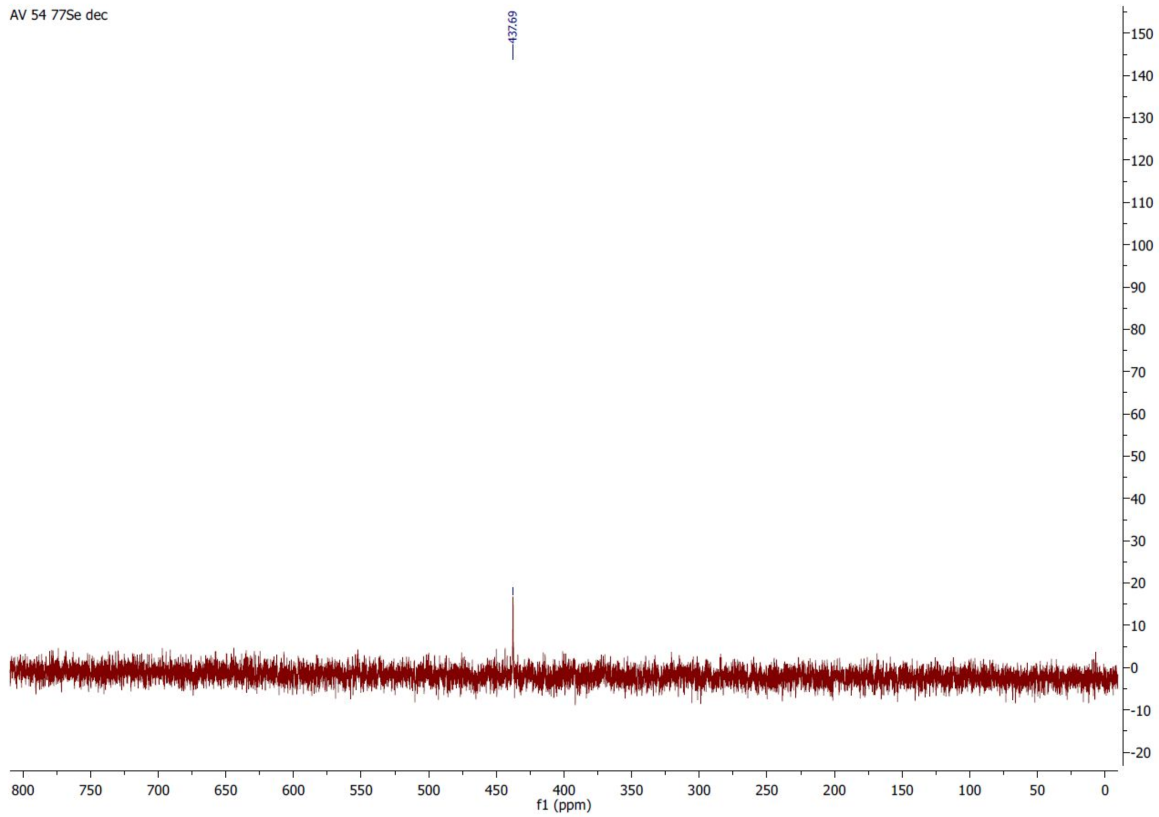

^{77}Se NMR spectrum of compound **10e** (76 MHz, DMSO-d_6)

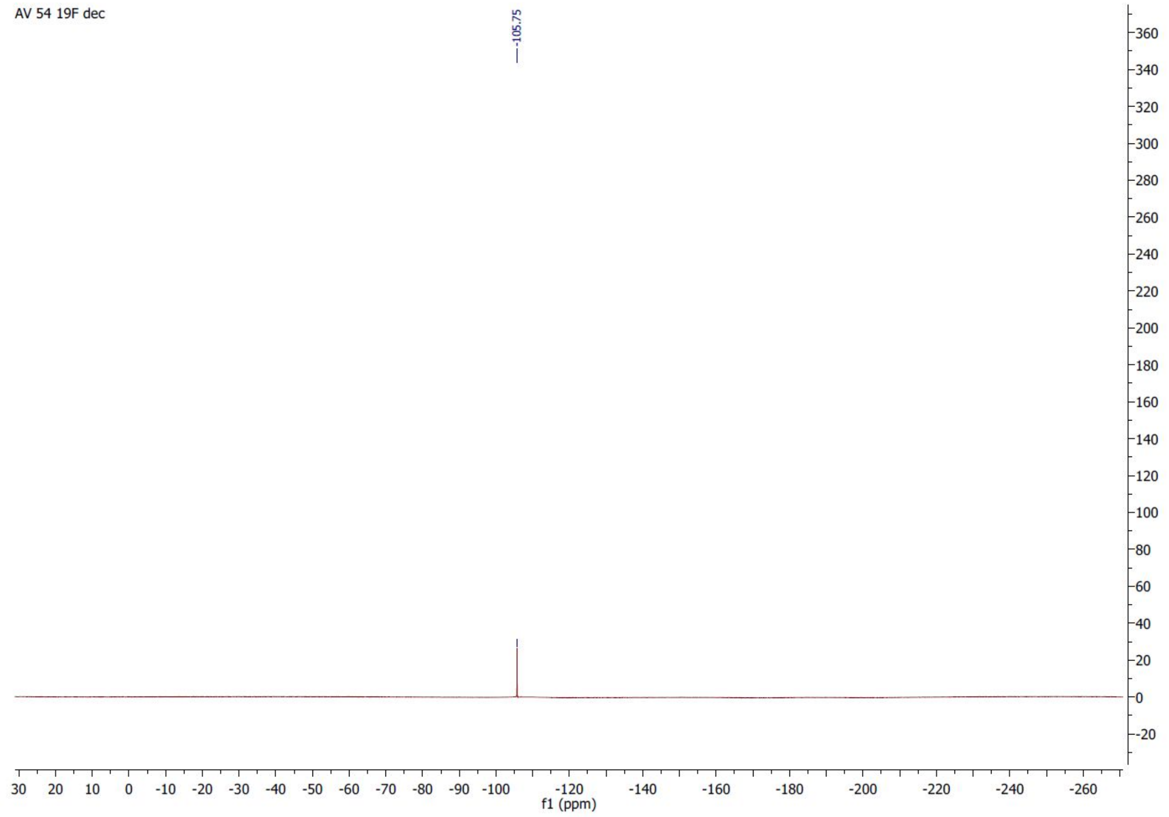

^{19}F NMR spectrum of compound **10e** (376 MHz, DMSO-d_6)

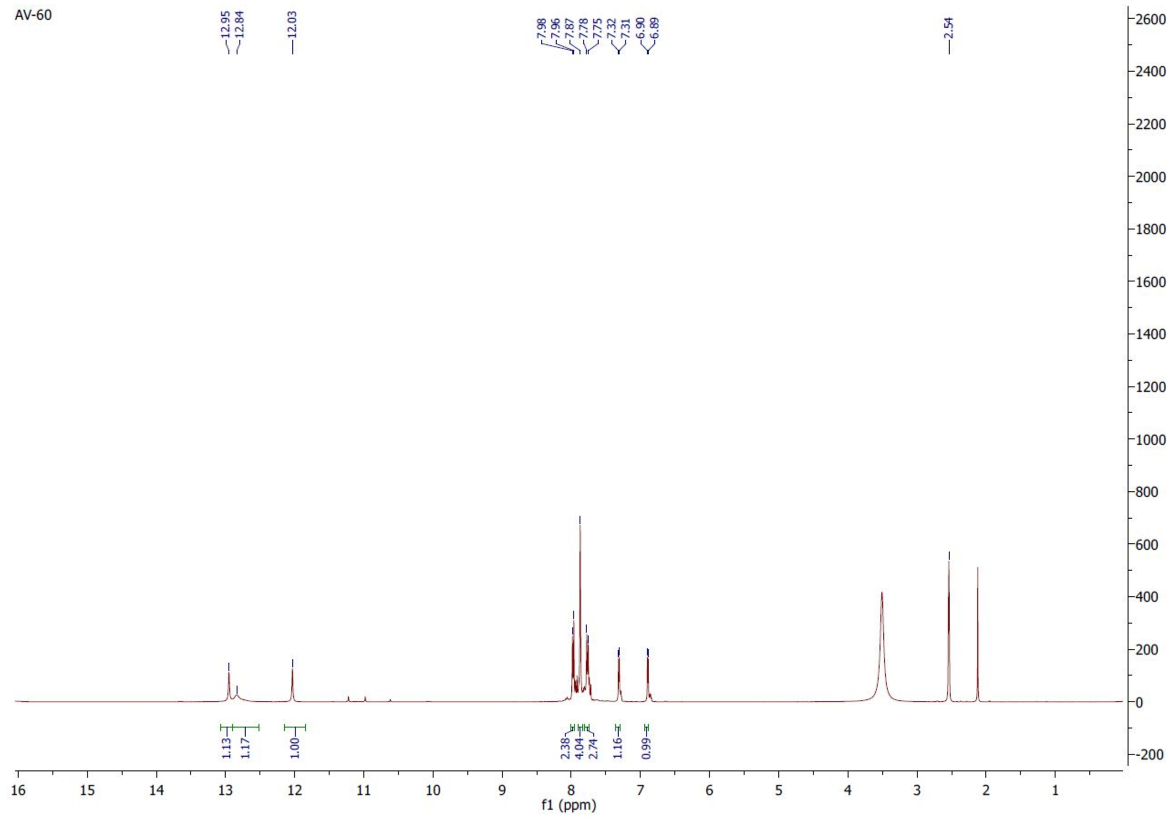

^{1}H NMR spectrum of compound **10f** (400 MHz, DMSO-d_6)

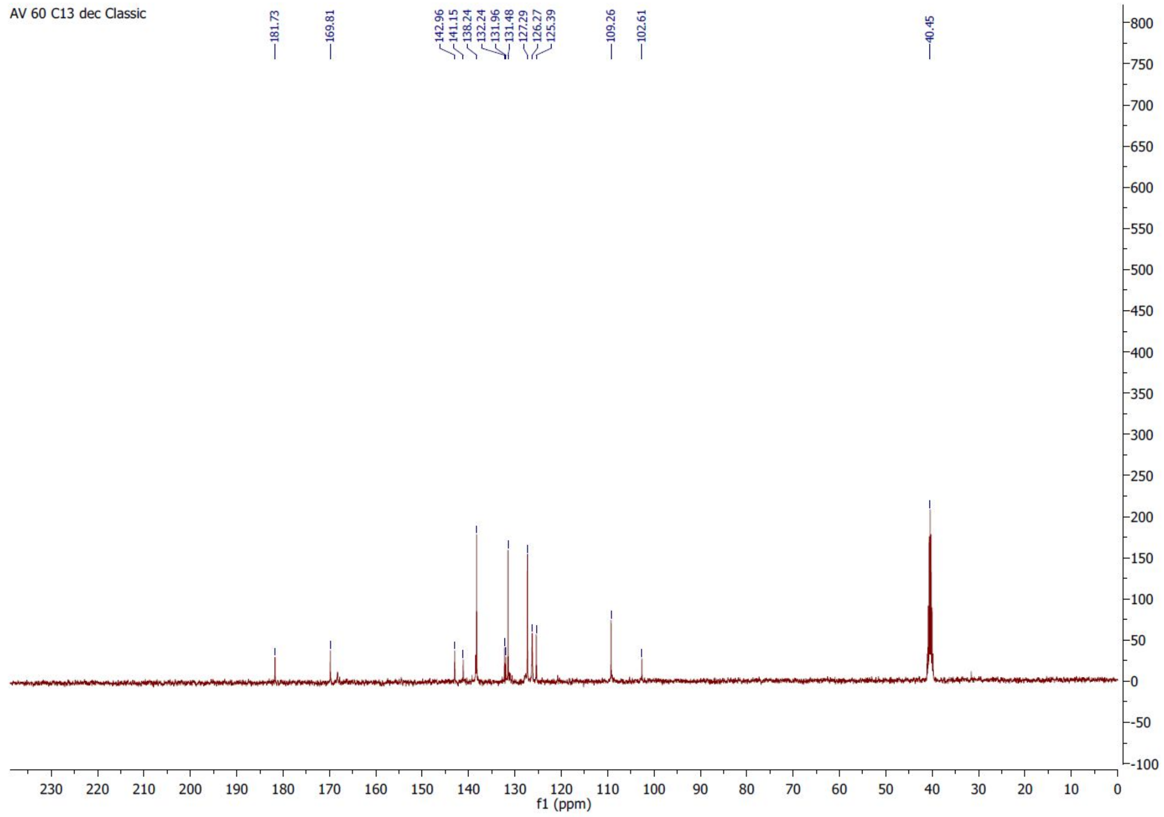

^{13}C NMR spectrum of compound **10f** (100 MHz, DMSO-d_6)

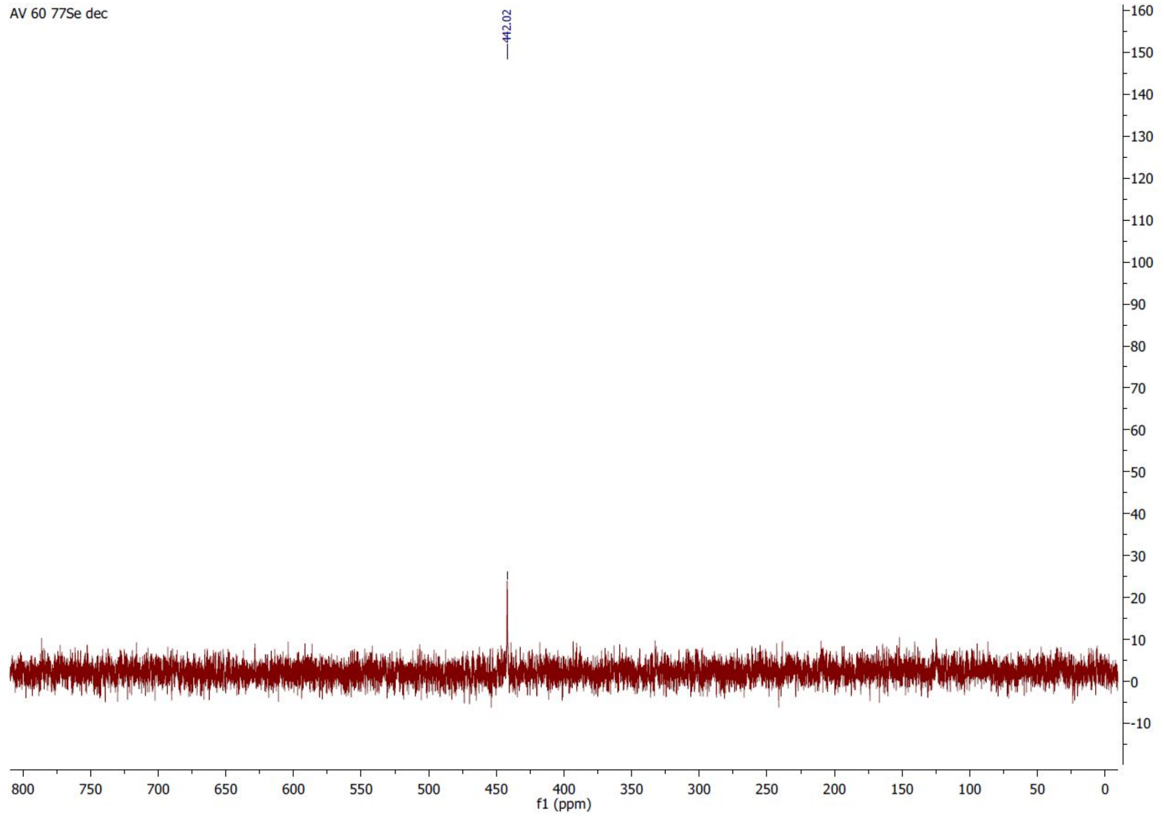

^{77}Se NMR spectrum of compound **10f** (76 MHz, DMSO-d_6)

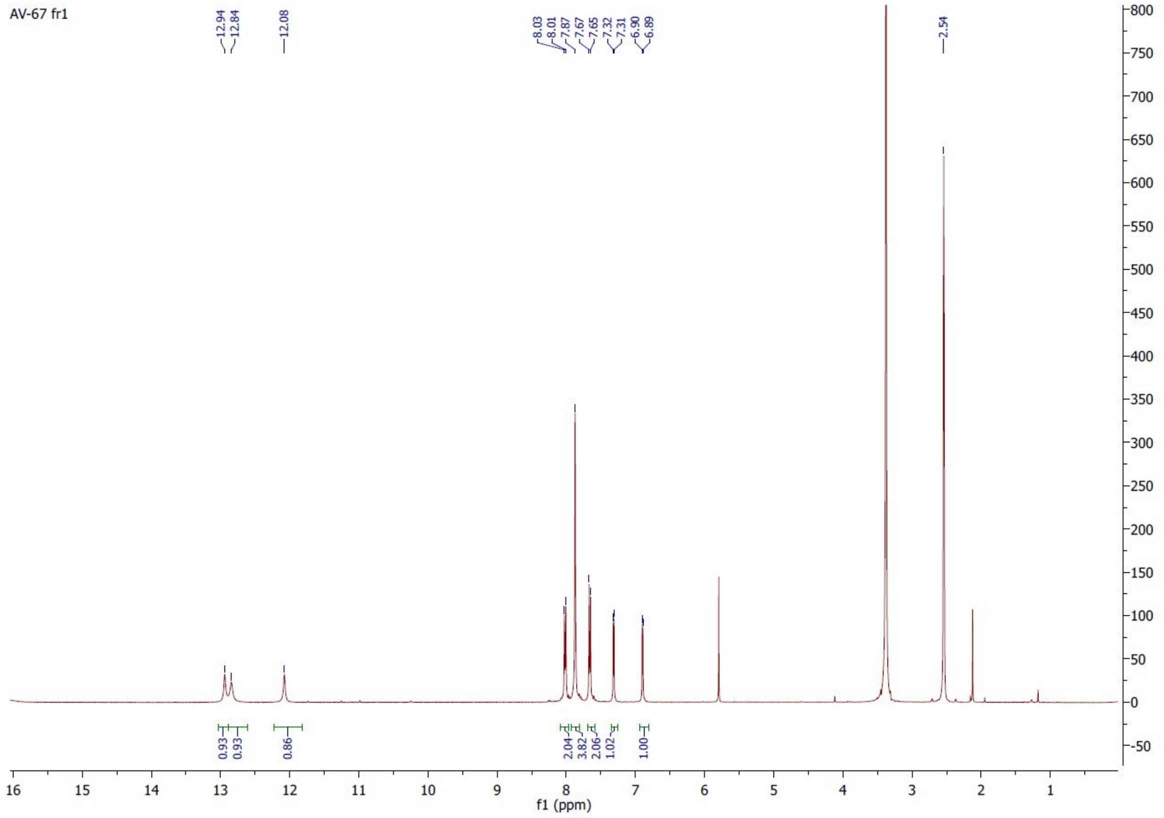

^{1}H NMR spectrum of compound **10g** (400 MHz, DMSO-d_6)

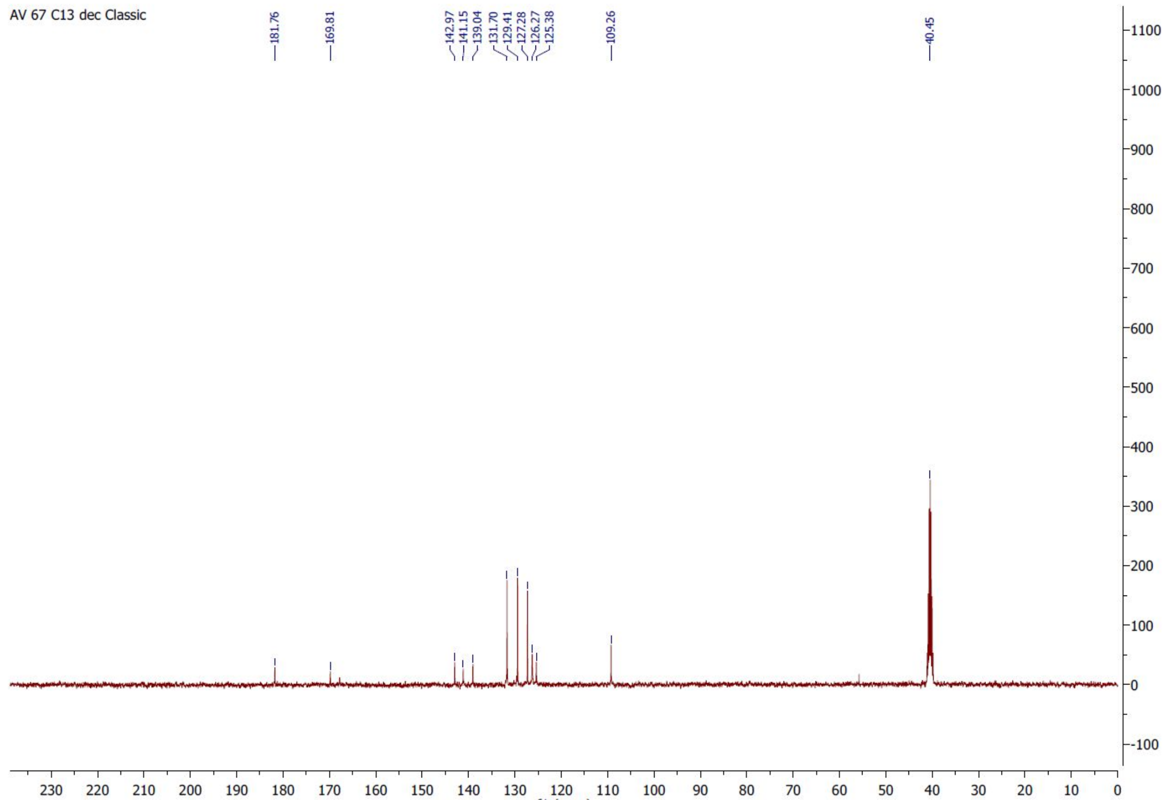

^{13}C NMR spectrum of compound **10g** (100 MHz, DMSO-d_6)

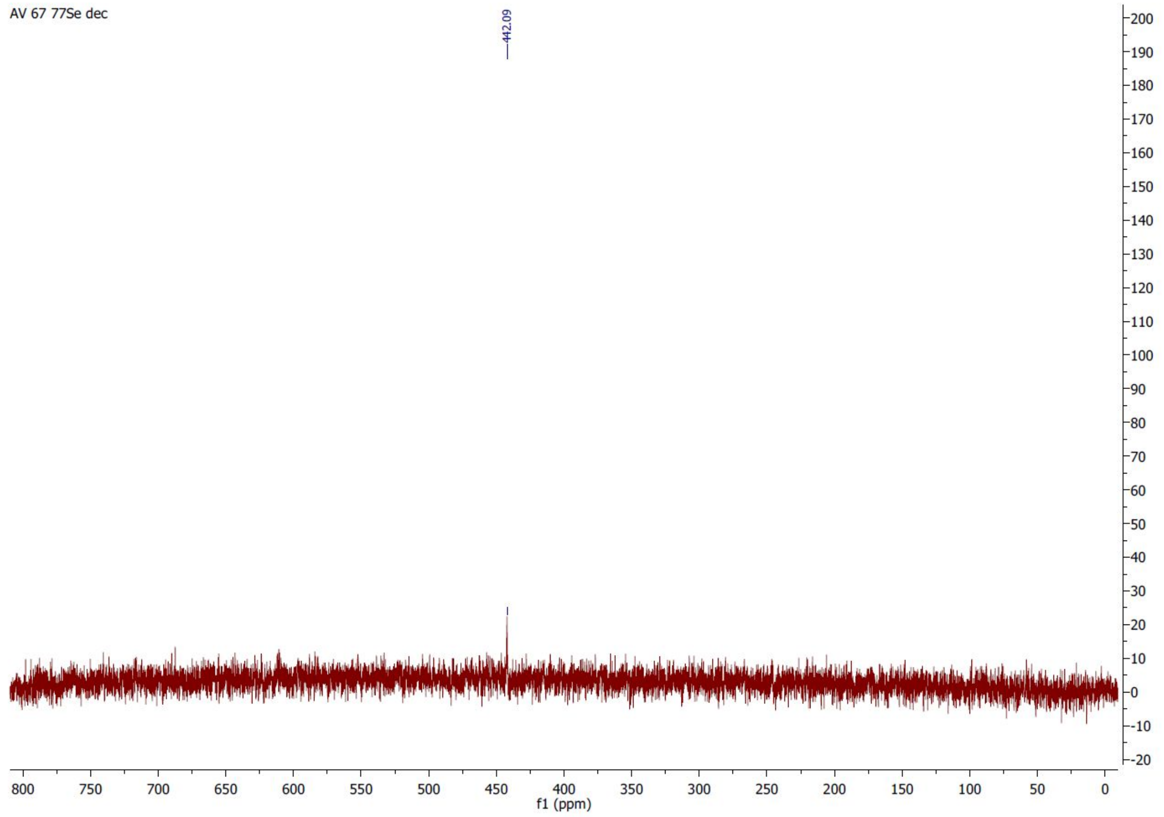

^{77}Se NMR spectrum of compound **10g** (76 MHz, DMSO-d_6)

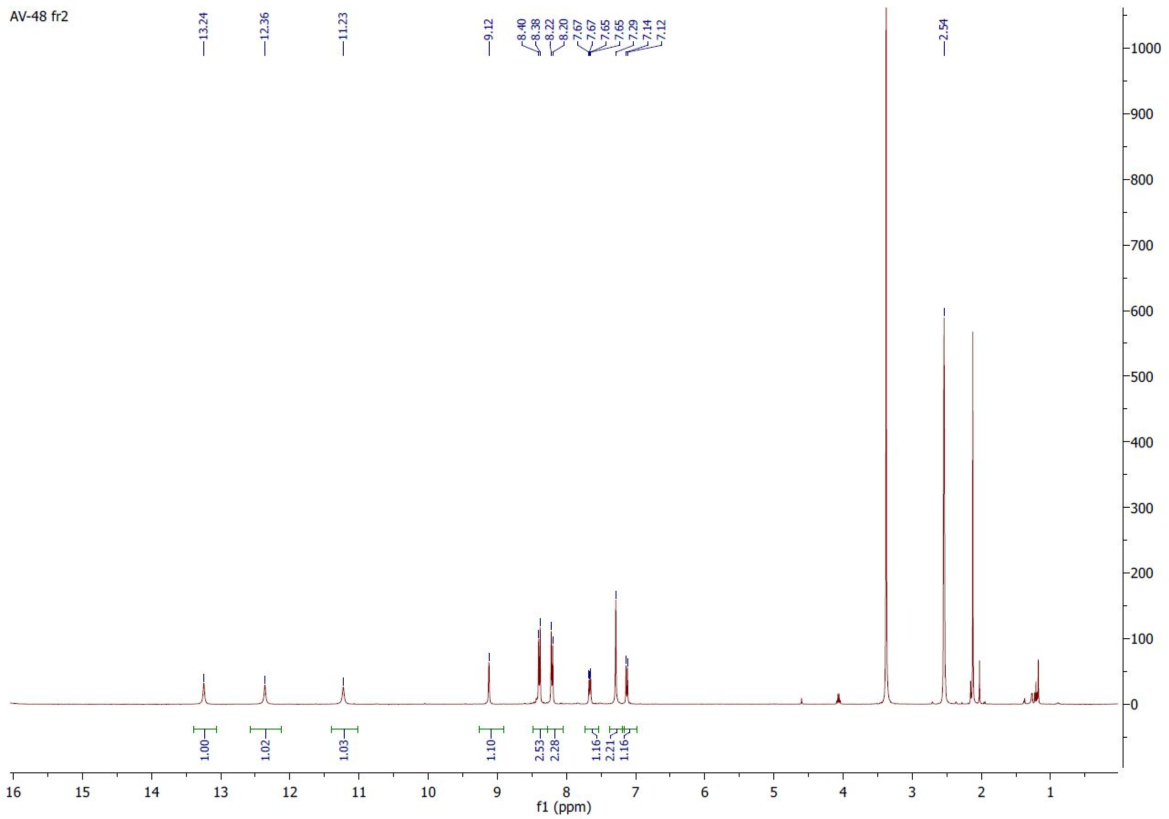

^{1}H NMR spectrum of compound **11c** (400 MHz, DMSO-d_6)

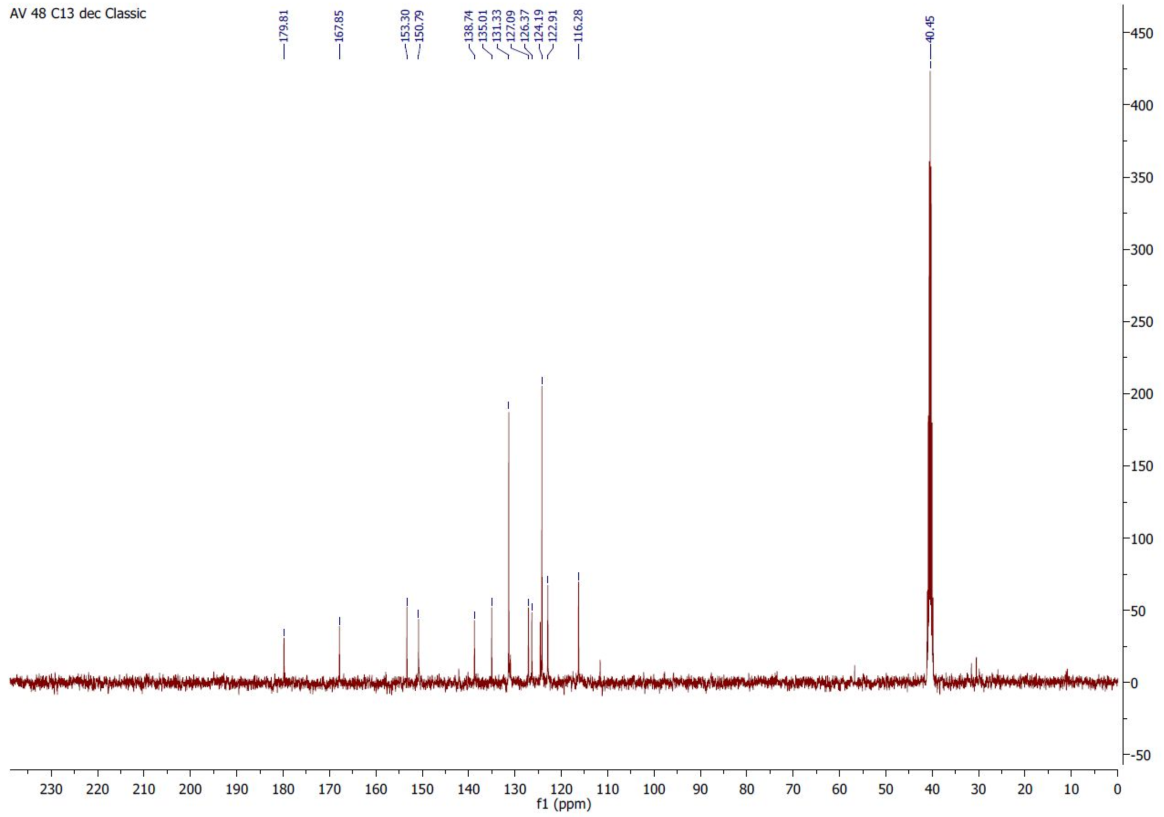

^{13}C NMR spectrum of compound **11c** (100 MHz, DMSO-d_6)

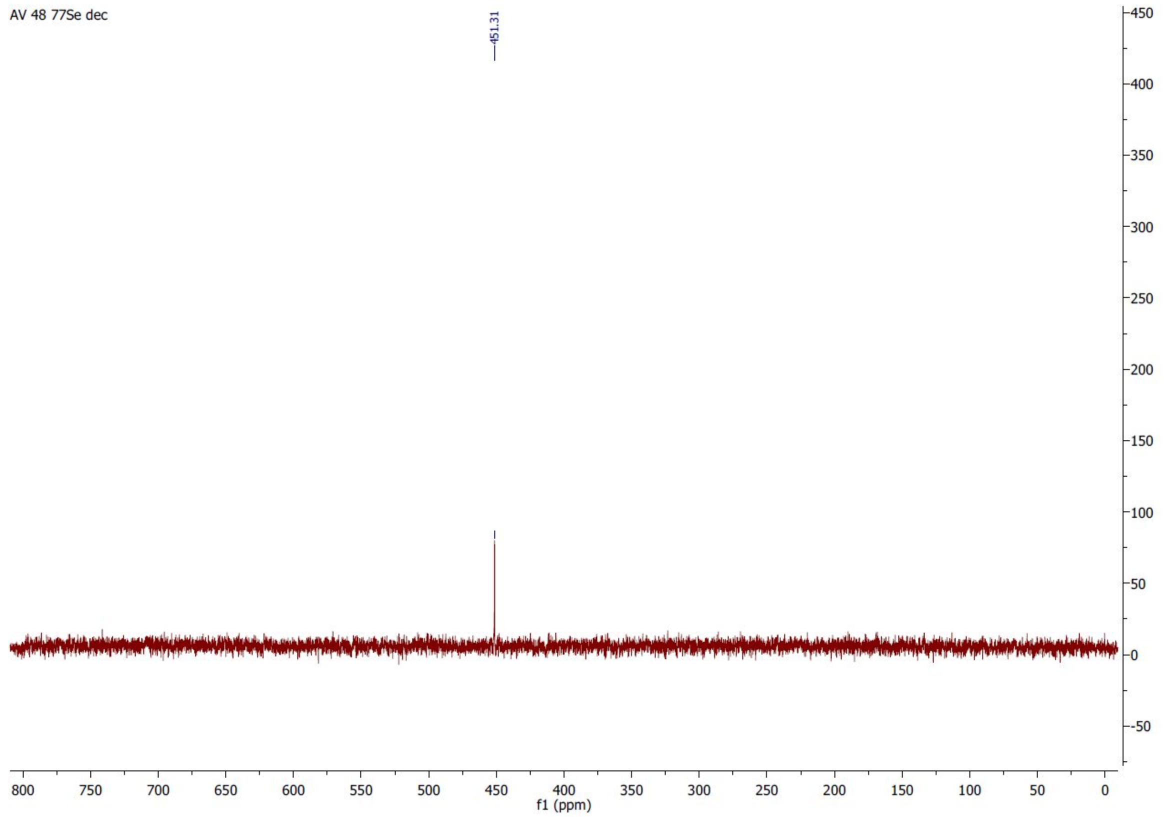

^{77}Se NMR spectrum of compound **11c** (76 MHz, DMSO-d_6)

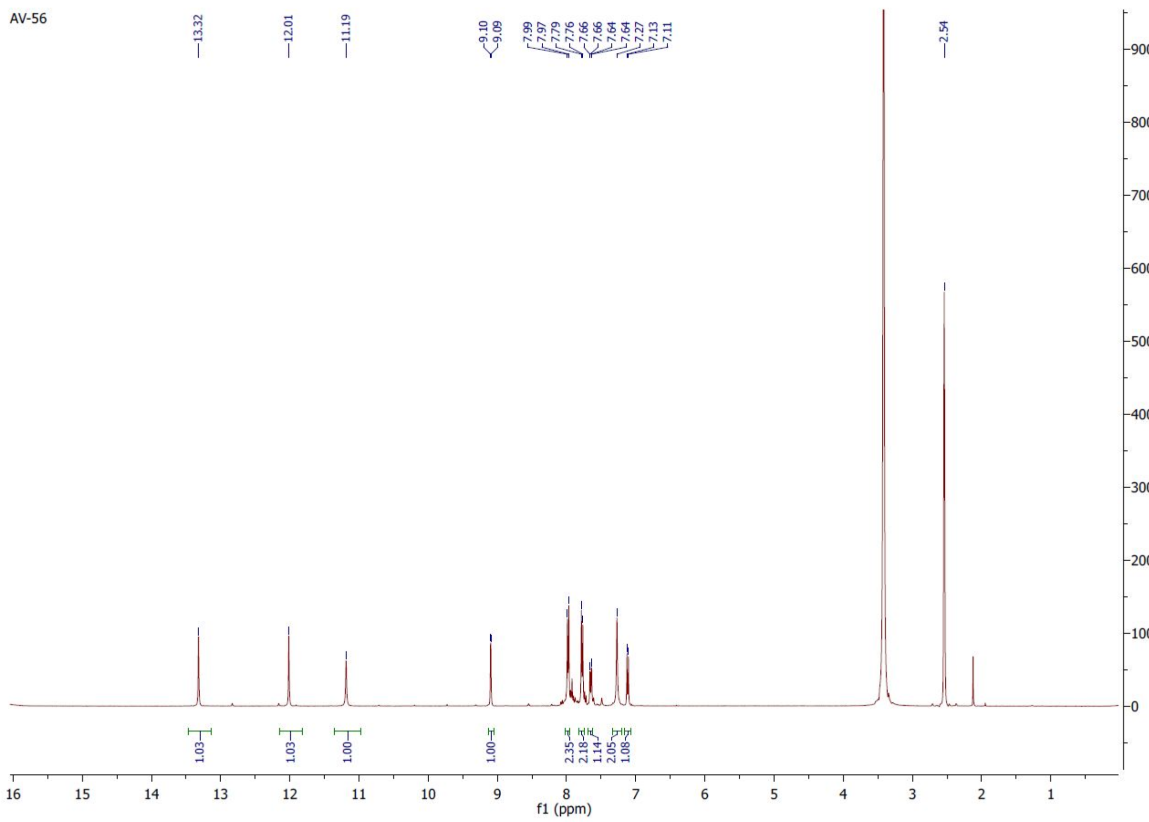

^{1}H NMR spectrum of compound **11d** (400 MHz, DMSO-d_6)

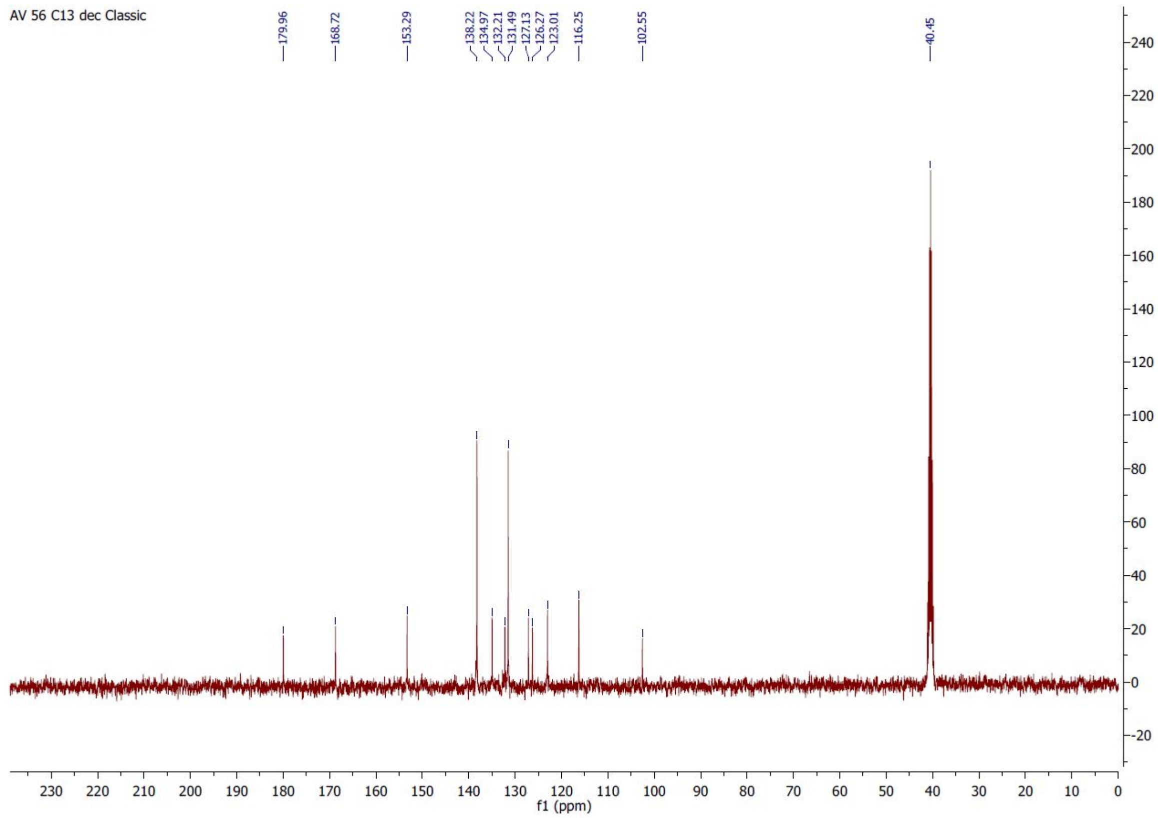

^{13}C NMR spectrum of compound **11d** (100 MHz, DMSO-d_6)

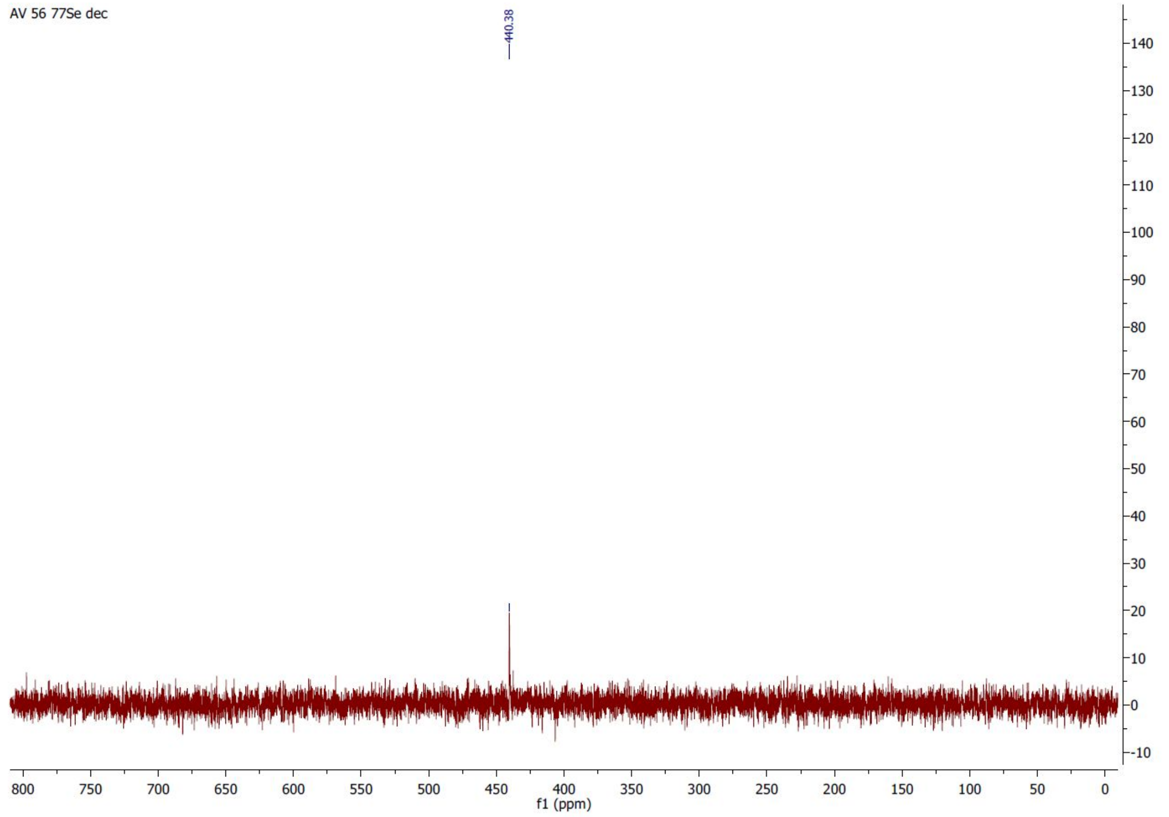

^{77}Se NMR spectrum of compound **11d** (76 MHz, DMSO-d_6)

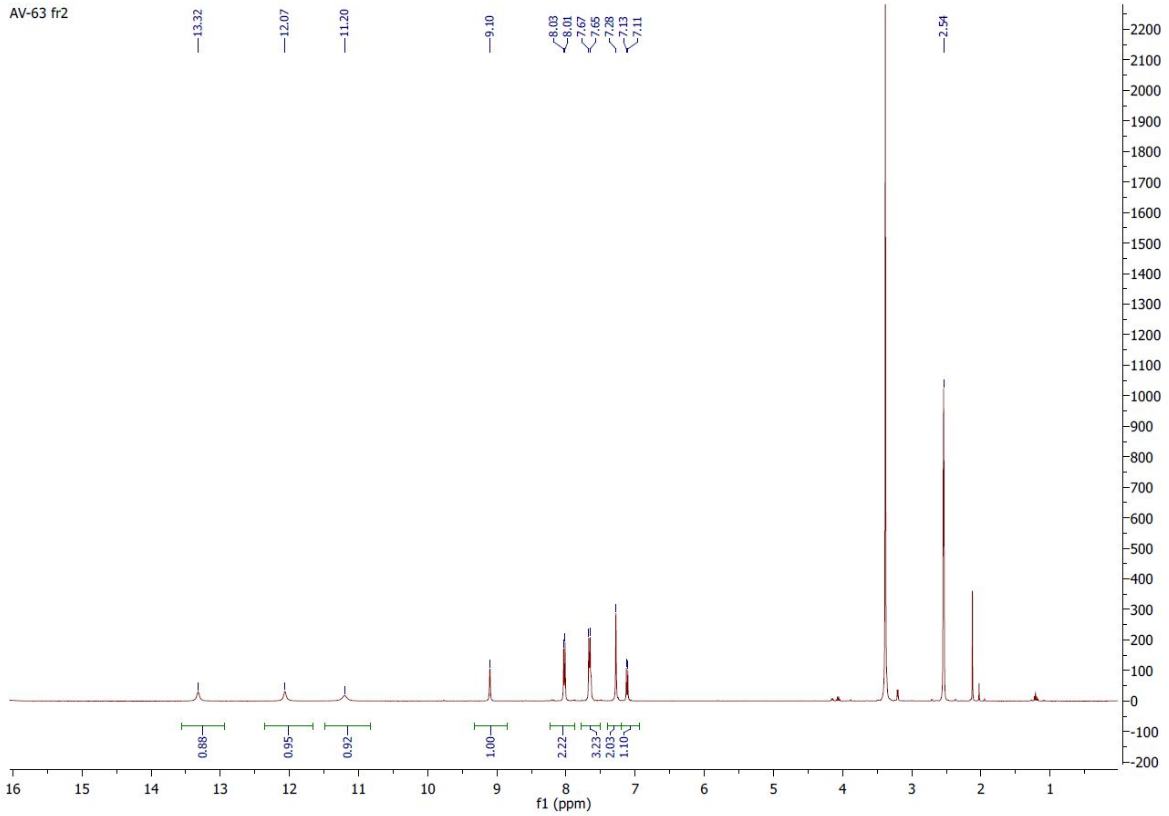

^{1}H NMR spectrum of compound **11f** (400 MHz, DMSO-d_6)

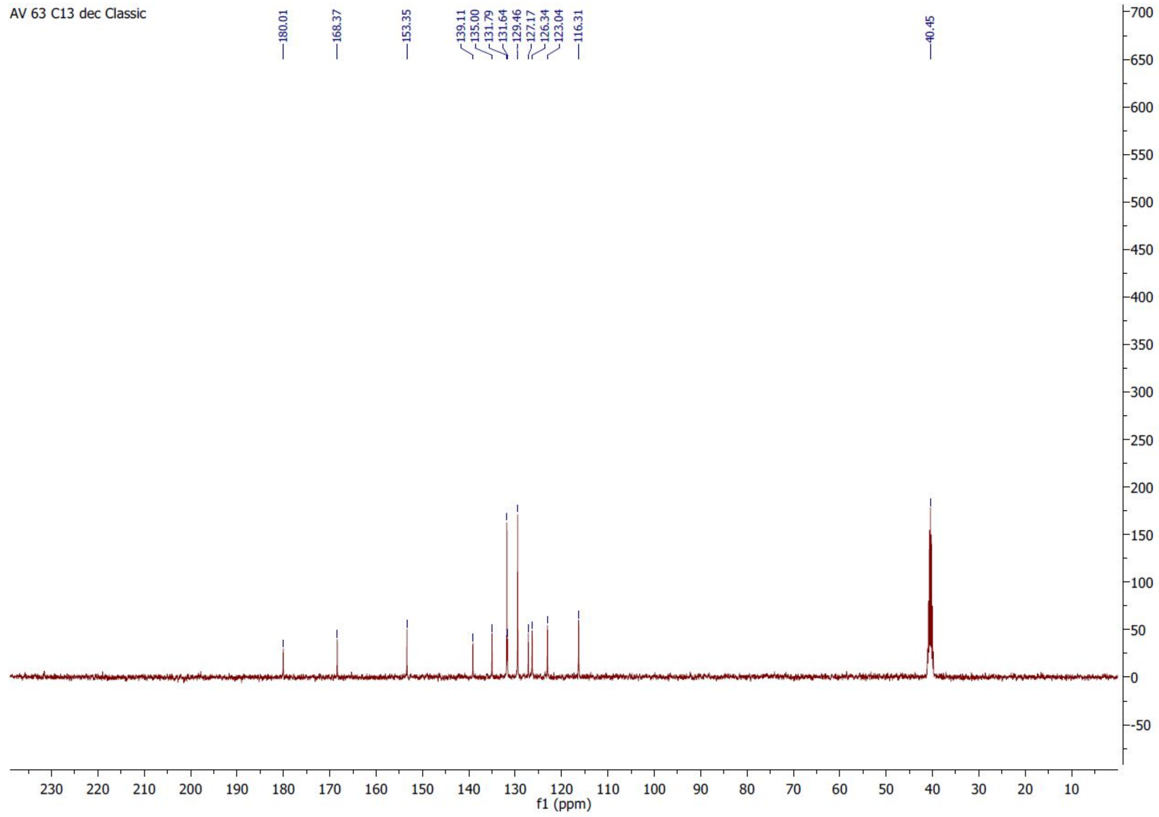

^{13}C NMR spectrum of compound **11f** (100 MHz, DMSO-d_6)

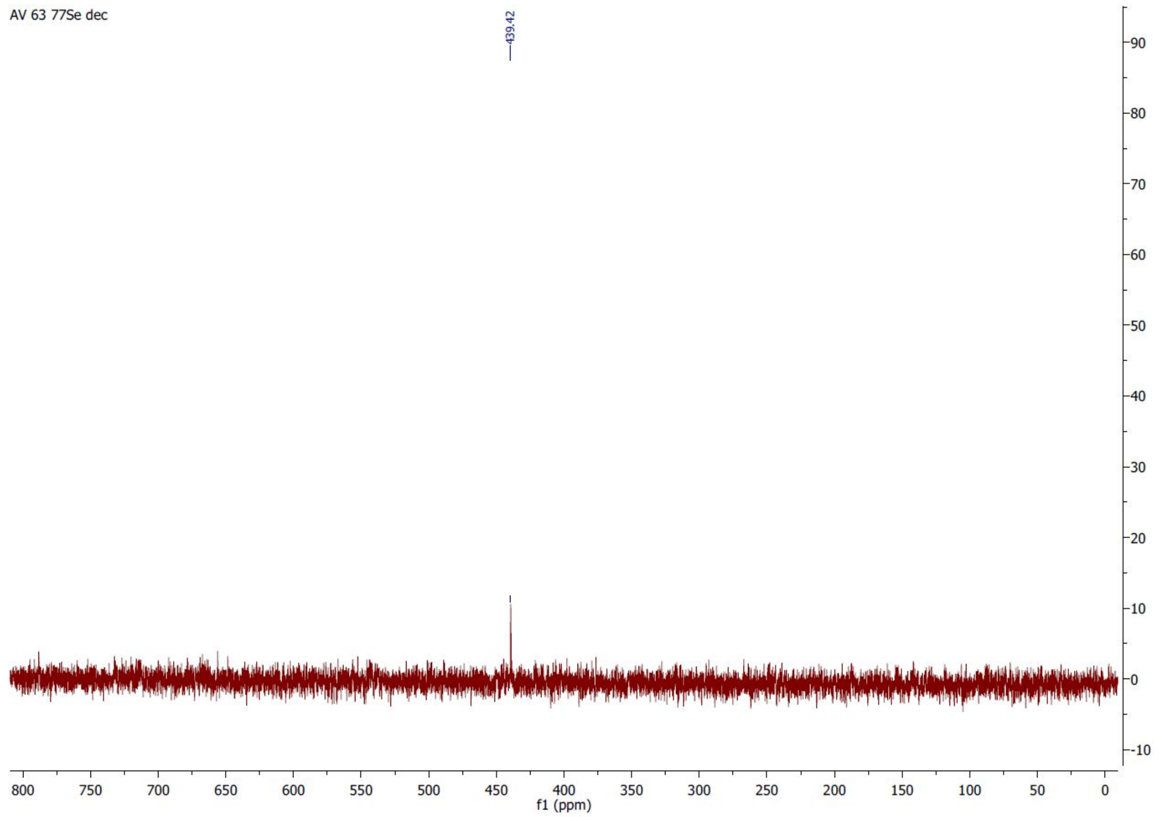

^{77}Se NMR spectrum of compound **11f** (76 MHz, DMSO-d_6)

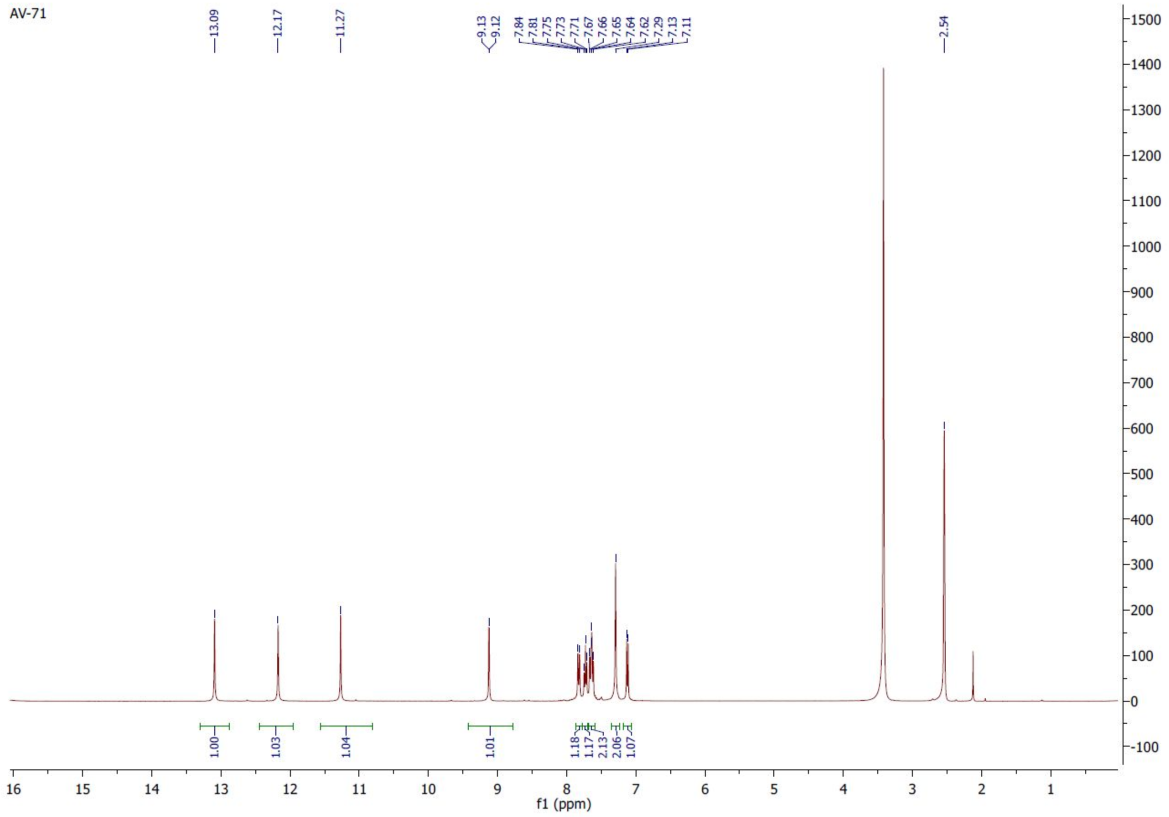

^{1}H NMR spectrum of compound **11g** (400 MHz, DMSO-d_6)

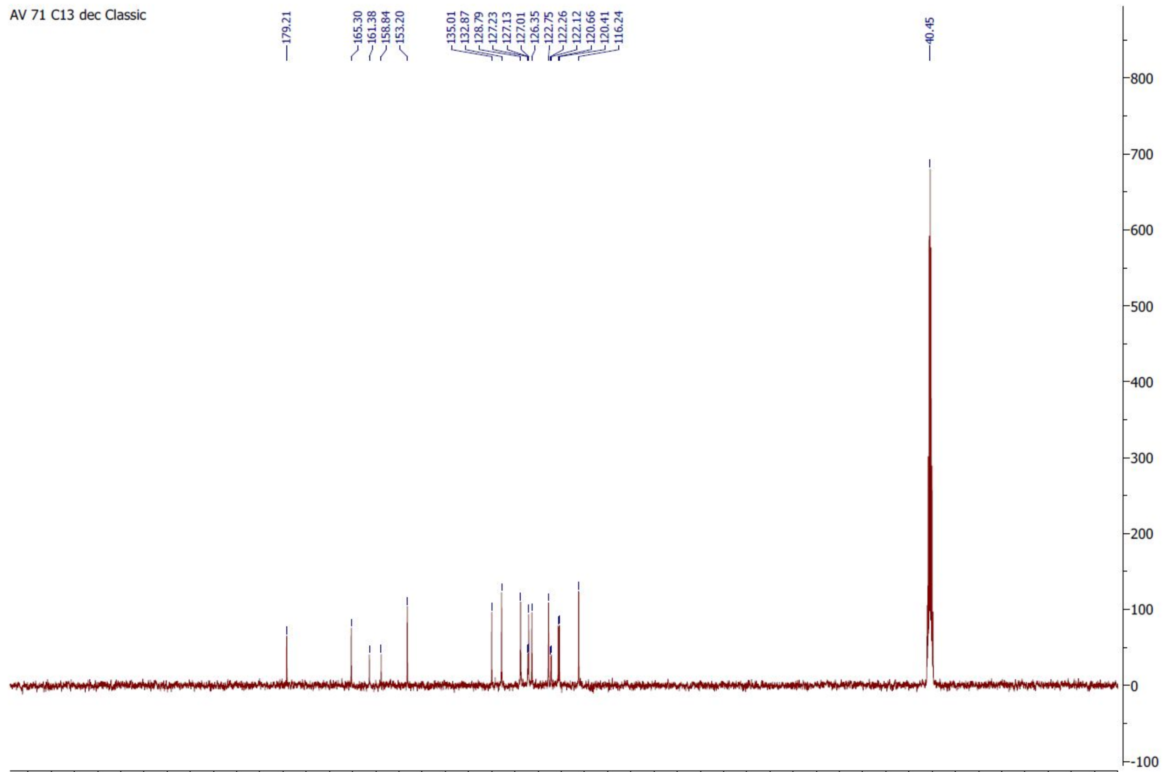

^{13}C NMR spectrum of compound **11g** (100 MHz, DMSO-d_6)

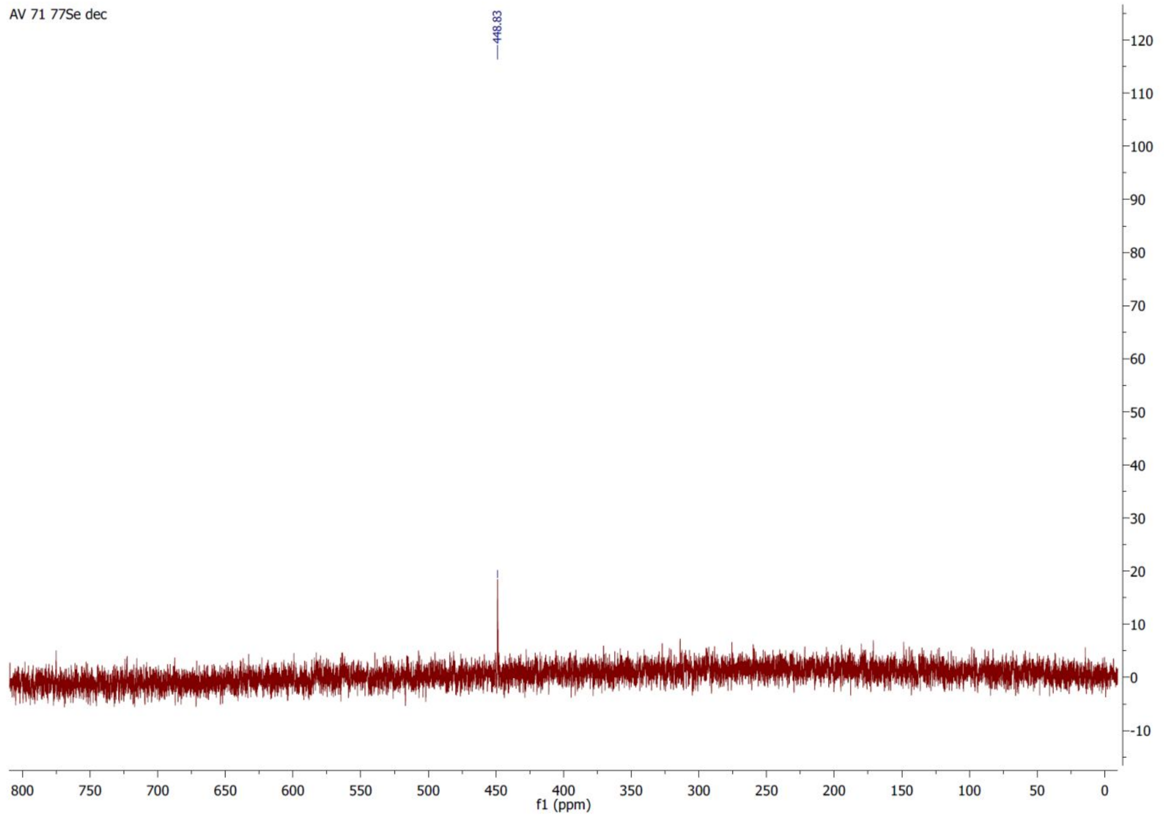

^{77}Se NMR spectrum of compound **11g** (76 MHz, DMSO-d_6)

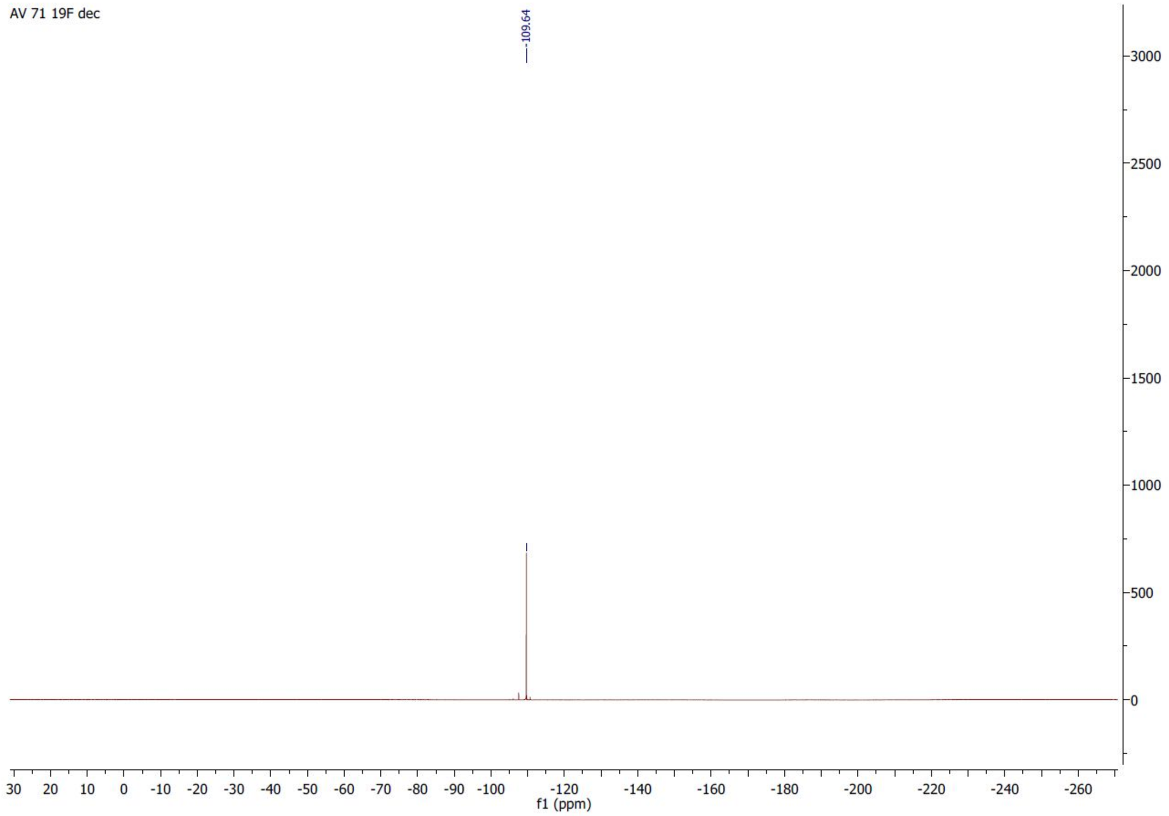

^{19}F NMR spectrum of compound **11g** (376 MHz, DMSO-d_6)

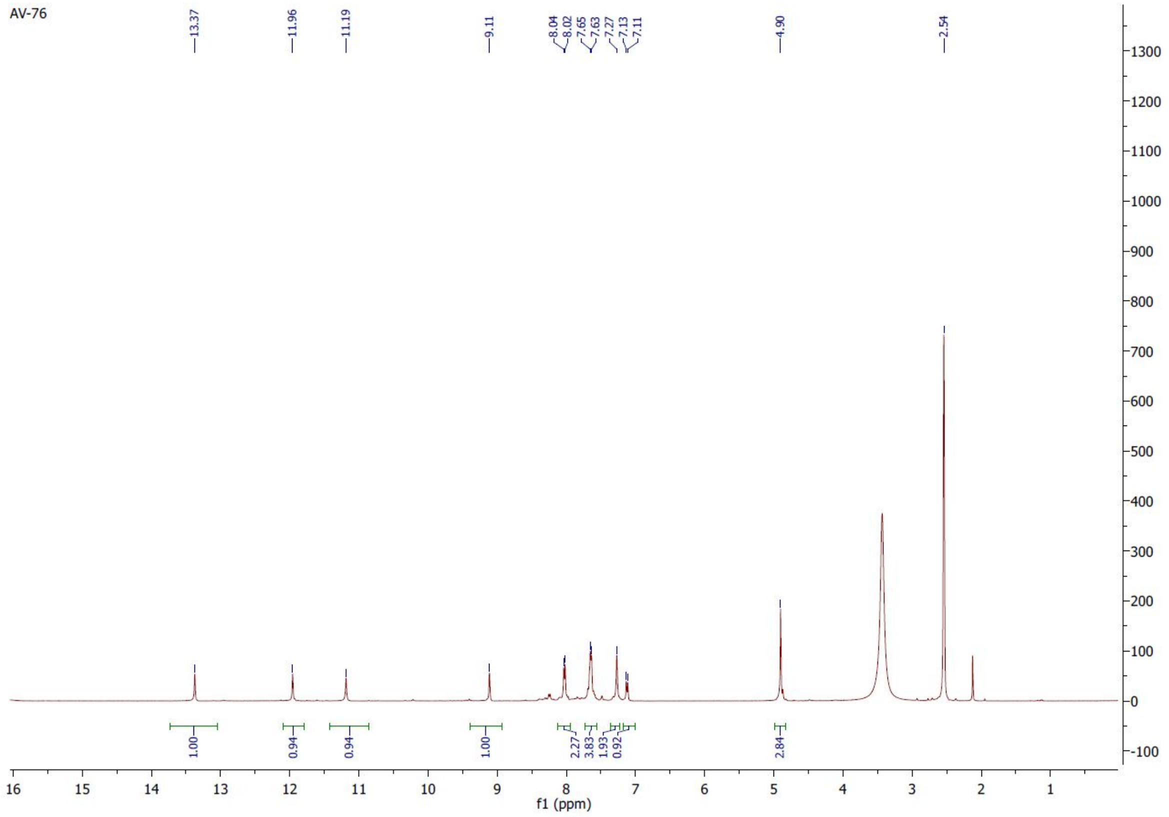

^{1}H NMR spectrum of compound **11h** (400 MHz, DMSO-d_6)

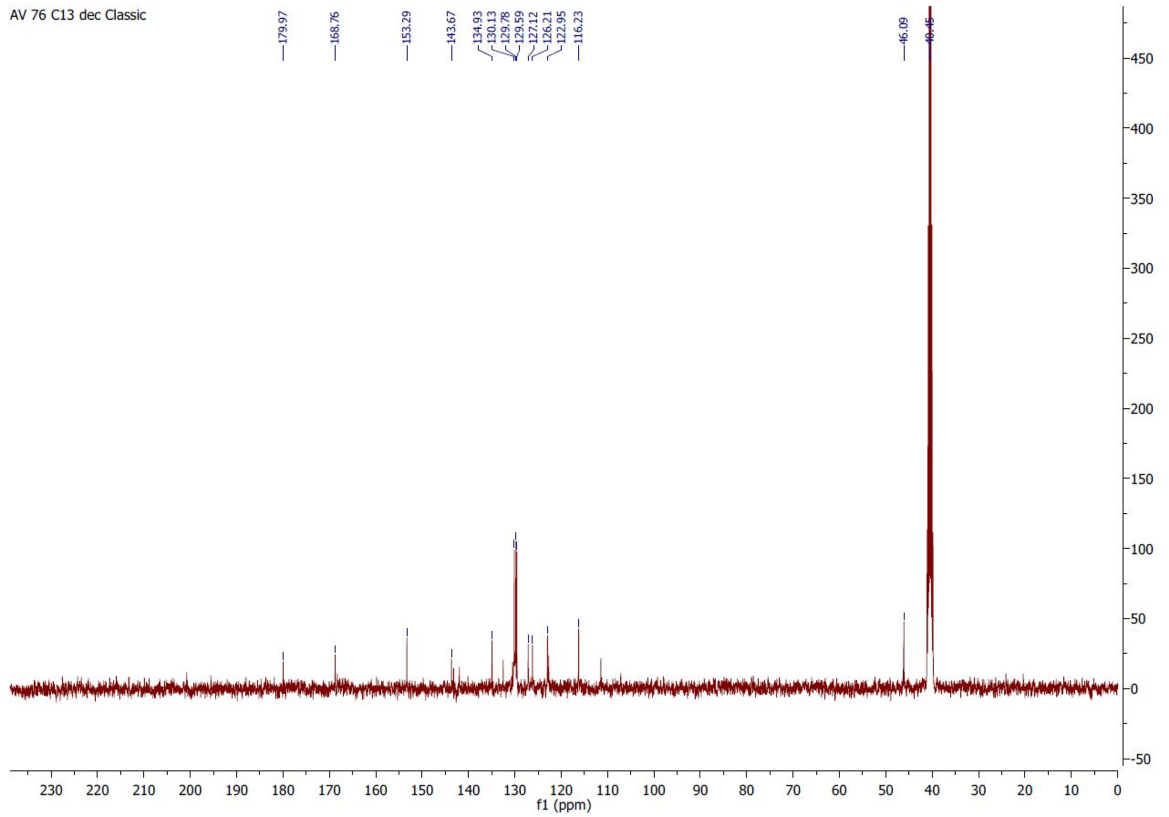

^{13}C NMR spectrum of compound **11h** (100 MHz, DMSO-d_6)

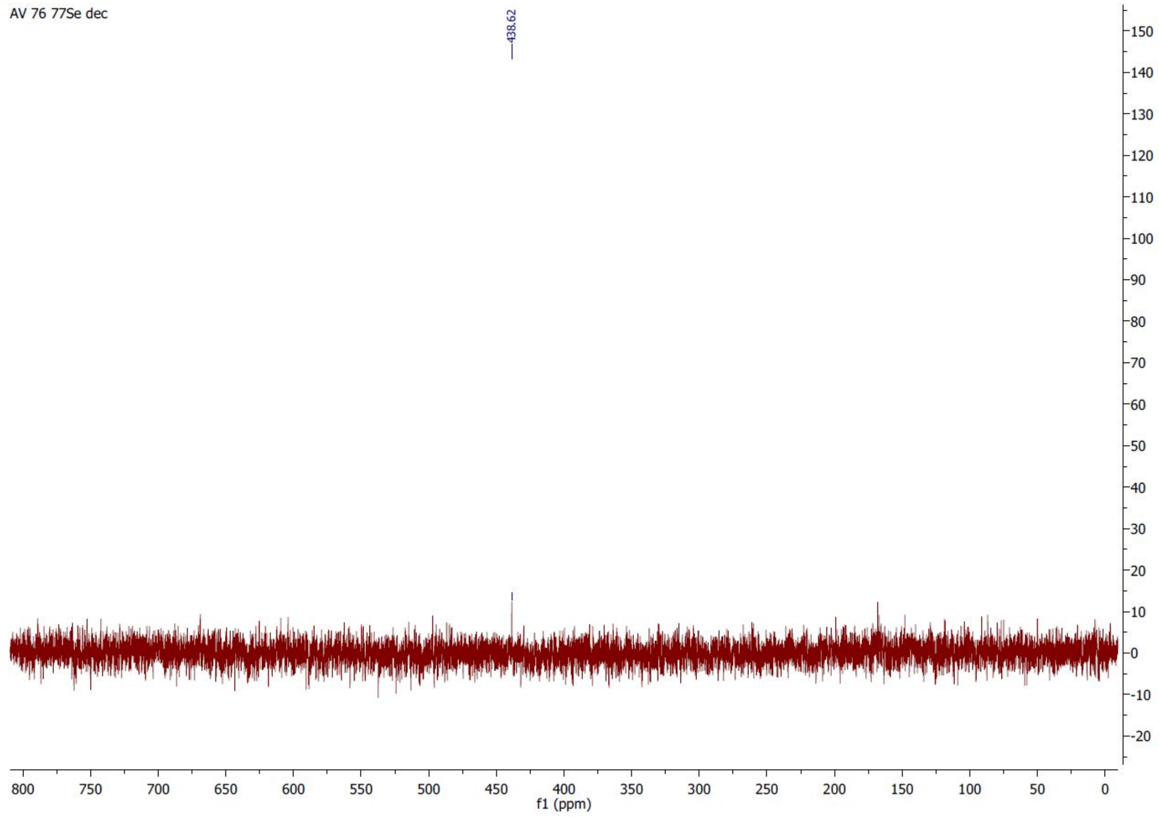

^{77}Se NMR spectrum of compound **11h** (76 MHz, DMSO-d_6)

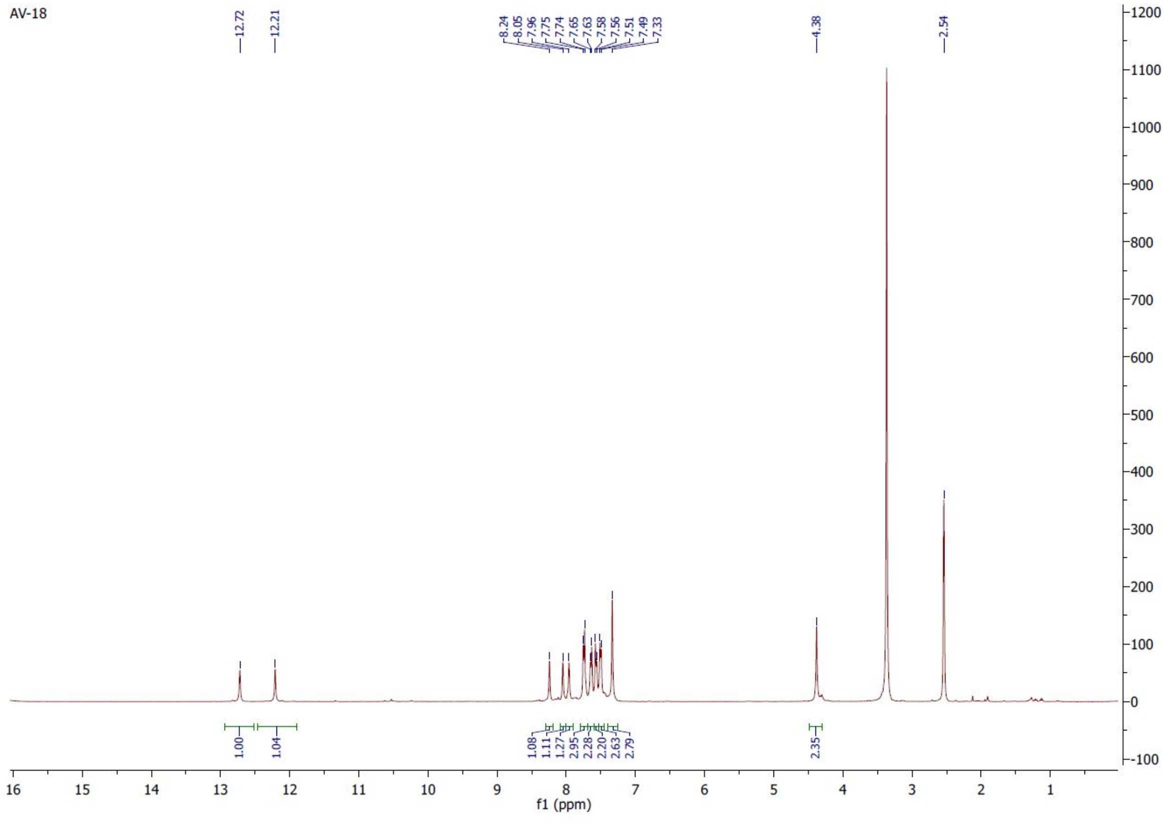

^{1}H NMR spectrum of compound **12** (400 MHz, DMSO-d_6)

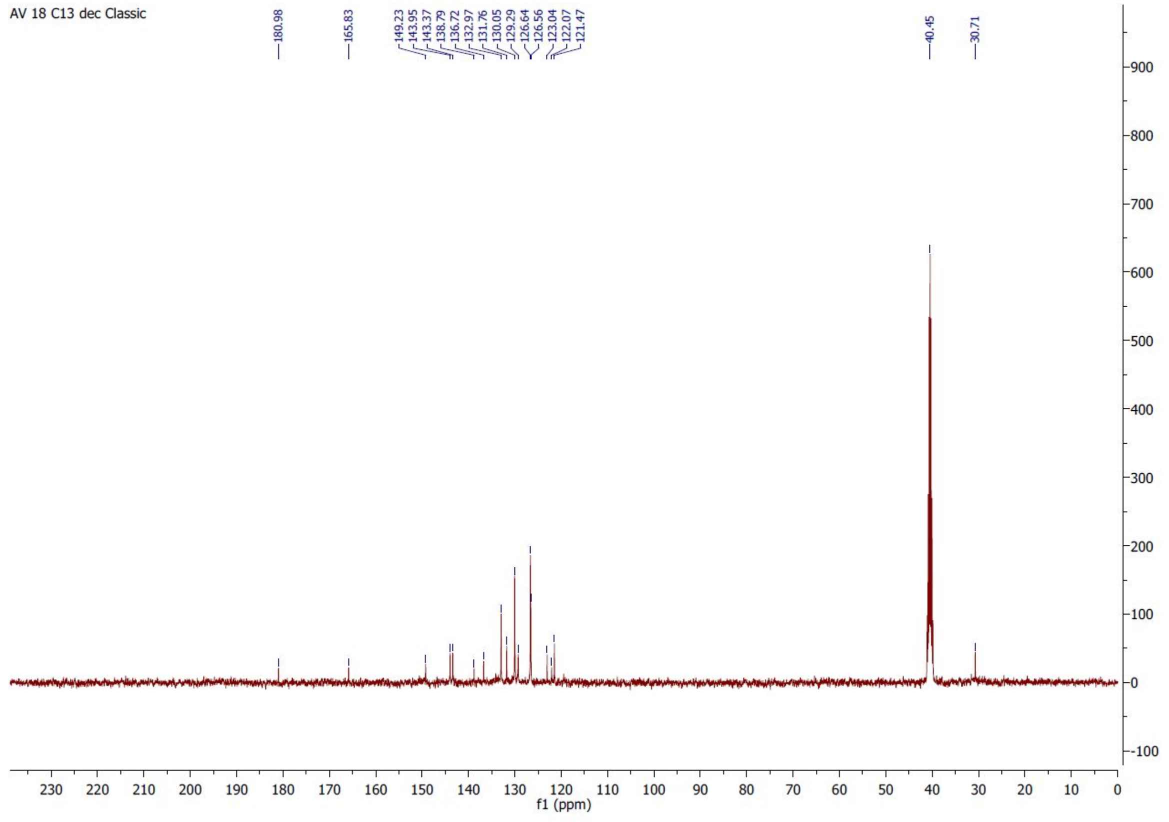

^{13}C NMR spectrum of compound **12** (100 MHz, DMSO-d_6)

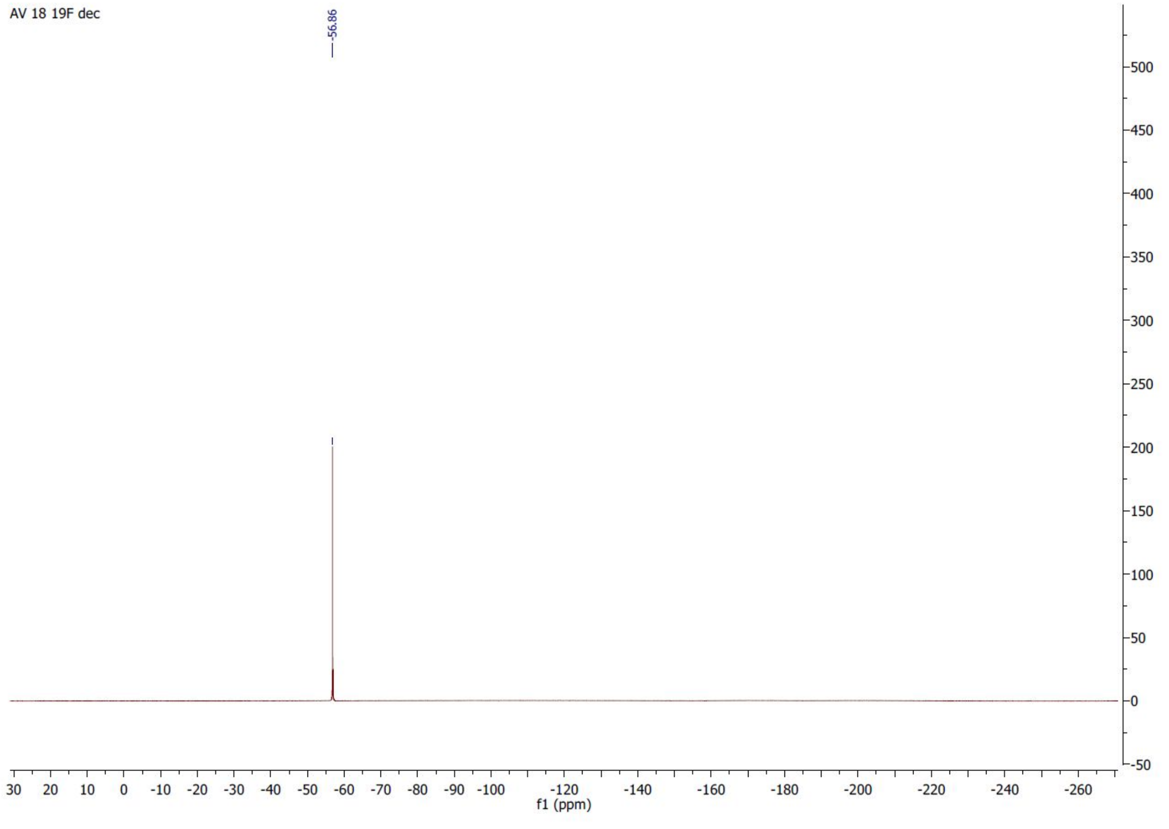

^{19}F NMR spectrum of compound **12** (376 MHz, DMSO-d_6)

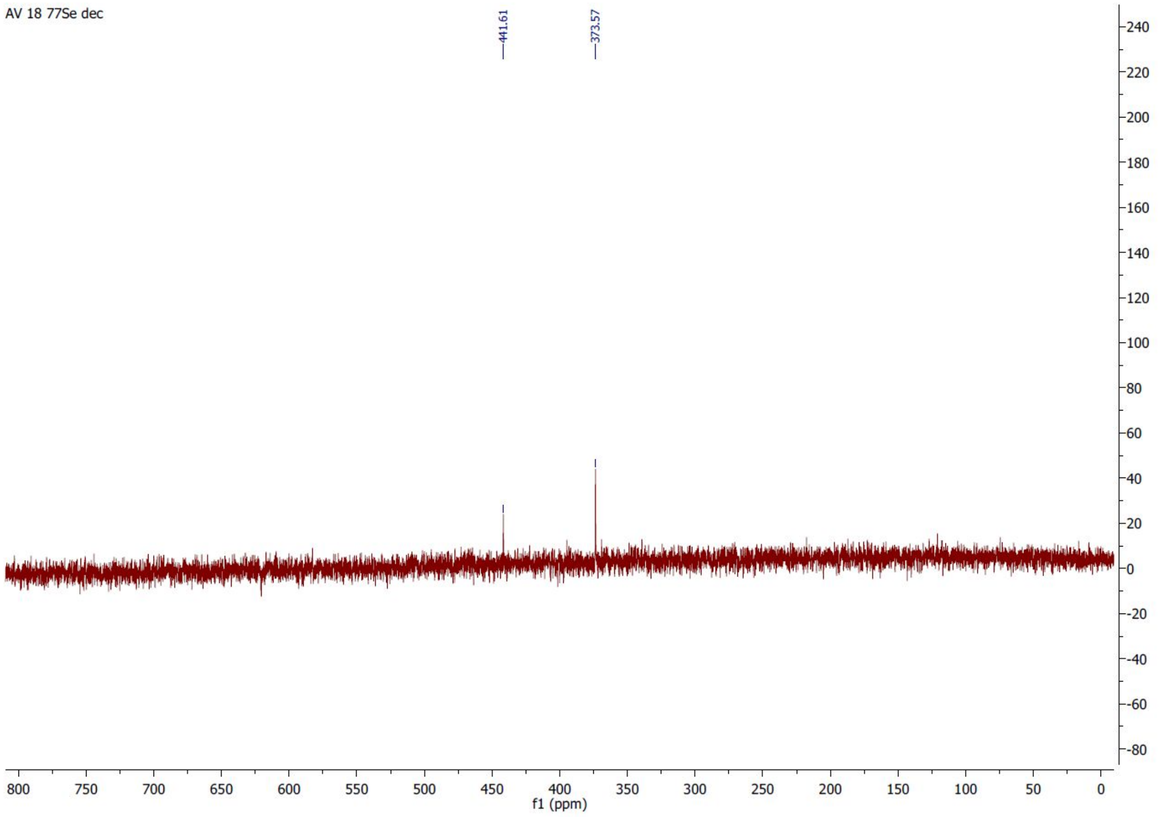

^{77}Se NMR spectrum of compound **12** (76 MHz, DMSO-d_6)

Table S1. SD % values for compounds **3a**, **3c**, **5b**, **7a-d**, **9c**, **11e** and **11i**

**Compound**	% hemolysis	SD %
3a – 256µg/ml	1,21 %	0,25 %
3a – 32 µg/ml	0%	0%
3a – 0,5 µg/ml	0,59%	0,20%
3c - 256 µg/ml	1,85%	0,17%
3c - 64 µg/ml	0%	0%
5b – 256 µg/ml	4,36%	0,78%
5b – 16 µg/ml	0%	0%
5b – 0,5 µg/ml	0%	0%
7a – 256 µg/ml	2,90%	0,79%
7a – 0,5 µg/ml	0,78%	0,17%
7b – 256 µg/ml	1,20%	0,04%
7b – 16 µg/ml	0%	0%
7b – 0,5 µg/ml	0,57%	0,19%
7c - 256 µg/ml	29,77%	4,56%
7c - 16 µg/ml	0%	0%
7c – 0,5 µg/ml	0%	0%
7d – 256 µg/ml	18,96%	1,98%
7d – 0,5 µg/ml	0%	0%
9c – 256 µg/ml	2,31%	0,19%
9c – 1 µg/ml	0%	0%
11c – 256 µg/ml	9,25%	1,48%
11c – 0,5 µg/ml	0%	0%
11i – 256 µg/ml	25,71%	4,22%
11i – 32 µg/ml	0%	0%
11i – 0,5 µg/ml	0,37%	0,09%

Table S2. P-values calculated with T-test of data on Figures 1

Compound	Concentration	Survival %	P value (T-test)	Figure
3e	256	24.00%	< 0.0001 ****	1A
	8	63.68%	0.015 *	
	1	76.63%	0.214	
5b	64	20,00%	< 0.0001 ****	
	0.5	91,53%	0.023 *	
5e	4	42.85%	0.0031 **	
	0.5	76.81%	0.341	
5f	2	97.70%	0.014 *	
	0.5	92.05%	0.115	
5g	32	27.92%	< 0.0001 ****	
	16	25.75%	< 0.0001 ****	
	2	82.48%	0.515	
	1	69.96%	0.0019 **	
5h	64	73.88%	0.010 **	
	2	76.25%	0.054	
	1	77.23%	0.248	
7a	0.5	83.81%	0.0145 *	1B
7b	0.5	62.01%	0.00019 ***	
7c	0.5	94.54%	0.045 *	
7e	16	17.56%	< 0.0001 ****	
	0.5	79.82%	0.701	
7g	8	20.04%	< 0.0001 ****	
	0.5	84.61%	0.454	
7h	64	24.75%	< 0.0001 ****	
	0.5	76.74%	0.063	
7k	256	25.91%	< 0.0001 ****	
	8	75.32%	0.015 *	
	0.5	105.57%	0.008 **	
8b	256	22.72%	< 0.0001 ****	
	8	75.16%	0.024 *	
	1	82.84%	0.488	
8c	256	24.41%	< 0.0001 ****	1C
	8	99.85%	< 0.0001 ****	
	2	81.48%	0.950	
9a	32	50.20%	< 0.0001 ****	
	4	45.52%	0.00015 ***	
	1	101.98%	0.020 *	
9c	256	76.46%	< 0.0001 ****	
	0.5	107.27%	0.016 *	
9d	128	64.07%	0.0005 **	

	1	99.67%	0.059	
9e	32	49.64%	0.0003 ***	
	2	92.09%	0.712	
10a	128	27.55%	< 0.0001 ****	
	8	21.81%	< 0.0001 ****	
	1	111.20%	0.0012 **	
10c	256	76.05%	0.012 *	
	4	97.66%	0.046 *	
	1	103.35%	0.004 **	
10e	128	25.56%	< 0.0001 ****	1D
	4	69.90%	0.191	
	1	95.21%	0.050 *	
11a	128	29.71%	0.0013 **	
	4	55.46%	< 0.0001 ****	
	1	52.55%	< 0.0001 ****	
11b	256	18.94%	< 0.0001 ****	
	8	79.78%	0.508	
	2	77.27%	0.050 *	
11c	128	37.70%	< 0.0001 ****	
	8	56.73%	0.002 **	
	0.5	87.58%	0.832	
11e	0.5	71.68%	0.0073 **	
11f	256	30.45%	< 0.0001 ****	
	4	71.24%	0.016 *	
	0.5	89.62%	0.965	
11j	256	36.53%	< 0.0001 ****	
	0.5	90.73%	0.114	
16b	16	87.80%	0.580	1E
	1	87.59%	0.600	
16c	64	27.94%	< 0.0001 ****	
	2	72.71%	0.358	
16d	4	36.88%	0.0026 **	
	1	88.14%	0.655	
	0.5	90.7%	0.045 *	

P-value of compounds toxicity was calculated compared to the negative control.

Table S3. P-values calculated with T-test of data on Figure 2

Compound	Concentration	Survival %	P value (T-test)
10e	256	19.85%	< 0.0001 ****
	4	18.77%	< 0.0001 ****
	1	72.66%	0.091
10c	256	54.53%	0.007 **
	4	61.51%	0.196
	1	67.30%	0.832
11b	256	18.94%	< 0.0001 ****
	8	79.78%	0,508
	2	77.27%	0,050 *
11f	128	14.13%	< 0.0001 ****
	4	58.17%	0.013 *
	0.5	66.92%	0.563
Ketoconazole	256	10.50%	< 0.0001 ****
	16	67.52%	0.130
	0.5	68.51%	0.227
SeS2	256	23.49%	< 0.0001 ****
	16	18.09%	< 0.0001 ****
	0.5	43.56%	< 0.0001 ****

P-value of compounds toxicity was calculated compared to the negative control.

Table S4. P-values calculated with T-test of data on Figures 3

Compound	Concentration	Hemolysis %	P value (T-test)
3a	256	1.21%	0.036 *
	32	0.59%	0.410
	0.5	0%	0.428
3c	256	1.85%	0.0002 ***
	64	0%	0.499
5b	256	4.36%	< 0.0001 ****
	16	0%	0.003 **
	0.5	0%	0.789
7a	256	2.90%	0.004 **
	0.5	0.78%	0.154
7b	256	1.20%	0.005 **
	16	0%	0.688
	0.5	0.57%	0.405
7c	256	29.77%	< 0.0001 ****
	16	0%	0.013 *
	0.5	0%	0.103
7d	256	18.96%	< 0.0001 ****
	0.5	0%	0.586
9c	256	2.31%	< 0.0001 ****
	1	0%	0.003 **
11e	256	9.25%	< 0.0001 ****
	0.5	0%	0.135
11i	256	25.71%	< 0.0001 ****
	32	0%	0.009 **
	0.5	0.37%	0.479

P-value of compounds hemolysis was calculated compared to the negative control.
